# Ruthenium and Iron‐Catalysed Decarboxylative *N*‐alkylation of Cyclic α‐Amino Acids with Alcohols: Sustainable Routes to Pyrrolidine and Piperidine Derivatives

**DOI:** 10.1002/cssc.201901499

**Published:** 2019-07-22

**Authors:** Anastasiia Afanasenko, Rachael Hannah, Tao Yan, Saravanakumar Elangovan, Katalin Barta

**Affiliations:** ^1^ Stratingh Institute for Chemistry University of Groningen Nijenborgh 4 9747 AG Groningen The Netherlands

**Keywords:** decarboxylation, iron, *N*-alkylation, N-heterocycles, proline

## Abstract

A modular and waste‐free strategy for constructing *N*‐substituted cyclic amines via decarboxylative *N*‐alkylation of α‐amino acids employing ruthenium‐ and iron‐based catalysts is presented. The reported method allows the synthesis of a wide range of five‐ and six‐membered *N*‐alkylated heterocycles in moderate‐to‐excellent yields starting from predominantly proline and a broad range of benzyl alcohols, and primary and secondary aliphatic alcohols. Examples using pipecolic acid for the construction of piperidine derivatives, as well as the one‐pot synthesis of α‐amino nitriles, are also shown.

## Introduction

**Figure 1:**
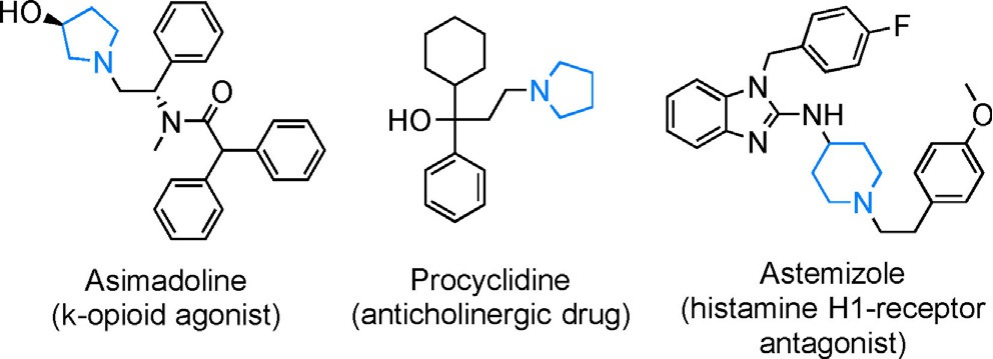
Bioactive compounds bearing *N*‐alkylated pyrrolidine and piperidine moieties.

Saturated azaheterocycles, especially pyrrolidine and piperidine, are ubiquitous scaffolds in biologically active compounds^1^ and key building blocks in diverse areas of organic chemistry.^2^ Numerous pharmaceuticals comprise five‐ and six‐membered azaheterocyclic moieties (Figure 1). Therefore, over the past decades considerable efforts have focused on the development of novel approaches for the efficient and environmentally friendly synthesis of substituted pyrrolidines and piperidines, employing affordable and widely available substrates and sustainable catalysts.

**Scheme 1:**
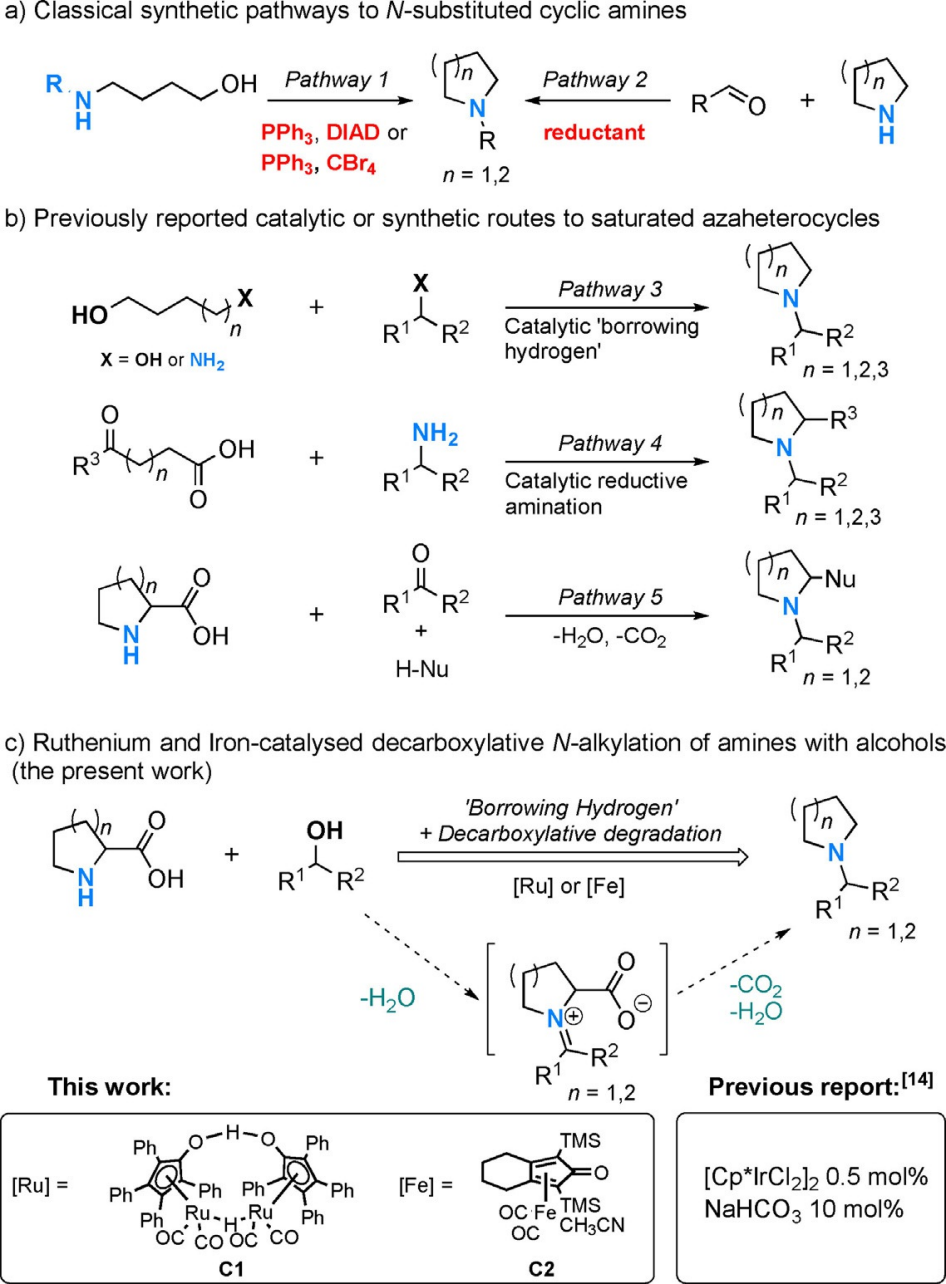
a) Classical pathways for the synthesis of the saturated azaheterocycles; b) catalytic and synthetic pathways for the construction of *N*‐alkylated heterocycles; c) Ru‐ and Fe‐catalysed decarboxylative *N*‐alkylation of amines with alcohols (this work).

Traditional methods for the construction of the core heterocyclic centre3a such as the Mitsunobu3b and Appel‐type reactions3c (Scheme 1 a, pathway 1) or classical reductive aminations3d (Scheme 1 a, pathway 2) face a number of limitations including the use of toxic solvents and/or reagents as well as poor atom economy.

The discovery of metal‐catalysed “hydrogen borrowing” methods for the *N*‐alkylation of amines with alcohols^4^ (Scheme 1 b, pathway 3) has led to cleaner synthesis of saturated azaheterocycles by judicious selection of appropriate combinations of coupling partners (e.g., benzyl amines and diols or cyclic amines with alcohols). These methods only produce water as a by‐product, may employ bio‐derived alcohol substrates^5^, ^6^ and, as recently reported, can also be performed using earth‐abundant metal catalysts.^7^, ^8^, ^9^

An elegant catalytic strategy for the construction of various cyclic amines was recently illustrated by Darcel and co‐workers (Scheme 1 b, pathway 4).^10^ They achieved the Fe‐ and Rucatalysed reductive amination of carbonyl compounds with ω‐amino fatty acids to furnish various pyrrolidines, piperidines and azepanes.

A distinctly different method for the construction of N‐heterocycles involves the decarboxylation of amino acids already containing such core moiety via coupling with carbonyl compounds (inspired by the classical Strecker degradation^11^, ^12^) followed by a reaction of the formed azomethine ylides with various non‐traditional dipolarophiles^13^ (Scheme 1 b, pathway 5).

A highly interesting approach for the one‐pot construction of valuable N‐heterocyclic scaffolds would be the combination of the hydrogen‐borrowing with the decarboxylation/azomethine ylide chemistry: this would enable the catalytic coupling of naturally abundant amino acids with widely available and potentially bio‐derived alcohol substrates (Scheme 1 c). In this case, the carbonyl compounds necessary for imine formation could be generated in situ by means of the transition‐metal catalyst while the amino acids would provide the core N‐heterocyclic scaffold upon decarboxylation. Surprisingly, to the best of our knowledge, the first and only example for such homogeneously catalysed decarboxylative *N*‐alkylation of natural α‐amino acids with alcohols has been reported by Zhao and co‐workers, employing pentamethylcyclopentadienyl iridium dichloride dimer ([Cp*IrCl_2_]_2_)/NaHCO_3_ as catalyst.^14^ Despite the potential of earth‐abundant Fe‐ or Ru‐based systems in diverse *N*‐alkylation reactions, no decarboxylative *N*‐alkylation methods have been developed yet using such catalysts. This is not surprising given the highly functionalized and potentially strongly coordinating nature of amino‐acid substrates or reaction intermediates.

Recently, we have shown that base‐free *N*‐alkylation of unprotected α‐amino acids7e with a broad range of alcohols is feasible using Shvo's catalyst and Knölker's complex and have introduced the Fe‐based catalytic *N*‐alkylation of amines with alcohols with a broad scope, including the construction of N‐heterocyclic scaffolds.7d, 7e, 8a, 8c Herein, we set to establish the first decarboxylative *N*‐alkylation methods using these Fe‐ and Ru‐based half‐sandwich complexes.

## Results and Discussion

Following our initial observations regarding the *N*alkylation of natural α‐amino acids with various alcohols using Shvo's complex (**C1**), we first selected *DL*‐proline (**1 a**) and 4‐methoxybenzyl alcohol (**2 a**) as starting materials for establishing the desired decarboxylative *N*‐alkylation methodology (Table 1). The initially selected conditions using a 1:2 molar ratio of **1 a** and **2 a**, 1 mol % **C1** at 120 °C delivered excellent results. Applying these parameters, excellent (88–99 %) product (**3 aa**) yields were detected in various solvents such as cyclopentyl methyl ether (CPME), toluene, 1,4‐dioxane, and *tert*‐amyl alcohol (entries 4–7). In chloroform (entry 8), only traces of the product were observed, whereas in acetonitrile moderate yield was seen (52 %, entry 9). While 1,4‐dioxane furnished the highest **3 aa** yield, and toluene also gave excellent results. Taking into account solvent sustainability guidelines,^15^ we chose toluene as preferred solvent for further investigation; the product yield was further improved from 90 % to 94 % using 4 equiv. of **2 a** at 120 °C (entry 4 vs entry 11). Further decrease of reaction temperature to 110 °C negatively affected the product yield (entry 10). Blank reactions in the absence of catalyst (entry 1) or in the absence of alcohol (entry 2) gave no product as expected. For comparison with our previous studies,7e the reaction was also conducted in trifluoroethanol, indeed resulting in 90 % yield of the *N*‐alkylation product without decarboxylation, presumably due to the increased acidity of the reaction medium.

**Table 1:** Establishing the decarboxylative *N*‐alkylation of *DL*‐proline with 4‐methoxybenzyl alcohol using iron‐ and ruthenium‐based catalysts.^[a]^ 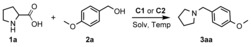

Entry	Alcohol [mmol]	Catalyst [mol %]	*T* [°C]	Solvent	Yield^[b]^ [%]
1	2	–	120	toluene	–
2	–	**C1**/1	120	toluene	–
3	1	**C1**/1	120	CF_3_CH_2_OH	96^[c]^
4	1	**C1**/1	120	toluene	90
5	1	**C1**/1	120	*tert*‐amyl alcohol	88
6	1	**C1**/1	120	CPME	89
7	1	**C1**/1	120	1,4‐dioxane	99
8	1	**C1**/1	120	CHCl_3_	traces
9	1	**C1**/1	120	CH_3_CN	52
10	2	**C1**/1	110	toluene	81
11	2	**C1**/1	120	toluene	94
12	2	**C2**/4	120	toluene	77
13	2	**C2**/8	110	toluene	75
14^[d]^	2	**C2**/4	110	toluene	71
15^[d]^	2	**C2**/4	110	1,4‐dioxane	73
16	2	**C2**/4	120	1,4‐dioxane	70

[a] General reaction conditions: 0.5 mmol of **1**, 1 or 2 mmol of **2**, 1 mol % **C1** or 4–8 mol % **C2**, 2 mL of solvent, 24 h, 100–120 °C, under argon. [b] Isolated yields. [c] *N*‐alkylated non‐decarboxylated product was observed. [d] 48 h.

Next, we turned our attention to the Fe catalyst **C2**.^16^ Applying **C2** in the model reaction at 120 °C in toluene 77 % yield was achieved (entry 12) within 24 h. However, further attempts to enhance **3 aa** yield either by doubling the catalyst loading to 8 mol % or by increasing the reaction time to 48 h did not significantly influence the yield of the reaction (75 % and 71 %, entries 13 and 14, respectively). The lower yield achieved with **C2** compared to **C1** could be attributed to a slower dehydrogenation and/or a slower reduction step involved in the hydrogen‐borrowing cycle (see also Scheme 3).

In further studies, the scope and limitations of the newly established Ru‐ and Fe‐catalysed decarboxylative *N*‐alkylation methodologies were explored. Benzyl alcohol with electrondonating substituents such as 4‐methoxybenzyl alcohol (**2 a**) and 4‐methylbenzyl alcohol (**2 b**) as well as bulky substituents (**2 c**, **2 d**) were successfully coupled with **1 a**, furnishing the corresponding products (**3 aa**, **3 ab**, **3 ac**, **3 ad**) with excellent isolated yields (86–94 %) with **C1** as catalyst, whereas poorer yields (19–77 %) were obtained when applying the Fe‐based catalyst **C2** (Table 2, entries 1–4). Interestingly, piperonyl alcohol (**2 e**) gave excellent isolated yields (94 %) of **3 ae** in both systems (entry 5). With 3‐pyridine methanol (**2 g**) and 2‐thiophene methanol (**2 h**), moderate product yields were observed with **C1**, while reactivity was completely blocked using **2 g** with **C2**. The latter system appeared more compatible with **2 h** (33 % **3 ah** with **C1** vs. 45 % with **C2**).

**Table 2:** Decarboxylative *N*‐alkylation of amino acids with primary alcohols.^[a]^ 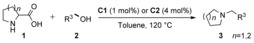

Entry	Product		Yield^[b]^ [%]	
			**C1**	**C2**
1	**3 aa**	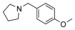	94	77
2	**3 ab**	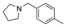	86	37
3	**3 ac**	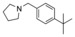	93	75
4	**3 ad**	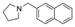	91	19
5	**3 ae**	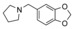	94	94
6	**3 af**	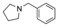	42	58
7	**3 ag**	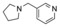	40	–
8	**3 ah**	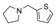	33	45
9	**3 ai**	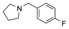	75	26
10	**3 aj**	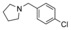	90	68
11	**3 ak**	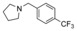	84	37
12	**3 al**	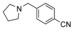	83	18
13	**3 am**	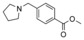	65	14
14	**3 an**	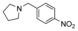	99	traces
15	**3 ao**	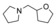	83^[c]^	–
16	**3 ap**	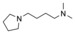	86^[c]^	–
17	**3 ba**	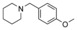	31^[c]^	54
18	**3 bn**	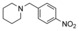	64	–

**[**a] General reaction conditions: 0.5 mmol of **1**, 1 mmol of **2**, 1 mol % **C1** or 4 mol % **C2**, 2 mL toluene, 24 h, 120 °C, under argon. [b] Isolated yields. [c] Yields are based on ^1^H NMR spectroscopy, using 1,3,5‐trimethoxybenzene as an internal standard.

When *para*‐halide‐substituted benzyl alcohols were employed, 75 % of **3 ai** and 90 % of **3 aj** were successfully isolated. Moreover, desired products bearing deactivating functional groups such as −CF_3_, −CN, −CH_3_COOCH_3_ and −NO_2_ were formed in good‐to‐excellent isolated yields (65–99 %, **3 ak**–**3 an**). Considerably lower yields were observed for the above‐mentioned substrates using **C2** as catalyst (14–37 %, **3 ak**–**3 an**). Selected aliphatic alcohols such as **2 o** and **2 p** reacted smoothly with *DL*‐proline, affording **3 ao** and **3 ap** in very good yields (83–86 %), albeit only with **C1** as catalyst (entries 15 and 16).

Notably, the developed methodology could be extended to pipecolic acid (**1 b**) as well, and the target *N*‐substituted piperidines **3 ba**–**3 bn** were obtained in 31 and 64 % isolated yields, respectively, using **C1**. In the case of product **3 ba**, the Fe‐based catalyst (**C2**) gave a 54 % isolated yield.

Other acyclic α‐ and β‐amino acids (glycine, *DL*‐alanine, *DL*‐phenylalanine, β‐alanine) as well as *N*‐alkyl α‐amino acids (*N*‐methyl glycine, *N*‐isopropyl valine) were attempted to couple with 4‐methoxybenzyl alcohol (**2 a**) under the optimized reaction conditions; however, low yields were obtained in both systems (using **C1** and **C2**). Additionally, employing **C1** as a catalyst we examined reactions between *N*‐methyl glycine (sarcosine)/*N*‐isopropyl valine and **2 a** with the addition of a base (KO*t*Bu, KOH, K_2_CO_3_, NaHCO_3_) in various solvents (1,4‐dioxane, *t*‐amyl alcohol, CPME); however, no significant improvement in product yield could be established.

To further expand the scope of the reaction, we turned our attention to the use of secondary and long‐chain primary aliphatic alcohols applying the more active Ru‐based catalytic system (Table 3). Cyclohexanol (**2 q**) and 4‐isopropylhexan‐1‐ol (**2 r**) smoothly reacted with **1 a**, providing good yields (57 % and 76 %) of the corresponding products (**3 aq**, **3 ar**, respectively). However, no product was observed in the reaction of *DL*‐proline with menthol (**2 s**), presumably due to the steric bulk of the alcohol substrate. Similarly, other secondary alcohols such as **2 t**, **2 u**, and **2 v** furnished the desired products in reasonable yields. Interestingly, the use of cinnamyl alcohol **2 w** or **2 x** led to the formation of products **3 aw** and **3 ax** in good yields, although the double bond of **3 aw** was found to be reduced. Several long‐chain aliphatic alcohols were also found to react with **1 a** and **1 b**, affording the corresponding products (**3 ay**, **3 by**) in 54 % and 55 % isolated yields, respectively.

**Table 3:** Decarboxylative *N*‐alkylation of amino acids with secondary and long‐chain alcohols.^[a]^ 
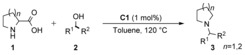

Entry	Product		Yield^[b]^ [%]
1	**3 aq**	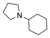	57
2	**3 ar**	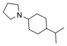	76^[c]^
3	**3 as**	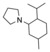	0
4	**3 at**	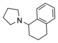	49
5	**3 au**	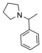	62
6	**3 av**	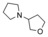	42^[c]^
7	**3 aw**	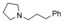	81^[d]^
8	**3 ax**	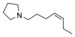	51^[c]^
9	**3 ay**	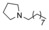	54
10	**3 by**	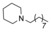	55

[a] General reaction conditions: 0.5 mmol of **1**, 1 mmol of **2**, 1 mol % **C1**, 2 mL toluene, 24 h, 120 °C, under argon. [b] Isolated yields. [c] Yields are based on ^1^H NMR spectroscopy, using 1,3,5‐trimethoxybenzene as an internal standard. [d] Reduced double bond in the product (note: 2 equiv. of alcohol used).

**Scheme 2:**
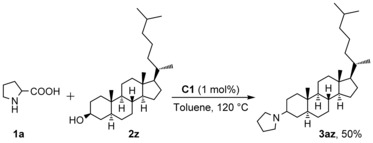
Decarboxylative *N*‐alkylation of *DL*‐proline with 5α‐cholestan‐3β‐ol.

Encouraged by the above results showing a broad range of alcohols suitable for the Ru‐catalysed decarboxylative *N*‐alkylation of α‐amino acids, we investigated the possibility of employing 5α‐cholestan‐3β‐ol as substrate for the decarboxylative *N*‐alkylation of *DL*‐proline (Scheme 2). The corresponding product was successfully obtained in 50 % isolated yield, which demonstrates the applicability of the developed methodology for the functionalization of biologically active compounds.

Next, we investigated the use of mandelonitriles (**4**) as unique substrates for the formation of nitriles and the possibility of accessing cyclic amino nitriles in a straightforward and one‐pot approach based on mechanistic considerations present in literature.^17^ Notably, the use of mandelonitrile (**4 a**) and its derivatives 4‐methylmandelonitrile (**4 b**) and 4‐chloromandelonitrile (**4 c**) led to the formation of products **5 a**, **5 b** and **5 c** as well as their regioisomers **5′a**, **5′b** and **5′c** in good combined isolated yields (71–76 %) when applying both catalytic systems **C1** and **C2** (Table 4). A preliminary proposal for the formation of these regioisomers is provided (Supporting Information, Figure S5, Note 1). Basic *DL*‐proline, used in slight excess in the system, is assumed to play a role in α‐aminonitrile isomerization in favour of regioisomer **5** (Supporting Information, Figure S5, Note 1).17d This is supported by the fact that **5:5′** ratios followed a specific trend (Table 4) related to the nature of the substituents on the aromatic ring, where electron‐withdrawing substituents would stabilize dipoles with a partial negative charge predominantly in the benzylic position. Thus, regioselectivity of the reaction could be attributed to the unlike charge distribution of the differently substituted azomethine ylides.13c

**Table 4:** Construction of α‐amino nitriles from mandelonitrile and its derivatives using an *N*‐alkylation/decarboxylation strategy.^[a]^ 
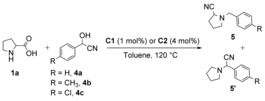

Entry	Major product (**5**)		Ratio	Yield^[b]^ [%]	
			**5:5′**	**C1**	**C2**
1	**5 a**+**5′a**	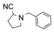	7:1	71	71
2	**5 b**+**5′b**	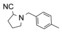	3:1	74	72
3	**5 c**+**5′c**	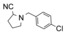	11:1	75	76

[a] General reaction conditions: 1.1 mmol of **1 a**, 1 mmol of **4**, 1 mol % **C1** or 4 mol % **C2**, 2 mL toluene, 24 h, 120 °C, under argon. [b] Reported value refers to combined isolated yield of both regioisomers **5** and **5′**.

**Scheme 3:**
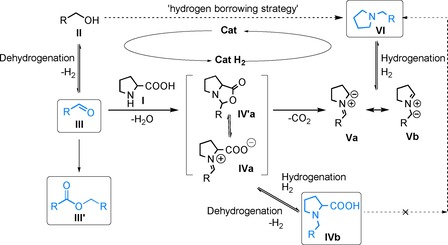
Proposed mechanism for the decarboxylative *N*‐alkylation of α‐amino acids with alcohols. Structures in boxes detected by in situ 1D and 2D NMR spectroscopy.

A plausible mechanism of the decarboxylative *N*‐alkylation of α‐amino acids with alcohols is depicted in Scheme 3. The proposed reaction sequence is based on control experiments and 1D, 2D NMR spectroscopic investigations as well as earlier literature reports13a, 14 (further specified below). The sequence begins with the metal‐catalysed dehydrogenation of an alcohol (**II**) to the carbonyl compound (**III**), which readily undergoes condensation with an amino acid (**I**; here, *DL*‐proline). Condensation and water elimination results in the formation of an oxazolidin‐5‐one derivative (**IV′a**), which is in equilibrium with the acyclic iminium carboxylate intermediate (**IVa**). The formation of oxazolidones by reaction of α‐amino acids with carbonyl compounds was previously extensively investigated by Grigg et al.17b and Seebach et al.^18^ whereas oxazolidone/iminium equilibria were proposed during mechanistic studies of proline‐mediated aldol condensation reactions.^19^ Decarboxylation of thermally labile oxazolidin‐5‐one derivative (**IV′a**) furnishes azomethine ylides (**Va** and **Vb**) in a dipolar [3+2] cycloreversion step,^20^, ^21^ as also discussed in literature for various oxazolidinone derivatives.17b, 18 As a final step, the azomethine ylides would undergo protonation and subsequent reduction by means of the metal hydride generated during the first dehydrogenation step, leading to the formation of the *N*‐alkylated cyclic amine product (**VI**). The existence of a dehydrogenation step (Scheme 3, **VI** to **Va** and **Vb**) through involvement of a Ru‐ and Ir‐based transfer hydrogenation catalyst similar to **C1** was previously described in literature.9b, 9c

Several control experiments were conducted to confirm the central role of the catalyst (**C1**) in the proposed reaction scheme. As discussed above, no reaction occurred between the alcohol and amino acid in the absence of **C1** (entry 1, Table 1) and **C1** did not induce decarboxylation of the α‐amino acid in the absence of the alcohol (entry 2, Table 1).

Next, 1D and 2D NMR spectroscopic investigations of a reaction mixture containing 4‐methoxybenzyl alcohol (**2 a**) and **1 a** in [D_8_]toluene at 120 °C in the presence of Shvo's catalyst (**C1**) were conducted to confirm several proposed reaction intermediates (Supporting Information, Figures S1–S4). These studies allowed the detection of key intermediate 4‐methoxybenzaldehyde (**III**), and the ester (**III′**) as side product originating from the aldehyde (Supporting Information, Figure S2). Although we were not able to detect the oxazolidin‐5‐one derivative (**IV′a**) or the acyclic iminium carboxylate species (**IVa**), the corresponding *N*‐alkyl‐proline (**IVb**) formed by hydrogenation of (**IVa**)—via the metal hydride generated by dehydrogenation—was observed as indirect evidence (Supporting Information, Figure S3). The presence of the Ru−H species expected in the catalytic cycle was also affirmed by its typical distinct chemical shift (−9.65 ppm, Supporting Information, Figure S4).

More experiments were conducted to further elaborate on the role of intermediate **IVb**. A direct decarboxylation pathway starting from separately synthetized benzyl‐pyrrolidine‐2carboxylic acid (**IVb**) under the reaction conditions but in absence of **C1** could be ruled out. However, excellent yield (>99 %) of the target cyclic amine (**VI**) was achieved starting from (**IVb**) in the presence of (**C1**), confirming the proposed hydrogenation/dehydrogenation equilibrium between **IVa** and **IVb**. Indeed, **C1** was previously shown to efficiently catalyse imine hydrogenation as well as the dehydrogenation of secondary or tertiary amines.^22^, ^23^ This result is particularly interesting, since, to the best of our knowledge, the decarboxylation of *N*‐alkyl amino acids into their *N*‐alkylamine analogues has not yet been accomplished by using a dehydrogenation catalyst.

Further, aiming to prove that the alcohol is a genuine hydrogen source, **1 a** was allowed to react with α,α‐[D_2_]benzyl alcohol (**2 f–d2**) under standard reaction conditions. The product distribution analysis employing ^1^H NMR spectroscopy (Supporting Information, Figure S6, Note 2) displayed deuterium incorporation at the 2 and 5 positions of the pyrrolidine ring as well as at the benzylic position of the desired product, which additionally supported the above‐proposed mechanism (for discussion see the Supporting Information, Note 2).

Lastly, during the 1D and 2D NMR spectroscopic investigations, where significant amount of product was detected already after 1 h reaction time, it became apparent that the reaction proceeds faster than initially assumed based on our earlier studies that frequently displayed sluggish imine hydrogenation step.7d, 7e, 8a, 8c Therefore we have followed the evolution of detectable intermediates (**III**, **IVb**, **Ru‐H**) and product (**VI**) over time (Supplementary Figure S7, Note 3). Gratifyingly, already after 2 h full conversion and excellent product yield was achieved, confirmed by an isolated yield of 96 % for 1‐(4‐methoxybenzyl)pyrrolidine (**3 aa**). Although certainly substrate dependent, at 120 °C the decarboxylation of the thermally labile oxazolidin‐5‐one derivative to the proposed azomethine ylides is expected to be rapid; hence, it appears that the proposed hydrogen transfer from the substrate alcohol to the ylides is facile as well. This presents a unique advantage of the method presented herein.

## Conclusions

We have developed the decarboxylative *N*‐alkylation of α‐amino acids with alcohols applying Ru‐ and Fe‐based catalytic systems for the synthesis of *N*‐substituted cyclic amines. The described methods demonstrate high selectivity, wide alcohol scope and excellent functional‐group tolerance, in particular regarding the Ru‐based system. Although the iron‐based method would require further optimization in terms of efficiency possibly by switching to alternative catalyst structures capable of borrowing hydrogen, the proof of principle presented here opens the way toward fully sustainable methodologies for the construction of saturated azaheterocycles since both the α‐amino acid as well as the alcohol substrates can be obtained from renewable resources and the employed catalyst uses an earth‐abundant, non‐toxic metal.

## Experimental Section

### General procedure for the decarboxylative *N*‐alkylation of amino acids

An oven‐dried 20 mL Schlenk tube, equipped with a stirring bar, was charged with amino acid (0.5 mmol, 1 equiv.), corresponding alcohol (1 or 2 mmol, 2 or 4 equiv.), Shvo's catalyst (**C1**, 0.005 mmol, 1 mol %) or Knölker's complex (**C2**, 0.02 mmol, 4 mol %) and toluene (as a solvent, 2 mL). Solid materials were weighed into the Schlenk tube under air. Then the Schlenk tube was subsequently connected to an argon line and vacuum–argon exchanges were performed three times. Liquid starting materials and solvent were charged under an argon stream. The Schlenk tube was capped, and the mixture was rapidly stirred at room temperature for 1 min, then was placed into a pre‐heated oil bath at 120 °C and stirred for a given time (typically, 24 h). Then, the reaction mixture was cooled down to room temperature, the crude mixture was filtered through silica gel, eluted with ethyl acetate and concentrated in vacuo. The residue was purified by flash column chromatography to provide the pure amine product.

## Conflict of interest

*The authors declare no conflict of interest*.

## Supporting information

As a service to our authors and readers, this journal provides supporting information supplied by the authors. Such materials are peer reviewed and may be re‐organized for online delivery, but are not copy‐edited or typeset. Technical support issues arising from supporting information (other than missing files) should be addressed to the authors.

Supplementary
