# Supplementary material for: Ruthenium and Iron‐Catalysed Decarboxylative N‐alkylation of Cyclic α‐Amino Acids with Alcohols: Sustainable Routes to Pyrrolidine and Piperidine Derivatives
Source: ChemSusChem. 2019 Jul 22;12(16):3801–7. doi: 10.1002/cssc.201901499 (PMC6772061; doi:10.1002/cssc.201901499)
Supplement: Supplementary file 1 — Supplementary [file CSSC-12-3801-s001.pdf]

CHEMISTRY & SUSTAINABILITY
CHEMSUSCHEM
ENERGY & MATERIALS

Supporting Information

Ruthenium and Iron-Catalysed Decarboxylative *N*-alkylation of Cyclic α-Amino Acids with Alcohols: Sustainable Routes to Pyrrolidine and Piperidine Derivatives

Anastasiia Afanasenko, Rachael Hannah, Tao Yan, Saravanakumar Elangovan, and Katalin Barta*[a]

cssc_201901499_sm_miscellaneous_information.pdf

Table of contents

General methods 3
Chromatography and spectroscopy 3
Representative procedures 3
General procedure for the decarboxylative *N*-alkylation of amino acids 3
Supplementary figures 4
^{1}H and ^{13}C NMR spectra 17
References 43

General methods

Chromatography and spectroscopy

Column chromatography was performed using Merck silica gel type 9385 230-400 mesh and typically pentane and ethyl acetate or ethyl acetate and methanol as eluent.

TLC: Merck silica gel 60, 0.25 mm. The components were visualized by UV or $KMnO_4$ staining.

Gas Chromatography was used for product identification as well as determination of conversion and selectivity values. Product identification was performed by GC-MS (Shimadzu QP2010 Ultra) with an HP-1MS column, and helium as carrier gas. GC-MS method: The temperature program started at 50 °C for 5 min, heated by 30°C/minute to 250 °C and held for 15 min.

Mass spectrometry: Mass spectra were recorded on an AEI-MS-902 mass spectrometer (EI^+) or a LTQ Orbitrap XL (ESI^+).

NMR spectroscopy: ^1H and ^{13}C NMR spectra were recorded on a Varian Mercury Plus 400, Agilent MR 400 (400 and 101 MHz, respectively) and Bruker Avance NEO 600 (600 and 151 MHz, respectively) using $CDCl_3$, CD_3OD, or Toluene-d_8 as a solvent. ^1H and ^{13}C NMR spectra were recorded at room temperature. Chemical shift values are reported in ppm with the solvent resonance as the internal standard ($CDCl_3$: 7.26 for ^1H, 77.00 for ^{13}C; CD_3OD: 3.31 for ^1H, 49.00 for ^{13}C; Toluene-d_8: 7.09, 7.01, 6.97, 2.08 for ^1H, 137.48, 128.87, 127.96, 125.13, 20.43 for ^{13}C.). Data are reported as follows: chemical shifts, multiplicity (s = singlet, d = doublet, t = triplet, q = quartet, br. = broad, m = multiplet), coupling constants (Hz), and integration.

All reactions were carried out under an Argon atmosphere using oven (120 °C) dried glassware and using standard Schlenk techniques. 1-Hydroxytetraphenylcyclopentadienyl-(tetraphenyl-2,4-cyclopentadien-1-one)-μ-hydrotetracarbonyldiruthenium(II) (Shvo's catalyst, **C1**) was purchased from Strem Chemicals, Inc. Complex **C2** was synthesized according to the literature procedure.[1] All other reagents were purchased from Sigma-Aldrich, Acros and TCI in reagent or higher grade and were used as received without further purification.

Representative procedures

General procedure for the decarboxylative *N*-alkylation of amino acids

An oven-dried 20 mL Schlenk tube, equipped with a stirring bar, was charged with amino acid (0.5 mmol, 1 equiv.), corresponding alcohol (1 or 2 mmol, 2 or 4 equiv.), Shvo's catalyst (**C1**, 0.005 mmol, 1 mol%) or Knölker's complex (**C2**, 0.02 mmol, 4 mol%), and toluene (as a solvent, 2 mL). Solid materials were weighed into the Schlenk tube under air. Then the Schlenk tube was subsequently connected to an argon line and vacuum-argon exchanges were performed three times. Liquid starting materials and solvent were charged under an argon stream. The Schlenk tube was capped and the mixture was rapidly stirred at room temperature for 1 min, then was placed into a pre-heated oil bath at 120 °C and stirred for a given time (typically, 24 h). Then, the reaction mixture was cooled down to room temperature, the crude mixture was filtered through silica gel, eluted with ethyl acetate, and concentrated *in vacuo*. The residue was purified by flash column chromatography to provide the pure amine product.

Supplementary figures

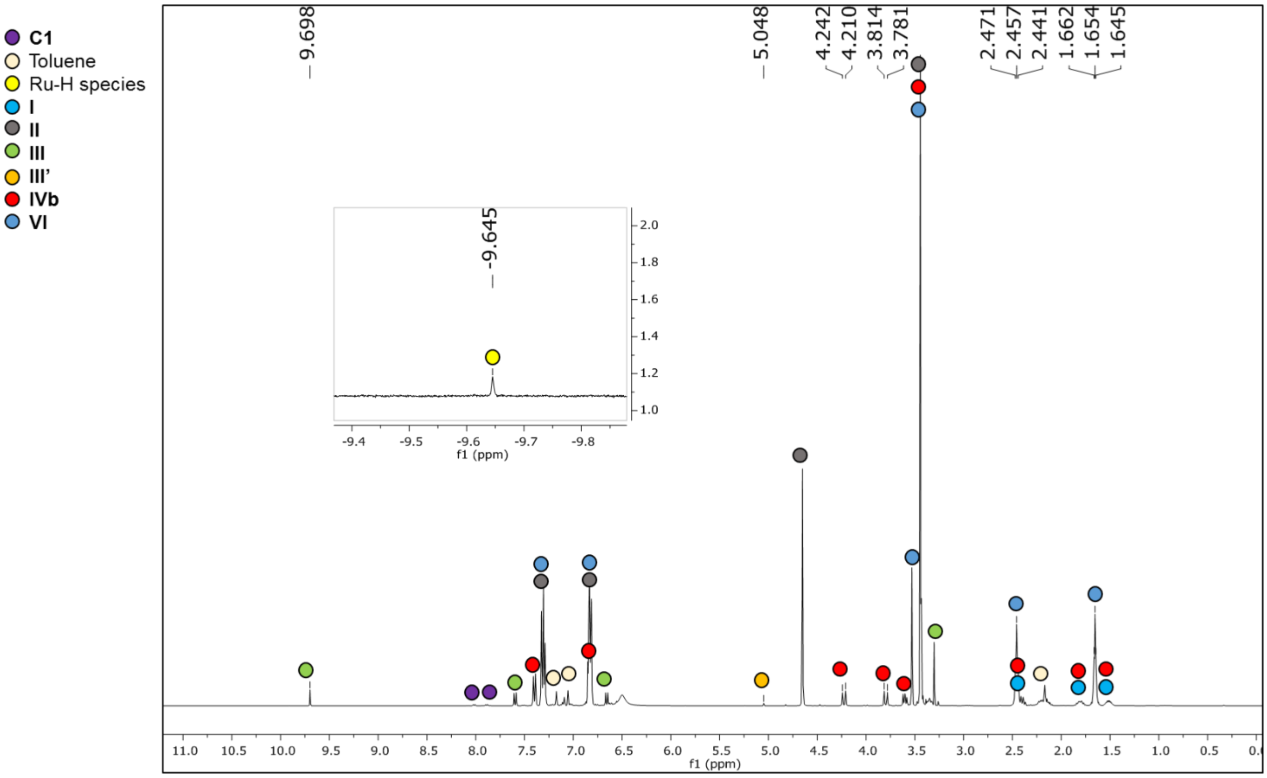

Supplementary Figure 1: *In situ* ^{1}H NMR study of the decarboxylative *N*-alkylation of *DL*-proline (**1a**, 0.5 mmol) with 4-methoxybenzyl alcohol (**2a**, 1 mmol) in presence of Shvo's catalyst (**C1**, 0.005 mmol) in toluene-d$_8$ at 120 °C after 1 hours.

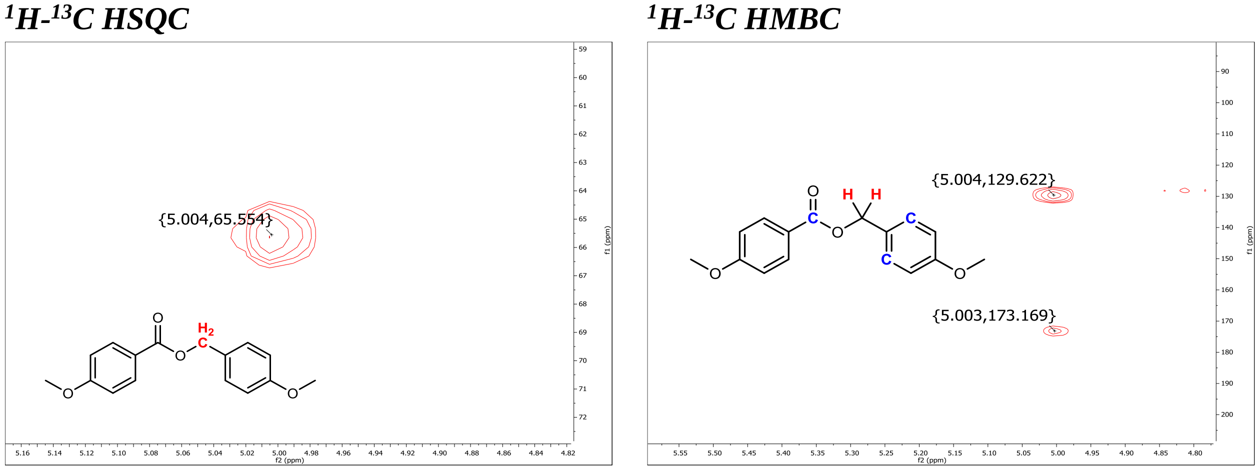

Supplementary Figure 2: Evidence of the side product (**III'**) formation according to the {^{1}H-^{13}C}HSQC (**A**) and {^{1}H-^{13}C}HMBC (**B**) 2D NMR analysis.

^{1}H-^{13}C HSQC

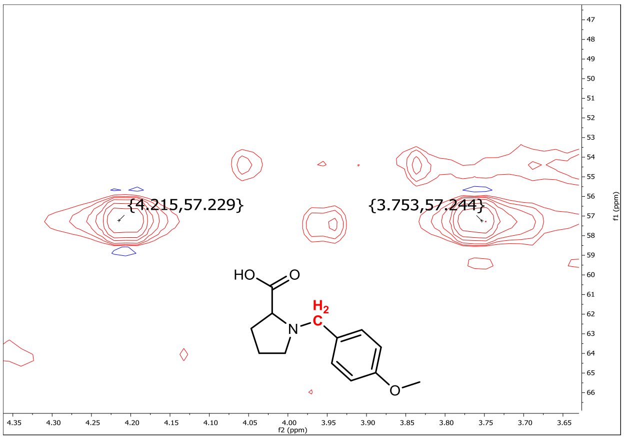

^{1}H-^{13}C HMBC

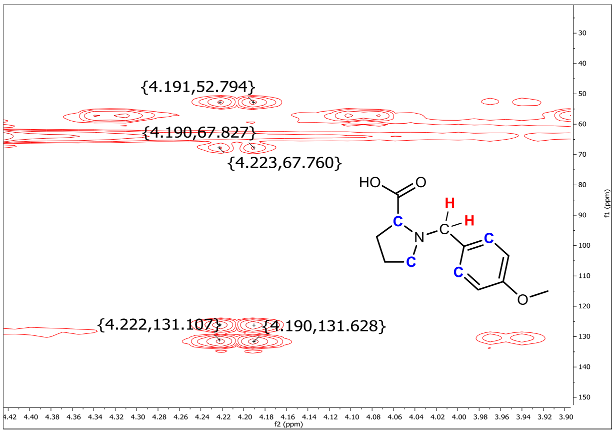

Supplementary Figure 3: Evidence of the acyclic intermediate formation (**IVb**) according to the {^{1}H-^{13}C}HSQC (**A**) and {^{1}H-^{13}C}HMBC (**B**) 2D NMR analysis.

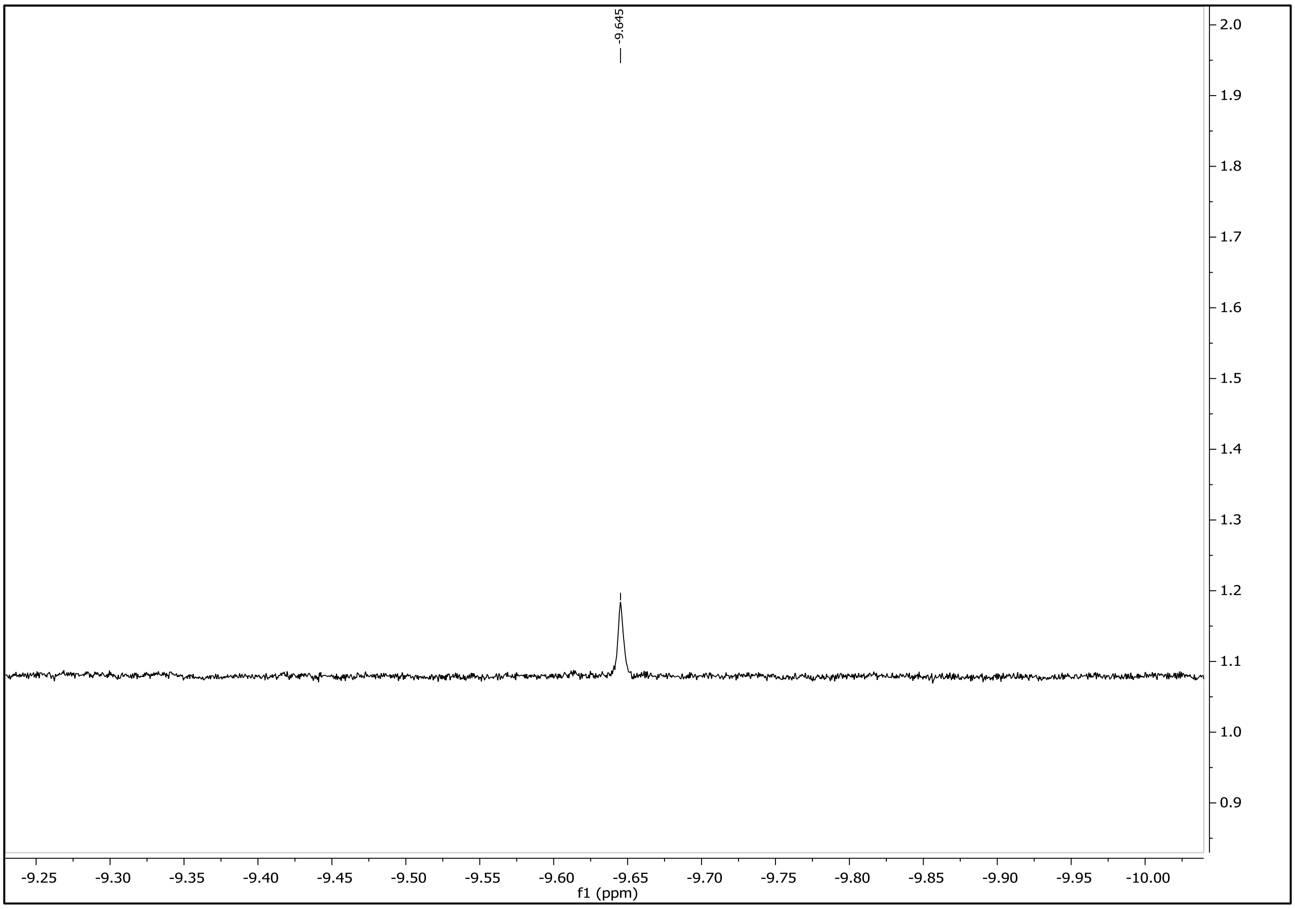

Supplementary Figure 4: Evidence of the Ru-H species formation.

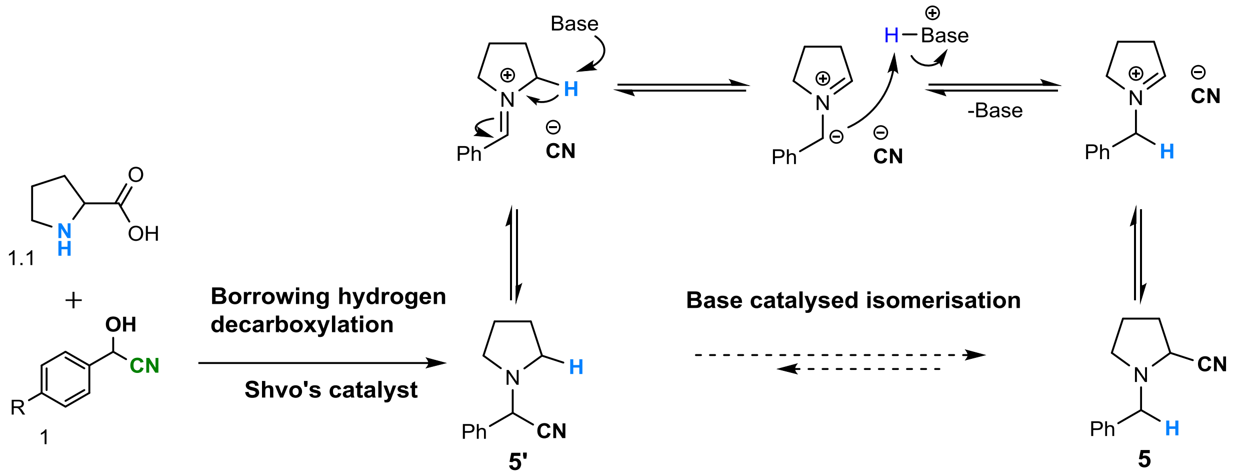

Supplementary Figure 5: Formation of α-aminonitrile by unique decarboxylative *N*-alkylation (for proposed mechanism see **Figure 4**) followed by base-catalysed α-aminonitrile isomerization as proposed by Siedel (*J. Am. Chem. Soc.* **2012**, *134*, 15305–15308).

Note 1: Additional description belonging to **Supplementary Figures 5**
We rationalise the formation of aminonitrile regioisomer **5'** *via* the hydrogen borrowing/ decarboxylative degradation sequence when mandelonitrile substrates are used as carbonyl precursors (**Table 4,** main text), in accordance with our general mechanistic proposal (**Figure 4**). Isomerisation of α-aminonitrile **5'** to **5** have been previously investigated and described by Siedel.[2,3] Due to the excess of proline in our system, we assume analogous isomerisation pathways to take place *via* an azomethine ylide intermediate (**Supplementary Figure 5**). The formation of the two regioisomers would depend on the charge distribution in the azomethine ylide. Li and co-workers have investigated the energy of resonance structures of the corresponding ylides leading to **5** and **5'** in favour of regioisomer **5,**[2] which perfectly matches with the obtained experimental data in our work (**Table 4**).
Alternative pathways, for example the loss of HCN upon reaction of proline with the carbonyl compound formed by dehydrogenation of the mandelonitrile substrate, followed by subsequent direct decarboxylation to the corresponding azomethine ylide would also be possible.

Note 2: Deuterium incorporation experiment was carried out in the following way:
An oven-dried 20 mL Schlenk tube, equipped with a stirring bar, was charged with *DL*-proline (**1a**, 0.1 mmol, 1 equiv.), benzyl alcohol-α,α-d_2 (**2f-d2**, 0.2 mmol, 2 equiv.), Shvo's catalyst (**C1**, 0.001 mmol, 1 mol%), and toluene-d_8 (as a solvent, 0.4 mL). Solid materials were weighed into the Schlenk tube under air. Then the Schlenk tube was subsequently connected to an argon line and vacuum-argon exchanges were performed three times. Liquid starting materials and solvent were charged under an argon stream. The Schlenk tube was then capped and the mixture was rapidly stirred at room temperature for 1 min, then was placed into a pre-heated oil bath at 120 °C and stirred for 2 h.

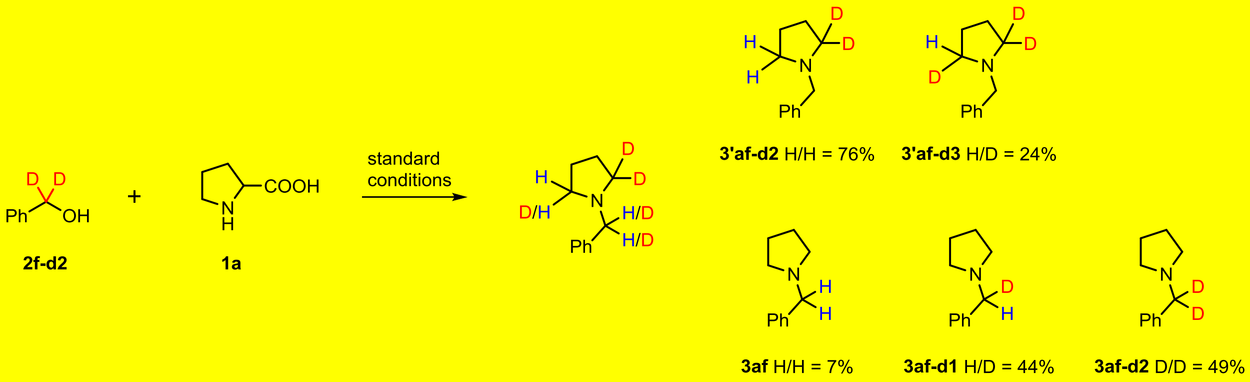

The deuterium is transferred from the benzylic position of the alcohol substrate (**2f-d2**) to the product confirms that the reaction indeed proceeds through a hydrogen borrowing pathway. The deuterium was incorporated at the *α*-position to the nitrogen at the endocyclic position and/or the benzylic position of the product, confirming that the azomethine ylide tautomerises during the reaction. Multiple deuterium atoms can be found in the products shown in **Supplementary Figure 6.** An additional reason for deuterium incorporation could be the existence of a hydrogenation/dehydrogenation equilibrium between **IVa** and **IVb** as well as between **Vl** and **Va/Vb** (**Figure 4** in the main text). Such dehydrogenation step with analogous substrates was already proposed in literature (C. Bruneau, *J. Am. Chem. Soc.* 2011, *133*, 10340–10343) using similar Ru transfer hydrogenation catalyst.

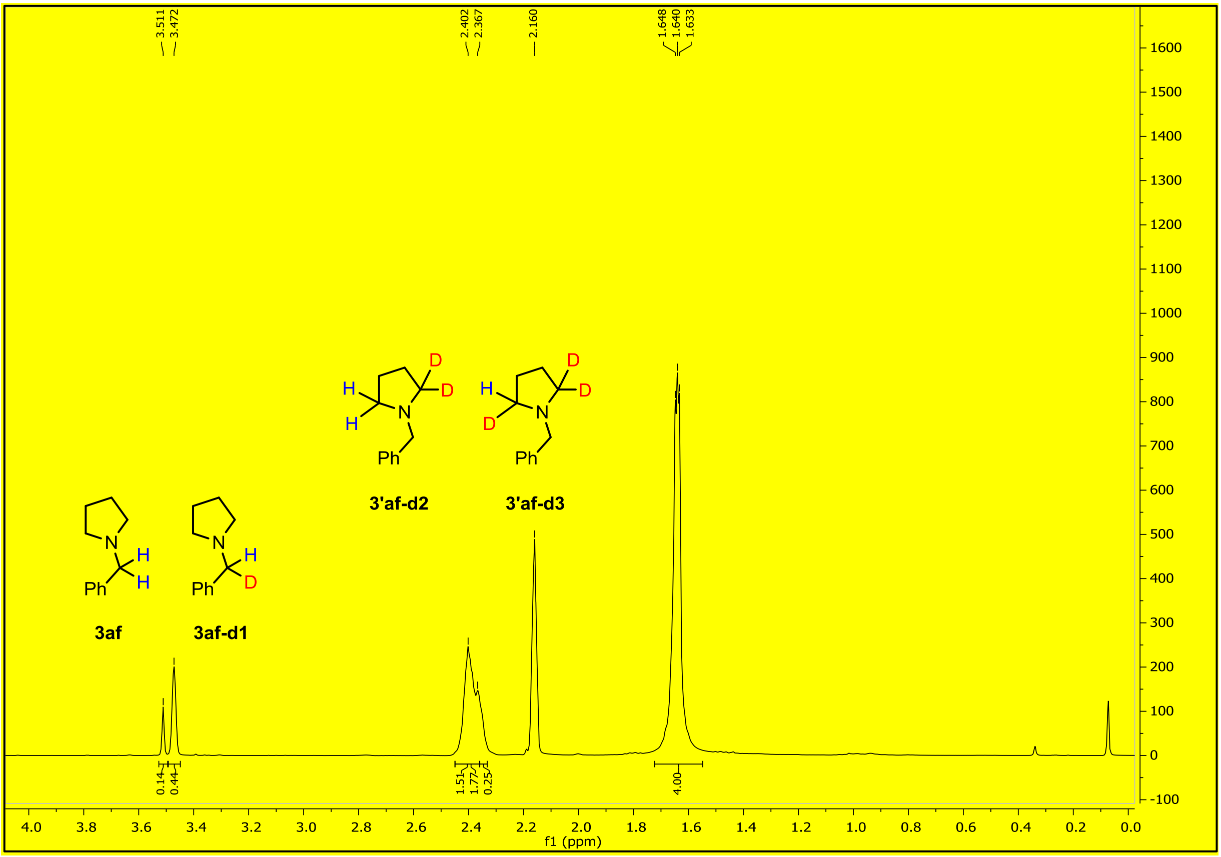

Supplementary Figure 6: ^{1}H NMR spectrum of the depicted on scheme above deuterium incorporation reaction.

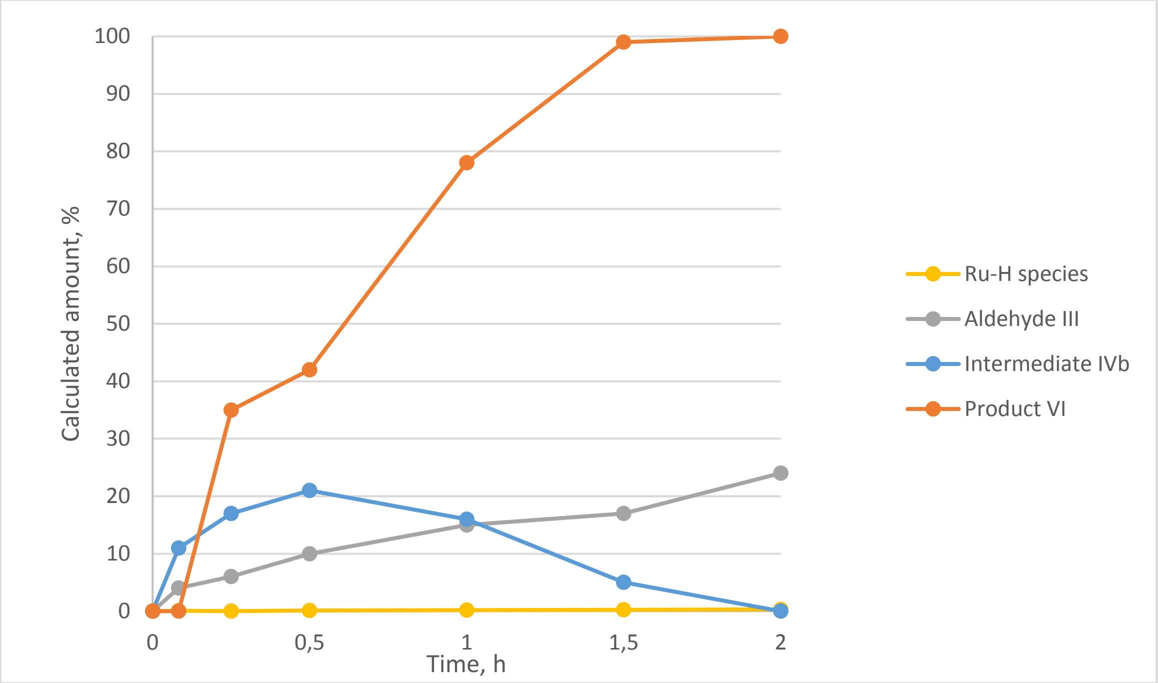

Supplementary Figure 7: The plot of selectivity of the intermediate/product formation *vs* reaction time in the reaction between *p*-methoxybenzyl alcohol (**2a**) and *DL*-proline (**1a**) in presence of Shvo's catalyst (**C1**).

Note 3: Kinetic experiments were carried out in the following way:

An oven-dried 20 mL Schlenk tube, equipped with a stirring bar, was charged with *DL*-proline (0.3 mmol, 1 equiv.), *p*-methoxybenzyl alcohol (0.6 mmol, 2 equiv.), Shvo's catalyst (**C1**, 0.003 mmol, 1 mol%), and toluene-d_8 (as a solvent, 1 mL). Solid materials were weighed into the Schlenk tube under air. Then the Schlenk tube was subsequently connected to an argon line and vacuum-argon exchanges were performed three times. Liquid starting materials and solvent were charged under an argon stream. The Schlenk tube was capped and the mixture was rapidly stirred at room temperature for 1 min, then was placed into a pre-heated oil bath at 120 °C and stirred for a specified time (5 min, 15 min, 30 min, 1h, 1h 30 min, 2 h). All reactions were set up in parallel. Then, the reaction mixture was cooled down to room temperature and 1,3,5-trimethoxybenzene (0.1 mmol, 0.3 equiv.) as an internal standard was added. Preparing the sample, 0.6 mL of the reaction mixture was placed to a *J*-Young NMR tube under argon. All spectra were recorded using Bruker Avance NEO 600 machine.

The obtained data is summarized in the table below.

Entry	Time, h	Selectivity, %			
		**III**	**IVb**	**VI**	Ru-H species
1	0.08	4	11	0	0.08
2	0.25	6	17	35	0.08
3	0.5	10	21	42	0.1
4	1	15	16	78	0.16
5	1.5	17	5	99	0.23
6	2	24	0	100	0.3

Spectral data of isolated compounds

1-(4-Methoxybenzyl)pyrrolidine (3aa)

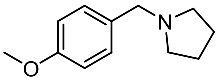

The compound was synthesised according to the **General procedure**. *DL*-Proline (57.5 mg, 0.5 mmol) affords **3aa** (90 mg, 94% using **C1**; 73 mg, 77% using **C2**). Colorless oil was obtained after column chromatography (SiO_2, EtOAc/MeOH 70:30). **^{1}H NMR** (400 MHz, $CDCl_3$): δ 7.25 (d, *J* = 8.4 Hz, 2H), 6.85 (d, *J* = 8.8 Hz, 2H), 3.79 (s, 3H), 3.57 (s, 2H), 2.52-2.49 (m, 4H), 1.80-1.77 (m, 4H). **^{13}C NMR** (151 MHz, $CDCl_3$): δ 158.79, 130.48, 130.25, 113.68, 59.79, 55.25, 53.87, 23.38. **HRMS** ($APCI^+$, m/z) calculated for $C_{12}H_{17}NO$ $[M+H]^+$: 192.13829; found: 192.13791. The spectral data are identical to the previously reported.[4]

1-(4-Methylbenzyl)pyrrolidine (3ab)

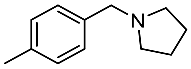

The compound was synthesised according to the **General procedure**. *DL*-Proline (57.5 mg, 0.5 mmol) affords **3ab** (75 mg, 86% using **C1**; 32 mg, 37% using **C2**). Yellow oil was obtained after column chromatography (SiO_2, EtOAc/MeOH 90:10). **^{1}H NMR** (400 MHz, $CDCl_3$): δ 7.24 (d, *J* = 7.6 Hz, 2H), 7.13 (d, *J* = 8.0 Hz, 2H), 3.63 (s, 2H), 2.57-2.54 (m, 4H), 2.34 (s, 3H), 1.82-1.78 (m, 4H). **^{13}C NMR** (101 MHz, $CDCl_3$): δ 139.22, 138.24, 131.66, 131.59, 62.78, 56.51, 26.05, 23.76. **HRMS** ($APCI^+$, m/z) calculated for $C_{12}H_{18}N$ $[M+H]^+$: 176.14338; found: 176.14316.

1-(4-(tert-butyl)benzyl)pyrrolidine (3ac)

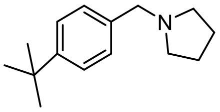

The compound was synthesised according to the **General procedure**. *DL*-Proline (57.5 mg, 0.5 mmol) affords **3ac** (101 mg, 93% using **C1**; 82 mg, 75% using **C2**). Slightly yellow oil was obtained after column chromatography (SiO_2, EtOAc/MeOH 70:30). **^{1}H NMR** (400 MHz, CD_3OD): δ 7.35 (d, *J* = 8.4 Hz, 2H), 7.25 (d, *J* = 8.4 Hz, 2H), 3.59 (s, 2H), 2.55-2.52 (m, 4H), 1.80-1.77 (m, 4H), 1.30 (s, 9H). **^{13}C NMR** (101 MHz, CD_3OD): δ 152.64, 137.39, 131.47, 127.47, 62.32, 56.12, 36.59, 33.14, 25.36. **HRMS** ($APCI^+$, m/z) calculated for $C_{15}H_{24}N$ $[M+H]^+$: 218.19033; found: 218.19014. The spectral data are identical to the previously reported.[5]

1-(Naphthalen-2-ylmethyl)pyrrolidine (3ad)

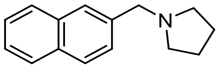

The compound was synthesised according to the **General procedure**. *DL*-Proline (57.5 mg, 0.5 mmol) affords **3ad** (96 mg, 91% using **C1**; 20 mg, 19% using **C2**). Yellow oil was obtained after column chromatography (SiO_2, EtOAc/MeOH 90:10). **^{1}H NMR** (400 MHz, $CDCl_3$): δ 7.88-7.83 (m, 4H), 7.59-7.47 (m, 3H), 3.83 (s, 2H), 2.63-2.60 (m, 4H), 1.88-1.84 (m, 4H). **^{13}C NMR** (101 MHz, $CDCl_3$): δ 139.68, 136.17, 135.44, 130.57, 130.48, 130.36, 130.08, 129.92, 128.60, 128.22, 63.60, 56.98, 26.25. **HRMS** ($APCI^+$, m/z) calculated for $C_{15}H_{18}N$ $[M+H]^+$: 212.14338; found: 212.14311. The spectral data are identical to the previously reported.[6]

1-(benzo[d][1,3]dioxol-5-ylmethyl)pyrrolidine (3ae)

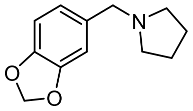

The compound was synthesised according to the **General procedure**. *DL*-Proline (57.5 mg, 0.5 mmol) affords **3ae** (97 mg, 94% using **C1**; 96 mg, 94% using **C2**). Slightly yellow oil was obtained after column chromatography (SiO_2, EtOAc/MeOH 70:30). **^{1}H NMR** (400 MHz, $CDCl_3$): δ 6.85 (d, *J* = 1.2 Hz, 1H), 6.77-6.72 (m, 2H), 5.92 (s, 2H), 3.54 (s, 2H), 2.53-2.50 (m, 4H), 1.80-1.76 (m, 4H). **^{13}C NMR** (101 MHz, $CDCl_3$): δ 150.20, 149.17, 135.42, 124.69, 112.13, 110.54, 103.49, 62.82, 56.45, 26.03. **HRMS** ($APCI^+$, m/z) calculated for $C_{12}H_{16}NO_2$ $[M+H]^+$: 206.11756; found: 206.11736. The spectral data are identical to the previously reported.[7]

1-benzylpyrrolidine (3af)

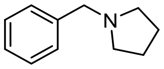

The compound was synthesised according to the **General procedure**. *DL*-Proline (57.5 mg, 0.5 mmol) affords **3af** (34 mg, 42% using **C1**; 46 mg, 58% using **C2**). Colorless solid was obtained after column chromatography (SiO_2, EtOAc/MeOH 50:50). **^{1}H NMR** (400 MHz, CD_3OD): δ 7.34-7.23 (m, 5H), 3.62 (s, 2H), 2.55-2.52 (m, 4H), 1.81-1.78 (m, 4H). **^{13}C NMR** (101 MHz, CD_3OD): δ 139.66, 131.82, 130.72, 129.95, 62.42, 56.06, 25.30. **HRMS** ($APCI^+$, m/z) calculated for $C_{11}H_{16}N$ $[M+H]^+$: 162.12773; found: 162.12732. The spectral data are identical to the previously reported.[8]

3-(pyrrolidin-1-ylmethyl)pyridine (3ag)

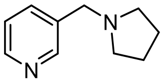

The compound was synthesised according to the **General procedure**. *DL*-Proline (57.5 mg, 0.5 mmol) affords **3ag** (31 mg, 40% using **C1**). Yellow oil was obtained after column chromatography (SiO_2, EtOAc/MeOH 90:10 to 70:30). **^{1}H NMR** (400 MHz, CD_3OD): δ 8.57 (d, *J* = 1.6 Hz, 1H), 8.51 (dd, *J* = 4.8 Hz, *J* = 1.2 Hz, 1H), 7.90 (d, *J* = 8.0 Hz, 1H), 7.47-7.44 (m, 1H), 3.91 (s, 2H), 2.81-2.77 (m, 4H), 1.91-1.88 (m, 4H). **^{13}C NMR** (101 MHz, CD_3OD): δ 152.34, 150.94, 140.73, 135.31, 126.53, 58.92, 56.08, 25.33. **HRMS** ($APCI^+$, m/z) calculated for $C_{10}H_{15}N_2$ $[M+H]^+$: 163.12298; found: 163.12287. The spectral data are identical to the previously reported.[9]

1-(thiophen-2-ylmethyl)pyrrolidine (3ah)

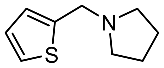

The compound was synthesised according to the **General procedure**. *DL*-Proline (57.5 mg, 0.5 mmol) affords **3ah** (28 mg, 33% using **C1**; 38 mg, 45% using **C2**). Slightly yellow oil was obtained after column chromatography (SiO_2, EtOAc/MeOH 90:10). **^{1}H NMR** (400 MHz, $CDCl_3$): δ 7.26-7.23 (m, 1H), 6.99-6.94 (m, 2H), 3.94 (s, 2H), 2.71-2.68 (m, 4H), 1.86-1.82 (m, 4H). **^{13}C NMR** (151 MHz, $CDCl_3$): δ 140.46, 126.80, 126.72, 125.43, 54.09, 53.69, 23.58. **HRMS** ($APCI^+$, m/z) calculated for $C_9H_{14}NS$ $[M+H]^+$: 168.08415; found: 168.08396.

1-(4-Fluorobenzyl)pyrrolidine (3ai)

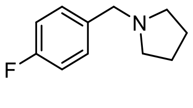

The compound was synthesised according to the **General procedure**. *DL*-Proline (57.5 mg, 0.5 mmol) affords **3ai** (67 mg, 75% using **C1**; 23 mg, 26% using **C2**). Yellow-brown oil was obtained after column chromatography (SiO_2, EtOAc/MeOH 90:10). **^{1}H NMR** (400 MHz, $CDCl_3$): δ 7.27 (dd, J = 8.4 Hz, J = 5.6 Hz, 2H), 6.97 (t, J = 8.4 Hz, 2H), 3.56 (s, 2H), 2.50-2.46 (m, 4H), 1.78-1.75 (m, 4H). **^{13}C NMR** (101 MHz, $CDCl_3$): δ 164.54 (d, $J_{C\text{-}F}$ = 246.4 Hz), 137.65 (d, $J_{C\text{-}F}$ = 3.0 Hz), 132.97 (d, $J_{C\text{-}F}$ = 8.1 Hz), 117.59 (d, $J_{C\text{-}F}$ = 21.2 Hz), 62.48, 56.69, 26.07. **HRMS** (APCI$^+$, m/z) calculated for $C_{11}H_{15}FN$ $[M+H]^+$: 180.11830; found: 180.11807.

1-(4-Chlorobenzyl)pyrrolidine (3aj)

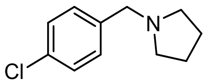

The compound was synthesised according to the **General procedure**. *DL*-Proline (57.5 mg, 0.5 mmol) affords **3aj** (88 mg, 90% using **C1**; 67 mg, 68% using **C2**). Colorless oil was obtained after column chromatography (SiO_2, EtOAc/MeOH 70:30). **^{1}H NMR** (400 MHz, $CDCl_3$): δ 7.28-7.26 (m, 4H), 3.58 (s, 2H), 2.51-2.48 (m, 4H), 1.80-1.77 (m, 4H). **^{13}C NMR** (101 MHz, $CDCl_3$): δ 140.32, 135.27, 132.83, 131.00, 62.52, 56.73, 26.09. **HRMS** (APCI$^+$, m/z) calculated for $C_{11}H_{15}ClN$ $[M+H]^+$: 196.08875; found: 196.08835. The spectral data are identical to the previously reported.[10]

1-(4-(Trifluoromethyl)benzyl)pyrrolidine (3ak)

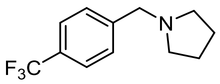

The compound was synthesised according to the **General procedure**. *DL*-Proline (57.5 mg, 0.5 mmol) affords **3ak** (96 mg, 84% using **C1**; 42 mg, 37% using **C2**). Orange-brown oil was obtained after column chromatography (SiO_2, EtOAc/MeOH 90:10). **^{1}H NMR** (400 MHz, $CDCl_3$): δ 7.56 (d, J = 8.0 Hz, 2H), 7.45 (d, J = 8.0 Hz, 2H), 3.66 (s, 2H), 2.52-2.49 (m, 4H), 1.80-1.77 (m, 4H). **^{13}C NMR** (151 MHz, $CDCl_3$): δ 142.59, 128.14 (q, $J_{C\text{-}F}$ = 32.2 Hz), 127.94, 124.12 (q, $J_{C\text{-}F}$ = 3.8 Hz) , 123.30 (q, $J_{C\text{-}F}$ = 272.0 Hz), 59.13, 53.17, 22.47. **HRMS** (APCI$^+$, m/z) calculated for $C_{12}H_{15}F_3N$ $[M+H]^+$: 230.11511; found: 230.11517.

4-(pyrrolidin-1-ylmethyl)benzonitrile (3al)

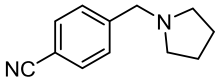

The compound was synthesised according to the **General procedure**. *DL*-Proline (57.5 mg, 0.5 mmol) affords **3al** (77 mg, 83% using **C1**, 17 mg 18% using **C2**). Yellow oil was obtained after column chromatography (SiO_2, EtOAc/MeOH 90:10). **^{1}H NMR** (400 MHz, $CDCl_3$): δ 7.52 (d, J = 8.0 Hz, 2H), 7.39 (d, J = 8.4 Hz, 2H), 3.60 (s, 2H), 2.45-2.42 (m, 4H), 1.74-1.70 (m, 4H). **^{13}C NMR** (151 MHz, $CDCl_3$): δ 145.25, 132.00, 129.26, 118.95, 110.57, 60.10, 54.16, 23.51. **HRMS** (APCI$^+$, m/z) calculated for $C_{12}H_{15}N_2$ $[M+H]^+$: 187.12298; found: 187.12288.

Methyl 4-(pyrrolidin-1-ylmethyl)benzoate (3am)

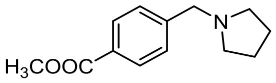

The compound was synthesised according to the **General procedure**. *DL*-Proline (57.5 mg, 0.5 mmol) affords **3am** (71 mg, 65% using **C1**, 15 mg 14% using **C2**). Orange-brown oil was obtained after column chromatography (SiO_2, EtOAc/MeOH 90:10). **^{1}H NMR** (400 MHz, $CDCl_3$): δ 7.95 (d,

J = 8.0 Hz, 2H), 7.37 (d, J = 8.0 Hz, 2H), 3.87 (s, 3H), 3.63 (s, 2H), 2.49-2.46 (m, 4H), 1.77-1.74 (m, 4H). **^{13}C NMR** (151 MHz, $CDCl_3$): δ 167.02, 144.81, 129.56, 128.66, 60.34, 54.20, 51.96, 23.48. **HRMS** ($APCI^+$, m/z) calculated for $C_{13}H_{18}NO_2$ $[M+H]^+$: 220.13321; found: 220.13315. The spectral data are identical to the previously reported.[11]

1-(4-Nitrobenzyl)pyrrolidine (3an)

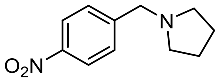

The compound was synthesised according to the **General procedure**. *DL*-Proline (57.5 mg, 0.5 mmol) affords **3an** (102 mg, 99% using **C1**). Orange oil was obtained after column chromatography (SiO_2, EtOAc/MeOH 90:10). **^{1}H NMR** (400 MHz, CD_3OD): δ 8.17 (d, J = 8.8 Hz, 2H), 7.57 (d, J = 8.8 Hz, 2H), 3.74 (s, 2H), 2.57-2.53 (m, 4H), 1.82-1.80 (m, 4H). **^{13}C NMR** (101 MHz, CD_3OD): δ 149.83, 148.90, 132.34, 125.68, 61.85, 56.29, 25.52. **HRMS** ($APCI^+$, m/z) calculated for $C_{11}H_{15}N_2O_2$ $[M+H]^+$: 207.11280; found: 207.11284.

1-(4-Methoxybenzyl)piperidine (3ba)

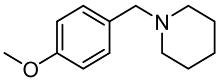

The compound was synthesised according to the **General procedure**. Piperidine-2-carboxylic acid (64.5 mg, 0.5 mmol) affords **3ba** (55 mg, 54% using **C2**). Colourless oil was obtained after column chromatography (SiO_2, EtOAc/MeOH 70:30). **^{1}H NMR** (400 MHz, $CDCl_3$): δ 7.22 (d, J = 8.4 Hz, 2H), 6.85 (d, J = 8.8 Hz, 2H), 3.80 (s, 3H), 3.42 (s, 2H), 2.39-2.30 (m, 4H), 1.59-1.54 (m, 4H), 1.45-1.39 (m, 2H). **^{13}C NMR** (101 MHz, $CDCl_3$): δ 161.23, 133.08, 116.10, 65.83, 57.88, 56.96, 28.57, 27.04. **HRMS** ($APCI^+$, m/z) calculated for $C_{13}H_{20}NO$ $[M+H]^+$: 206.15394; found: 206.15375.

1-(4-Nitrobenzyl)piperidine (3bn)

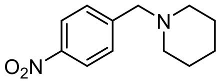

The compound was synthesised according to the **General procedure**. Piperidine-2-carboxylic acid (64.5 mg, 0.5 mmol) affords **3bn** (71 mg, 64% using **C1**). Colourless oil was obtained after column chromatography (SiO_2, EtOAc/MeOH 90:10). **^{1}H NMR** (400 MHz, CD_3OD): δ 8.19 (d, J = 8.8 Hz, 2H), 7.58 (d, J = 8.8 Hz, 2H), 3.60 (s, 2H), 2.46-2.36 (*br* m, 4H), 1.63-1.57 (m, 4H), 1.49-1.44 (m, 2H). **^{13}C NMR** (151 MHz, CD_3OD): δ 148.64, 147.12, 131.35, 124.30, 63.73, 55.52, 26.70, 25.12. **HRMS** ($APCI^+$, m/z) calculated for $C_{12}H_{17}N_2O_2$ $[M+H]^+$: 221.12845; found: 221.12820.

1-Cyclohexylpyrrolidine (3aq)

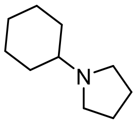

The compound was synthesised according to the **General procedure**. *DL*-Proline (57.5 mg, 0.5 mmol) affords **3aq** (43 mg, 57% using **C1**). Yellow oil was obtained after column chromatography (SiO_2, EtOAc/MeOH 70:30 to 50:50). **^{1}H NMR** (400 MHz, CD_3OD): δ 2.75-2.72 (m, 4H), 2.24-2.18 (m, 1H), 2.04-2.01 (m, 2H), 1.85-1.77 (m, 6H), 1.67-1.64 (m, 1H), 1.35-1.15 (m, 5H). **^{13}C NMR** (101 MHz, CD_3OD): δ 66.69, 53.65, 33.66, 28.13, 27.45, 25.22. **HRMS** ($APCI^+$, m/z) calculated for

$C_{10}H_{20}N$ $[M+H]^+$: 154.15903; found: 154.15897. The spectral data are identical to the previously reported.[8]

1-(1,2,3,4-tetrahydronaphthalen-1-yl)pyrrolidine (3at)

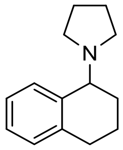

The compound was synthesised according to the **General procedure**. *DL*-Proline (57.5 mg, 0.5 mmol) affords **3at** (50 mg, 49% using **C1**). White solid was obtained after column chromatography (SiO_2, EtOAc/MeOH 70:30). **^{1}H NMR** (400 MHz, CD_3OD): δ 7.27-7.24 (m, 1H), 7.15-7.11 (m, 1H), 7.08-7.05 (m, 2H), 3.44-3.42 (m, 1H), 2.94-2.87 (m, 1H), 2.79-2.66 (m, 3H), 2.54-2.50 (m, 2H), 2.14-2.03 (m, 2H), 1.79-1.67 (m, 6H). **^{13}C NMR** (101 MHz, CD_3OD): δ 140.07, 139.94, 132.20, 131.53, 129.46, 127.10, 64.27, 53.53, 30.81, 28.28, 25.43, 20.64. **HRMS** ($APCI^+$, m/z) calculated for $C_{14}H_{20}N$ $[M+H]^+$: 202.15903; found: 202.15890.

1-(1-phenylethyl)pyrrolidine (3au)

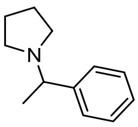

The compound was synthesised according to the **General procedure**. *DL*-Proline (57.5 mg, 0.5 mmol) affords **3au** (109 mg, 62% using **C1**; 98 mg, 56% using **C2**). White solid was obtained after column chromatography (SiO_2, EtOAc/MeOH 90:10). **^{1}H NMR** (400 MHz, CD_3OD): δ 7.36-7.22 (m, 5H), 3.35-3.33 (m, 1H), 2.70-2.64 (m, 2H), 2.46-2.42 (m, 2H), 1.84-1.77 (m, 4H), 1.44 (d, J = 6.8 Hz, 3H). **^{13}C NMR** (101 MHz, CD_3OD): δ 130.79, 129.73, 129.65, 68.76, 55.29, 25.37, 23.84. **HRMS** ($APCI^+$, m/z) calculated for $C_{12}H_{18}N$ $[M+H]^+$: 176.14338; found: 176.14337. The spectral data are identical to the previously reported.[8]

1-(3-phenylpropyl)pyrrolidine (3aw)

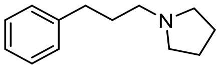

The compound was synthesised according to the **General procedure**. *DL*-Proline (57.5 mg, 0.5 mmol) affords **3aw** (77 mg, 81% using **C1**). Yellow oil was obtained after column chromatography (SiO_2, EtOAc/MeOH 70:30). **^{1}H NMR** (400 MHz, CD_3OD): δ 7.27-7.23 (m, 2H), 7.19-7.13 (m, 3H), 2.63 (t, J = 7.6 Hz, 2H), 2.56-2.48 (m, 6H), 1.90-1.78 (m, 6H). **^{13}C NMR** (101 MHz, CD_3OD): δ 144.34, 130.65, 130.64, 128.17, 58.27, 56.23, 36.05, 32.63, 25.40. **HRMS** ($APCI^+$, m/z) calculated for $C_{13}H_{20}N$ $[M+H]^+$: 190.15903; found: 190.15911. The spectral data are identical to the previously reported.[12]

1-Nonylpyrrolidine (3ay)

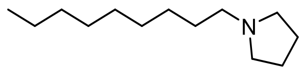

The compound was synthesised according to the **General procedure**. *DL*-Proline (57.5 mg, 0.5 mmol) affords **3ay** (53 mg, 54% using **C1**; 72 mg, 73% using **C2**). Colorless oil was obtained after column chromatography (SiO_2, EtOAc/MeOH 70:30). **^{1}H NMR** (400 MHz, $CDCl_3$): δ 2.52-2.49 (m, 4H), 2.44-2.40 (m, 2H), 1.79-1.76 (m, 4H), 1.54-1.47 (m, 2H), 1.29-1.25 (m,12H), 0.87 (t, J = 6.4

Hz, 3H). **^{13}C NMR** (101 MHz, $CDCl_3$): δ 56.71, 54.20, 31.89, 29.61, 29.55, 29.29, 29.00, 27.74, 23.40, 22.67, 14.10. **HRMS** (APCI$^+$, m/z) calculated for $C_{13}H_{28}N$ $[M+H]^+$: 198.22163; found: 198.22139.

1-Nonylpiperidine (3by)

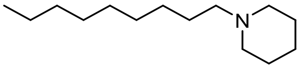

The compound was synthesised according to the **General procedure**. Piperidine-2-carboxylic acid (64.5 mg, 0.5 mmol) affords **3by** (58 mg, 55% using **C1**; 65 mg, 62% using **C2**). Colorless oil was obtained after column chromatography (SiO_2, EtOAc/MeOH 70:30). **^{1}H NMR** (400 MHz, $CDCl_3$): δ 2.42-2.30 (m, 4H), 2.27-2.23 (m, 2H), 1.60-1.54 (m, 4H), 1.50-1.41 (m, 4H), 1.29-1.18 (m,12H), 0.86 (t, *J* = 6.4 Hz, 3H). **^{13}C NMR** (101 MHz, $CDCl_3$): δ 62.28, 57.23, 34.52, 32.25, 32.19, 31.92, 30.41, 29.51, 28.55, 27.11, 25.30, 16.73. **HRMS** (APCI$^+$, m/z) calculated for $C_{14}H_{30}N$ $[M+H]^+$: 212.23728; found: 212.23742. The spectral data are identical to the previously reported.[13]

1-((*5S,8R,9S,10S,13R,14S,17R*)-10,13-dimethyl-17-((*S*)-6-methylheptan-2-yl)hexadecahydro-*1H*-cyclopenta[a]phenanthren-3-yl)pyrrolidine (3az)

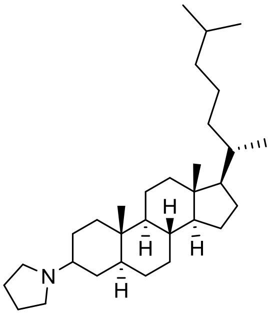

The compound was synthesised according to the **General procedure**. *DL*-Proline (57.5 mg, 0.5 mmol) affords **3az** (110 mg, 50% using **C1**). White solid was obtained after column chromatography (SiO_2, EtOAc/MeOH 70:30 to 50:50). **^{1}H NMR** (400 MHz, $CDCl_3$): δ 2.59-2.53 (m, 4H), 1.98-1.91 (m, 2H), 1.83-0.78 (m, 45H), 0.64-0.58 (m, 4H). **^{13}C NMR** (151 MHz, $CDCl_3$): δ 64.45, 56.57, 56.31, 54.49, 51.73, 45.32, 42.62, 40.09, 39.53, 37.48, 36.19, 35.80, 35.75, 35.53, 32.17, 28.98, 28.26, 28.02, 24.21, 23.84, 23.21, 22.82, 22.57, 21.11, 18.68, 12.34, 12.08. **HRMS** (APCI$^+$, m/z) calculated for $C_{31}H_{56}N$ $[M+H]^+$: 442.44073; found: 442.44132.

1-benzylpyrrolidine-2-carbonitrile (5a)

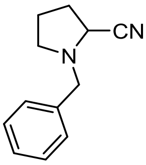

The compound was synthesised according to the **General procedure**. Mandelonitrile (133 mg, 1 mmol) affords **5a** (132 mg, 71% using **C1**; 132 mg, 71% using **C2**; 7:1 mixture of regioisomers). Colorless liquid was obtained after column chromatography (SiO_2, EtOAc/pentane 5:95). Characterisation data of the major regioisomer: **^{1}H NMR** (400 MHz, $CDCl_3$): δ 7.38-7.26 (m, 5H), 3.93 (d, *J* = 12.8 Hz, 1H), 3.71-3.66 (m, 2H), 2.97-2.91 (m, 1H), 2.59 (q, *J* = 8.0 Hz, 1H), 2.22-2.08 (m, 2H), 2.01-1.85 (m, 2H). **^{13}C NMR** (101 MHz, $CDCl_3$): δ 140.27, 131.50, 131.16, 130.15, 120.64, 59.19, 55.87, 53.87, 32.20, 24.56. **HRMS** (APCI$^+$, m/z) calculated for $C_{12}H_{15}N_2$ $[M+H]^+$: 187.12298; found: 187.12280. The spectral data are identical to the previously reported.[14]

1-(4-methylbenzyl)pyrrolidine-2-carbonitrile (5b)

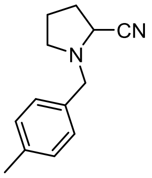

The compound was synthesised according to the **General procedure**. 4-Methylmandelonitrile (147 mg, 1 mmol) affords **5b** (148 mg, 74% using **C1**; 144 mg, 72% using **C2**; 3:1 mixture of regioisomers). Colorless liquid was obtained after column chromatography (SiO_2, EtOAc/pentane 5:95). Characterisation data of the major regioisomer: **^{1}H NMR** (400 MHz, $CDCl_3$): δ 7.26 (d, *J* = 8.0 Hz, 2H), 7.15 (d, *J* = 7.6 Hz, 2H), 3.89 (d, *J* = 13.2 Hz, 1H), 3.70-3.68 (m, 1H), 3.64 (d, *J* = 12.8 Hz, 1H), 2.97-2.91 (m, 1H), 2.58 (q, *J* = 8.4 Hz, 1H), 2.35 (s, 3H), 2.21-2.07 (m, 2H), 2.00-1.86 (m, 2H). **^{13}C NMR** (101 MHz, $CDCl_3$): δ 139.77, 137.21, 131.83, 131.47, 120.66, 58.88, 55.79, 53.82, 32.18, 24.54, 23.79. **HRMS** (APCI$^+$, m/z) calculated for $C_{13}H_{17}N_2$ $[M+H]^+$: 201.13863; found: 201.13835. The spectral data are identical to the previously reported.[15]

1-(4-chlorobenzyl)pyrrolidine-2-carbonitrile (5c)

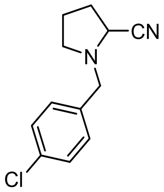

The compound was synthesised according to the **General procedure**. 4-chloromandelonitrile (167.6 mg, 1 mmol) affords **5c** (164 mg, 75% using **C1**; 168 mg, 76% using **C2**; 11:1 mixture of regioisomers). Colorless liquid was obtained after column chromatography (SiO_2, EtOAc/pentane 5:95). Characterisation data of the major regioisomer: **^{1}H NMR** (400 MHz, $CDCl_3$): δ 7.29 (m, 4H), 3.86 (d, *J* = 13.2 Hz, 1H), 3.68-3.66 (m, 1H), 3.63 (d, *J* = 13.2 Hz, 1H), 2.93-2.87 (m, 1H), 2.56 (q, *J* = 8.0 Hz, 1H), 2.21-2.07 (m, 2H), 2.00-1.85 (m, 2H). **^{13}C NMR** (101 MHz, $CDCl_3$): δ 138.83, 135.87, 132.78, 131.29, 120.48, 58.46, 55.84, 53.84, 32.18, 24.59. **HRMS** (APCI$^+$, m/z) calculated for $C_{12}H_{14}ClN_2$ $[M+H]^+$: 221.08400; found: 221.08370. The spectral data are identical to the previously reported.[15]

^{1}H and ^{13}C NMR spectra

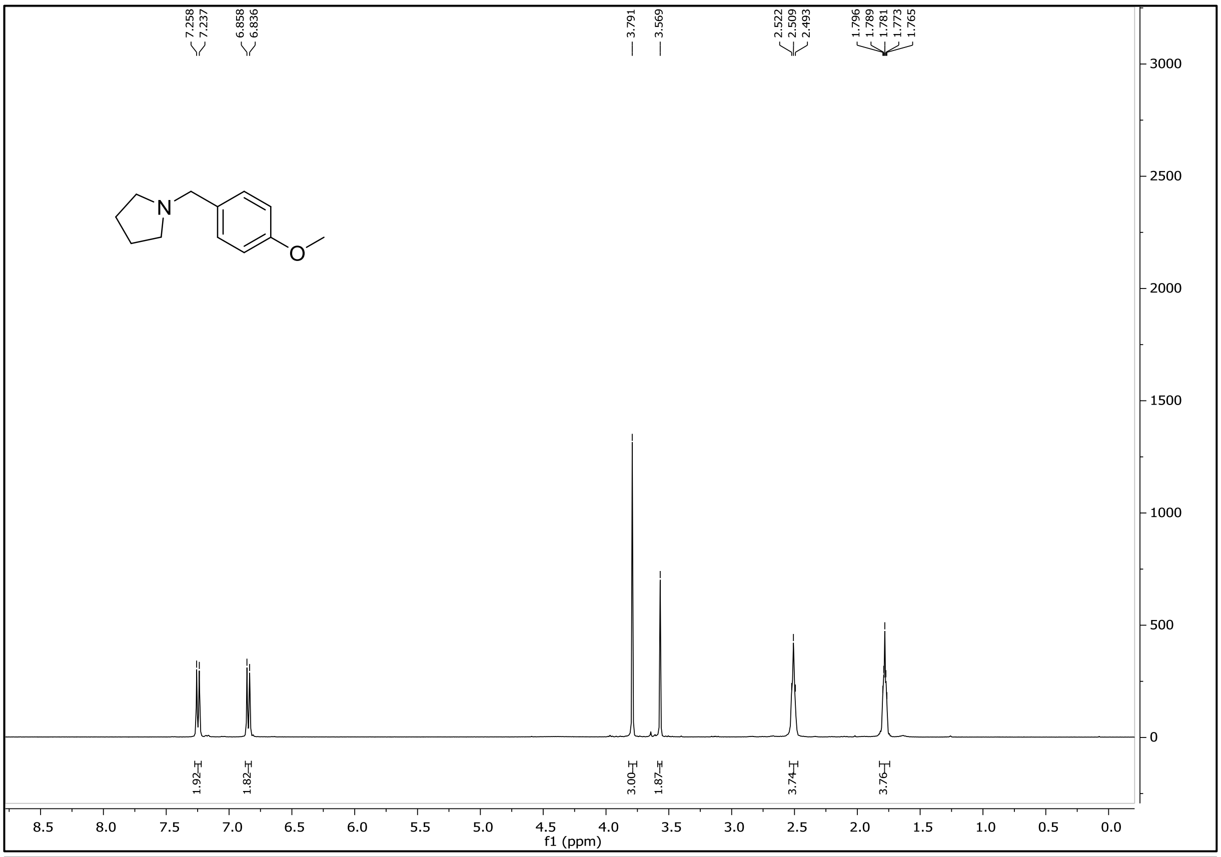

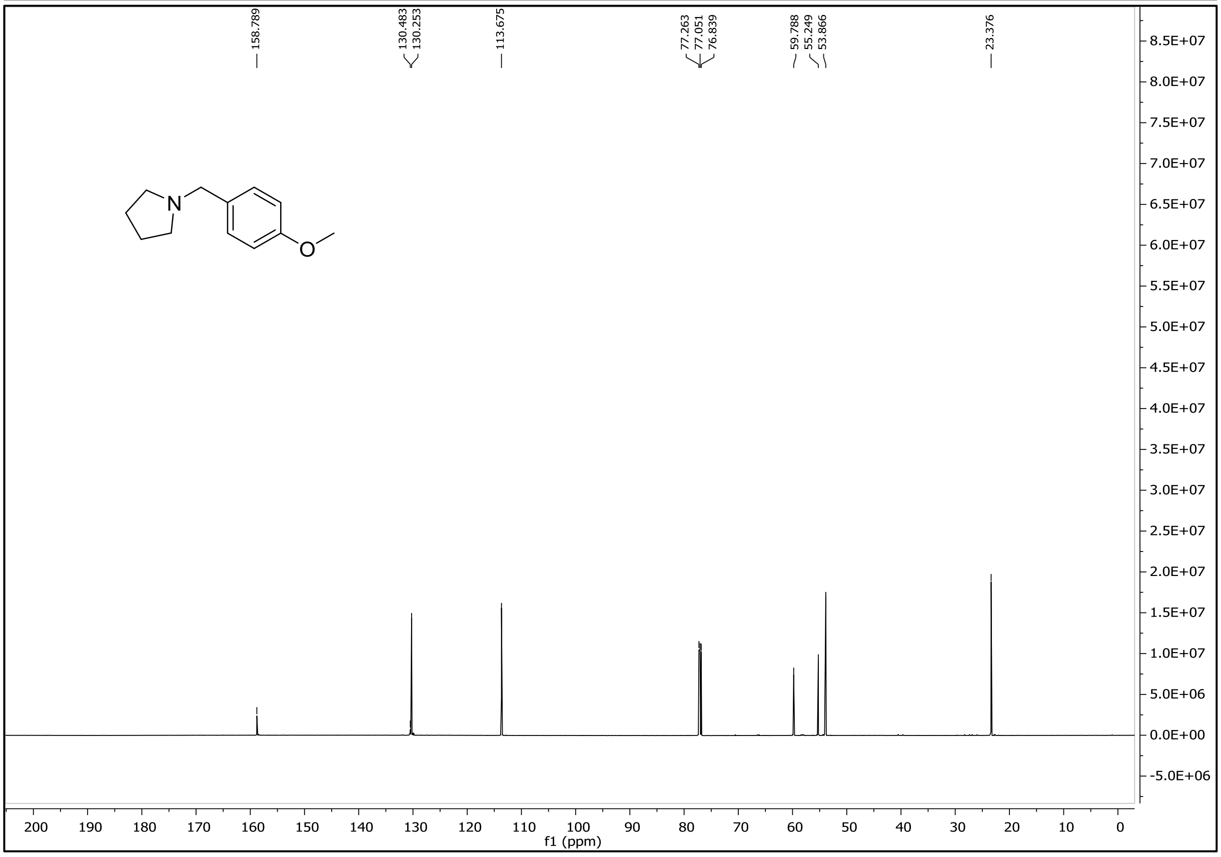

Supplementary Figure 8: ^{1}H and ^{13}C NMR of compound **3aa.**

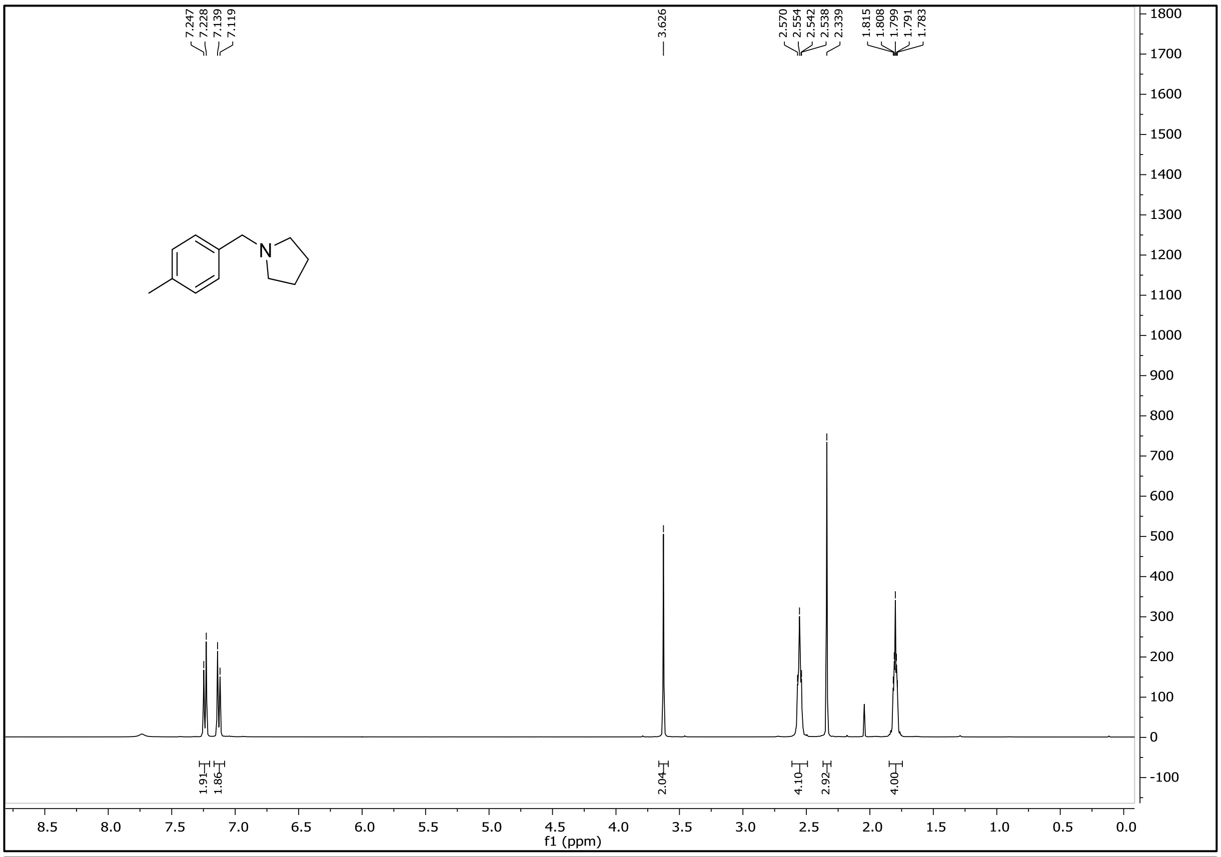

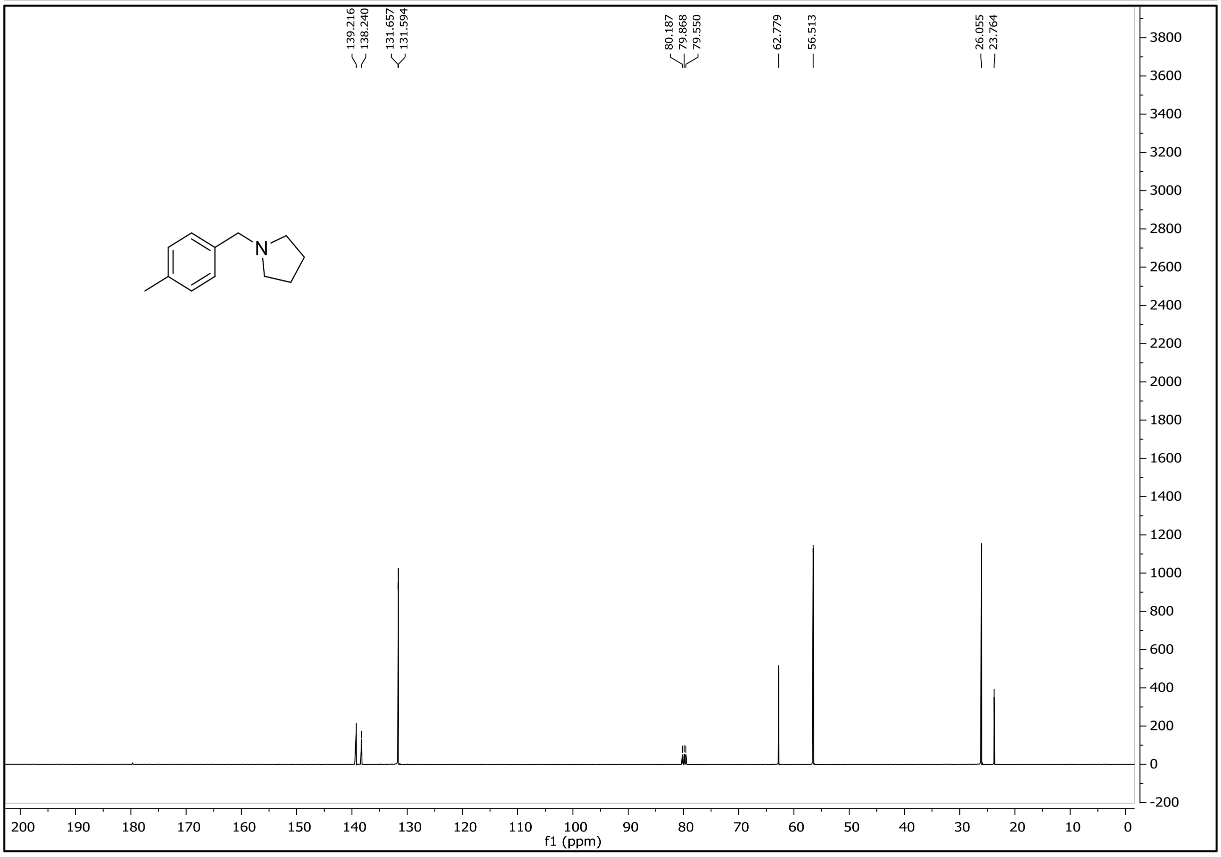

Supplementary Figure 9: ^{1}H and ^{13}C NMR of compound **3ab.**

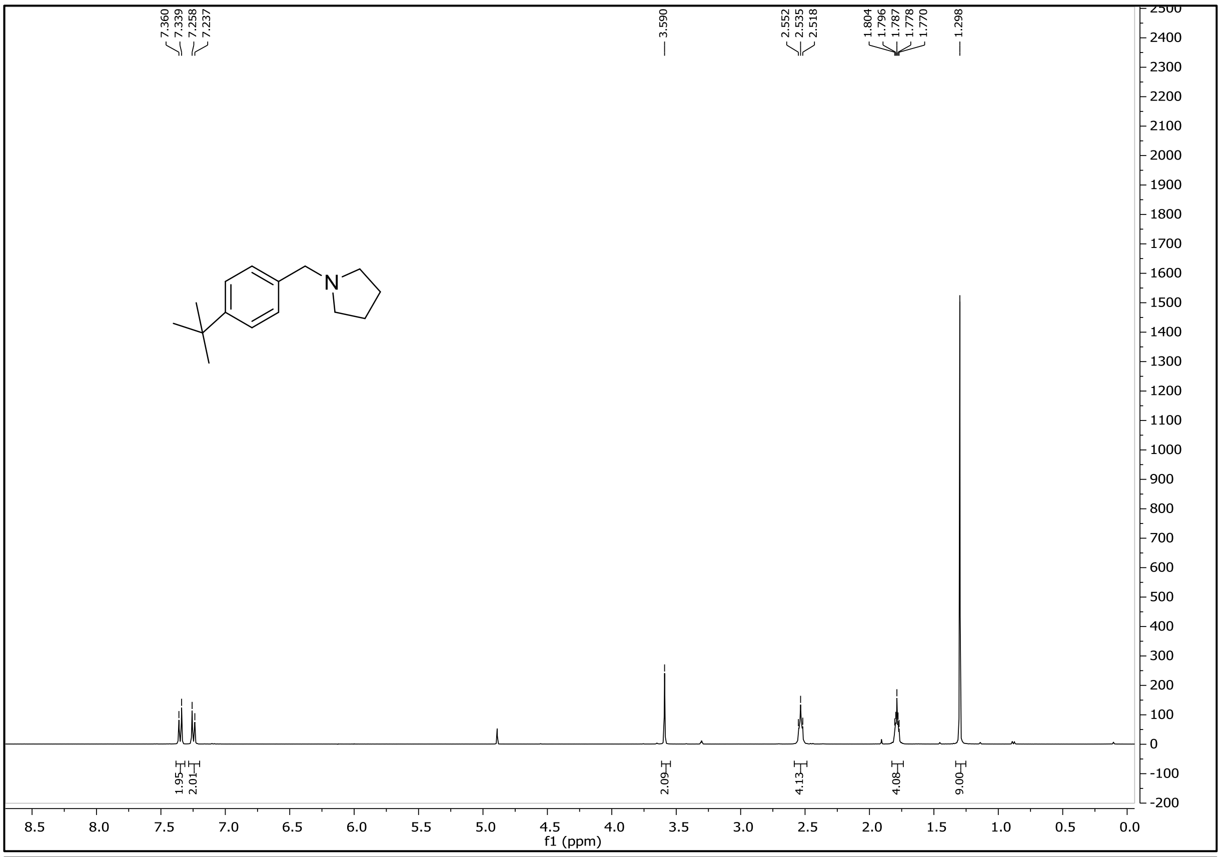

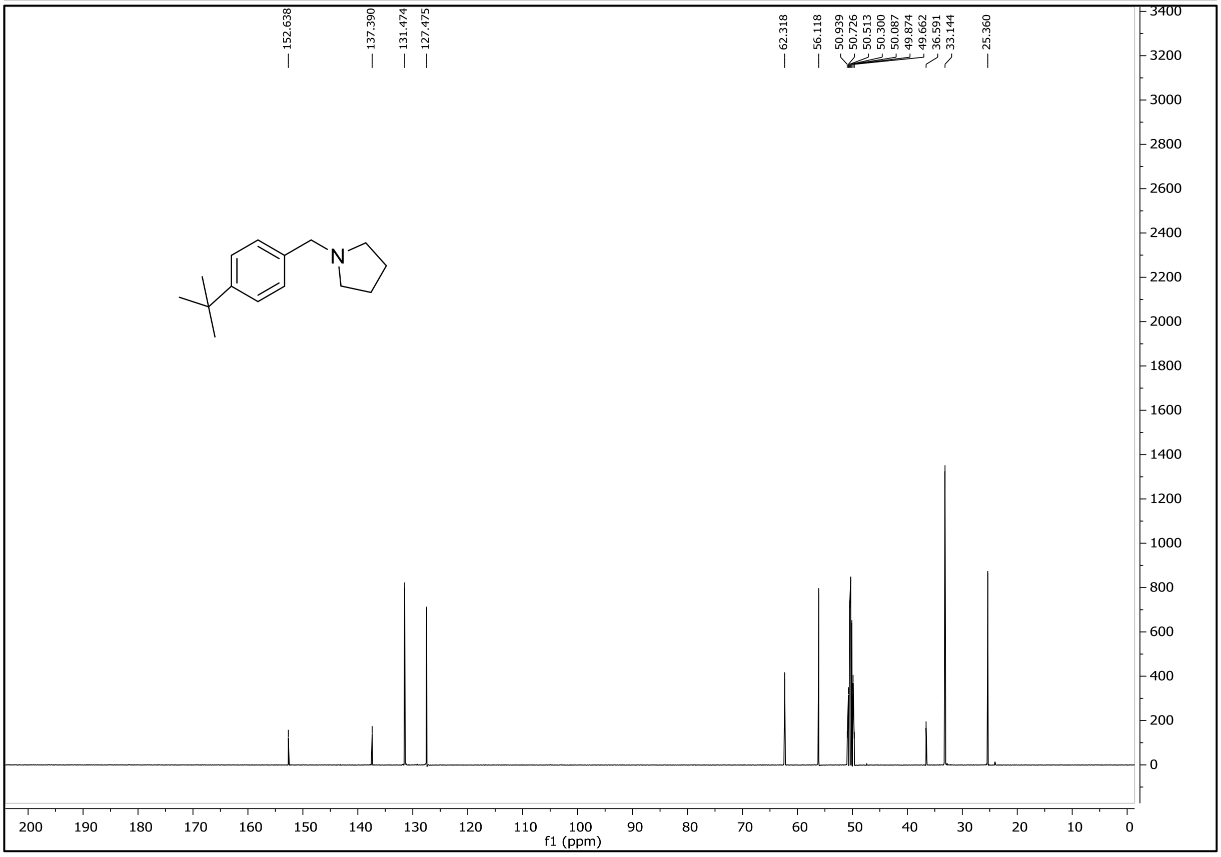

Supplementary Figure 10: ^{1}H and ^{13}C NMR of compound **3ac.**

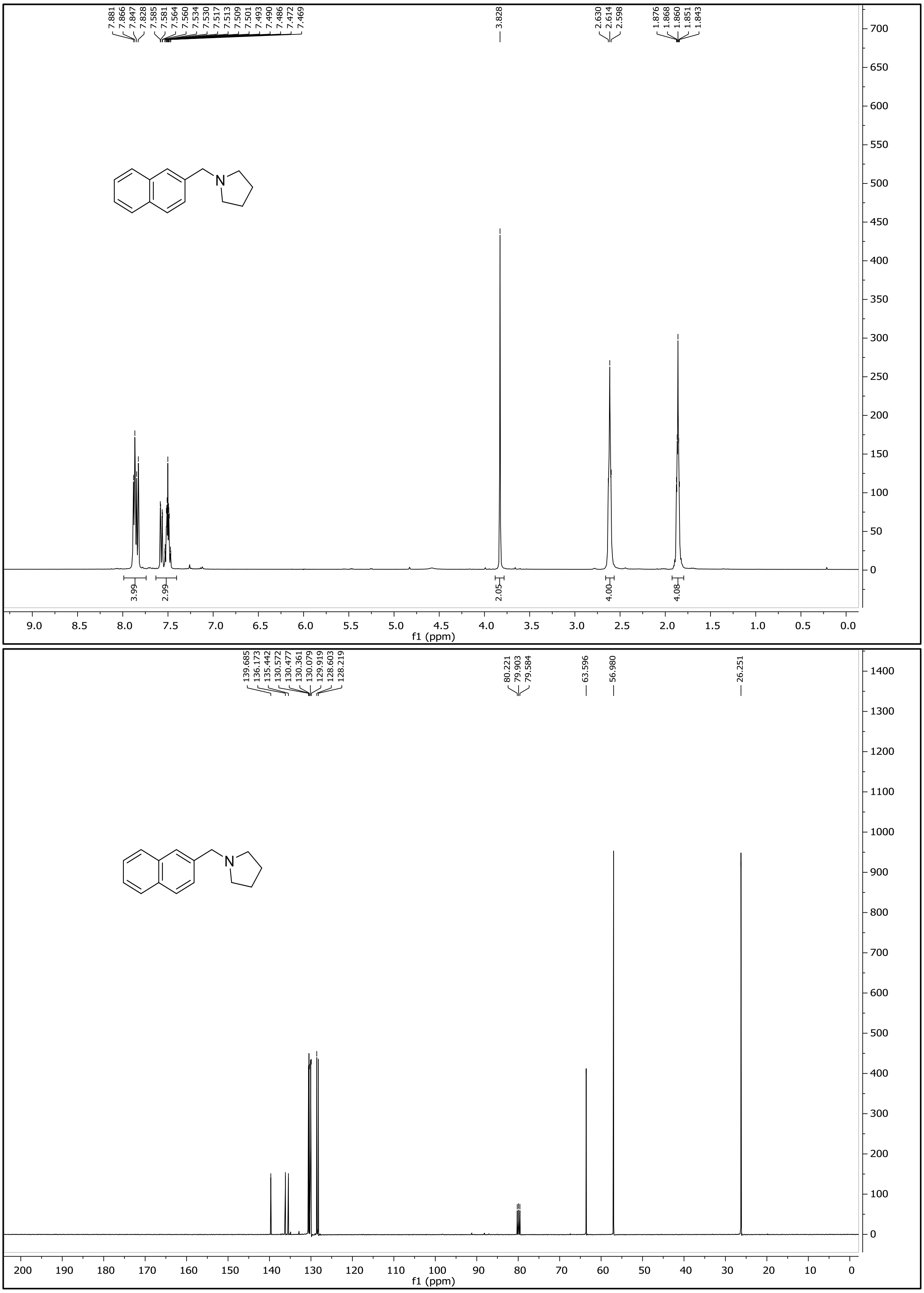

Supplementary Figure 11: ^{1}H and ^{13}C NMR of compound **3ad**.

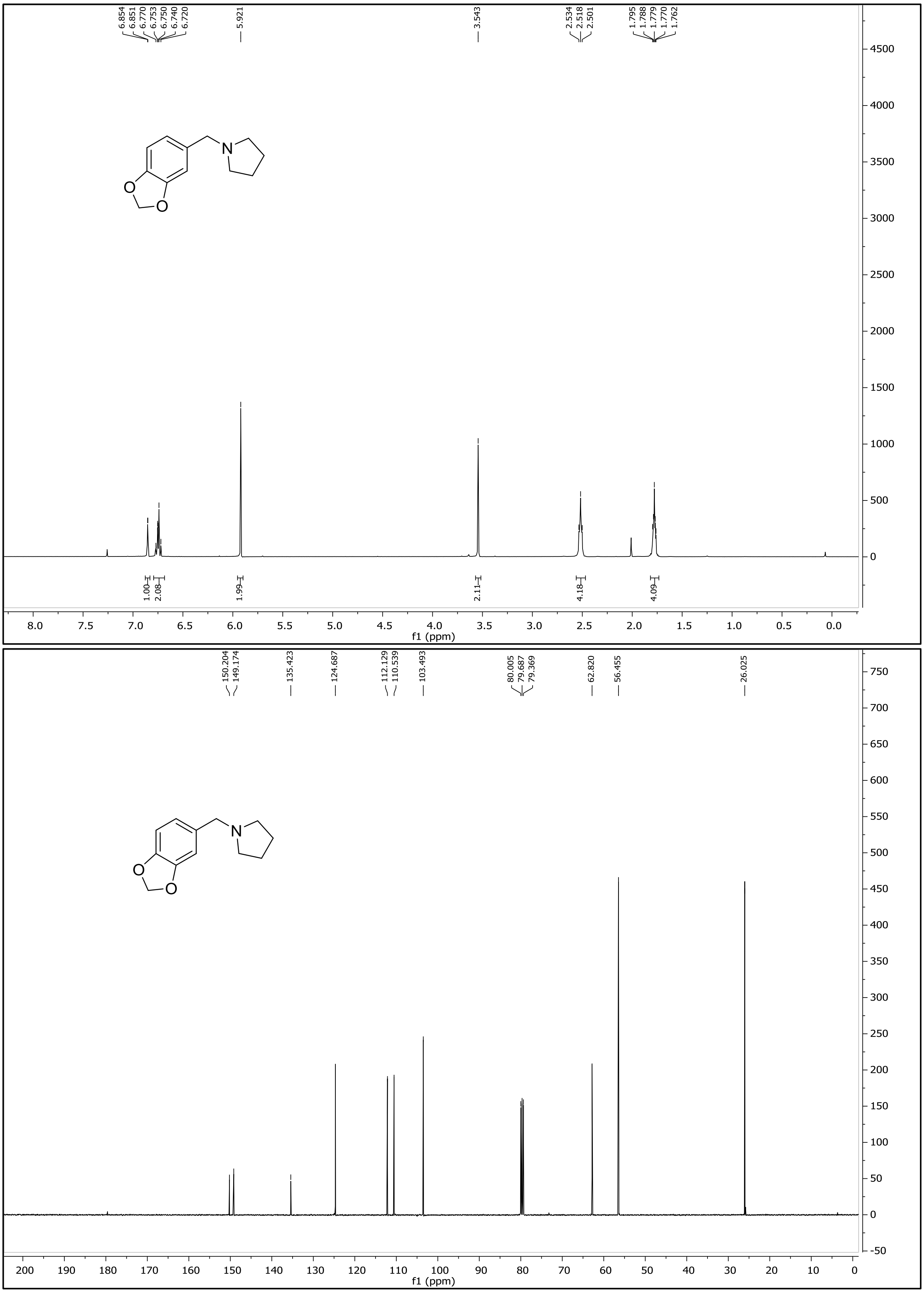

Supplementary Figure 12: ^{1}H and ^{13}C NMR of compound **3ae.**

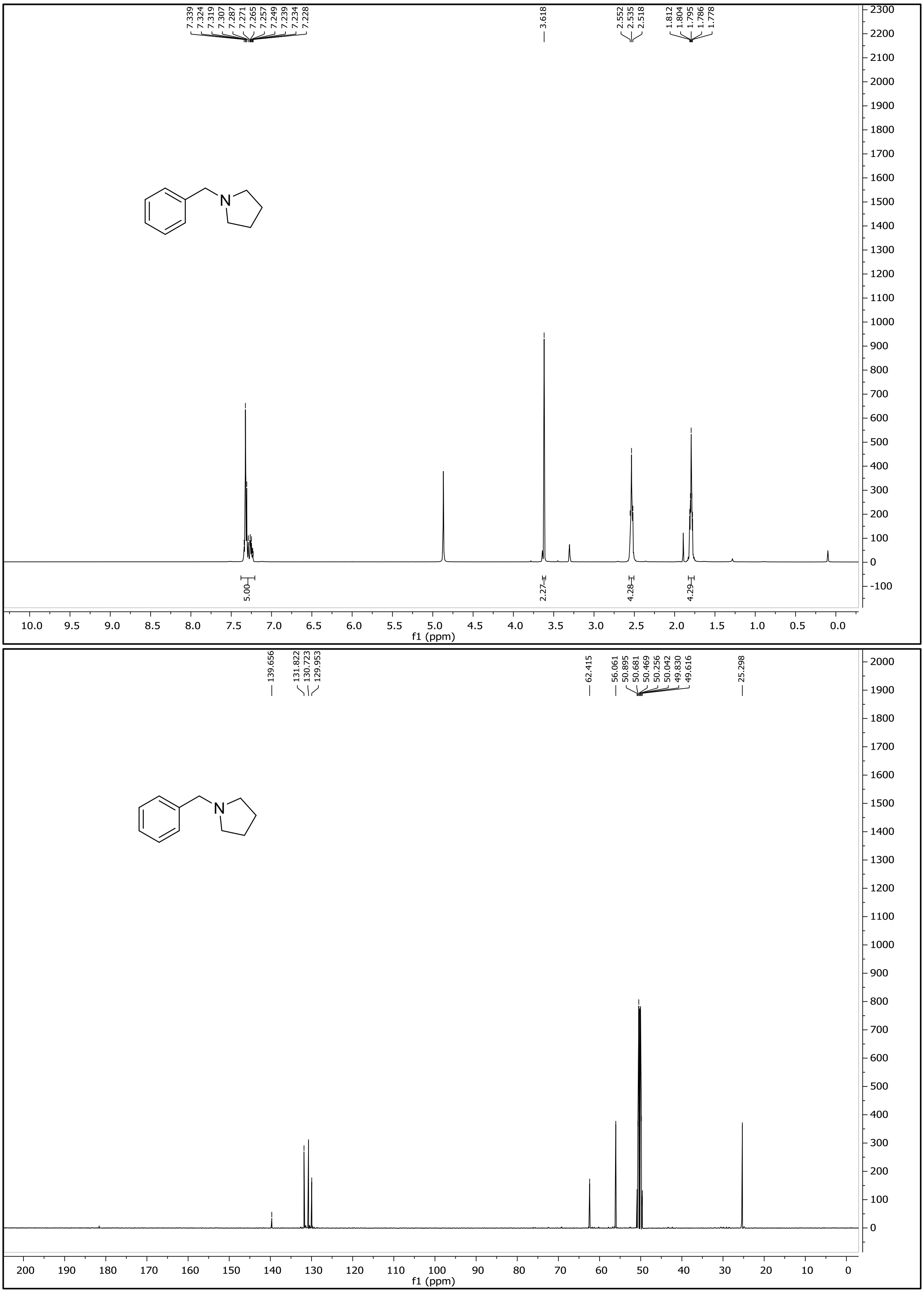

Supplementary Figure 13: ^{1}H and ^{13}C NMR of compound **3af.**

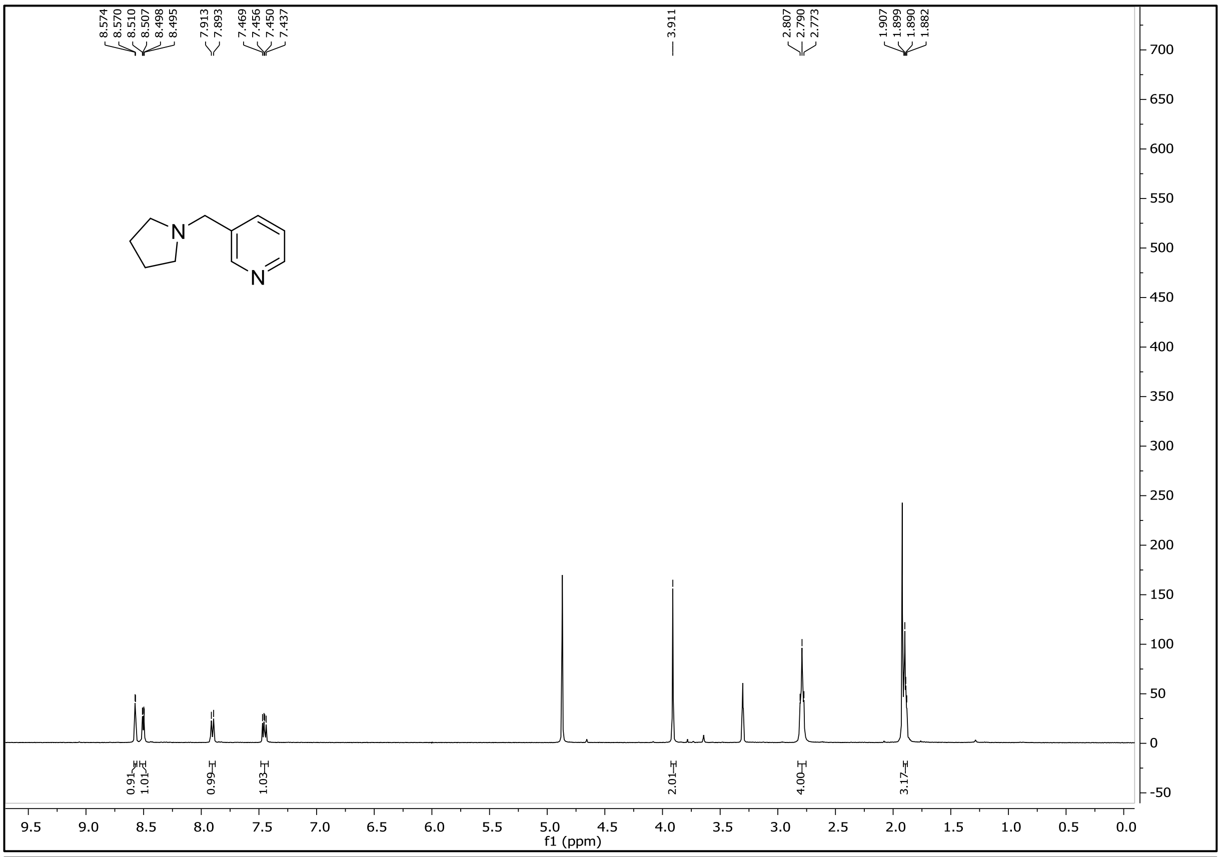

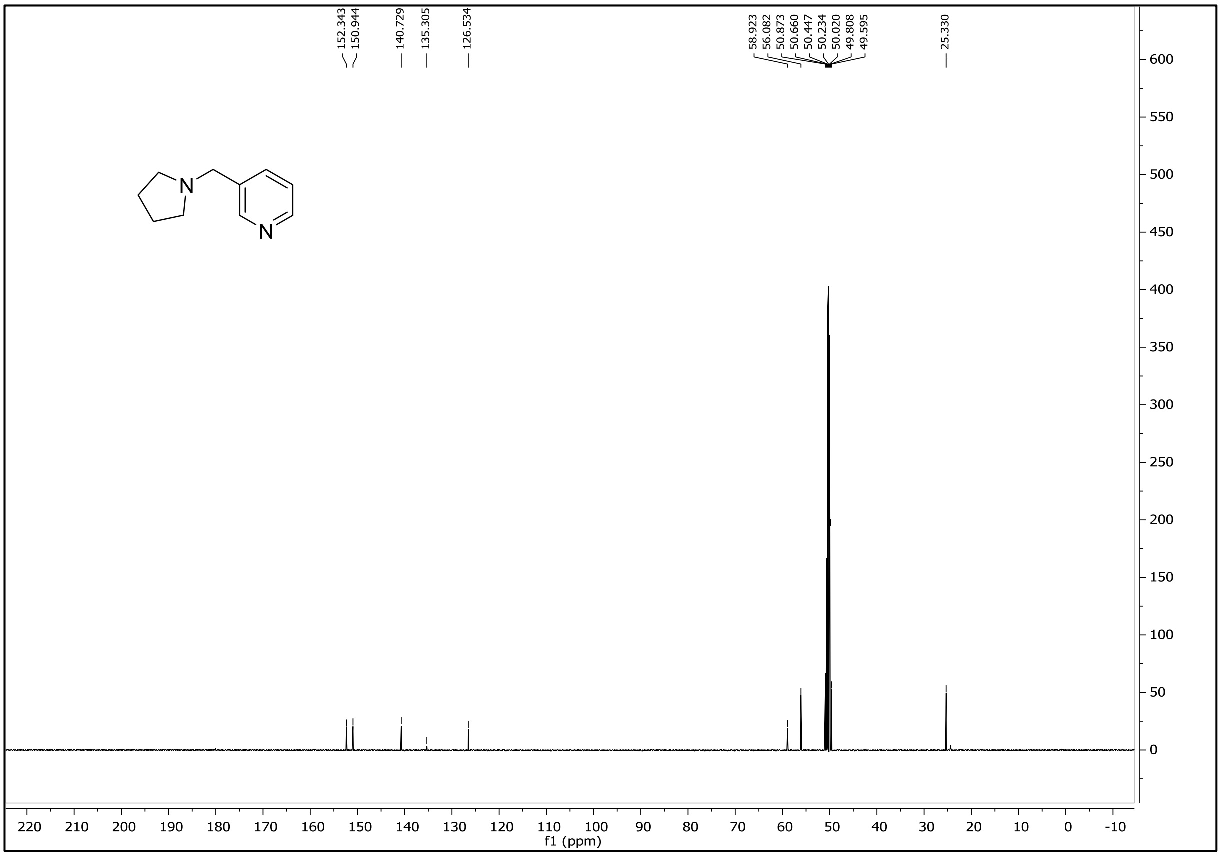

Supplementary Figure 14: ^{1}H and ^{13}C NMR of compound **3ag.**

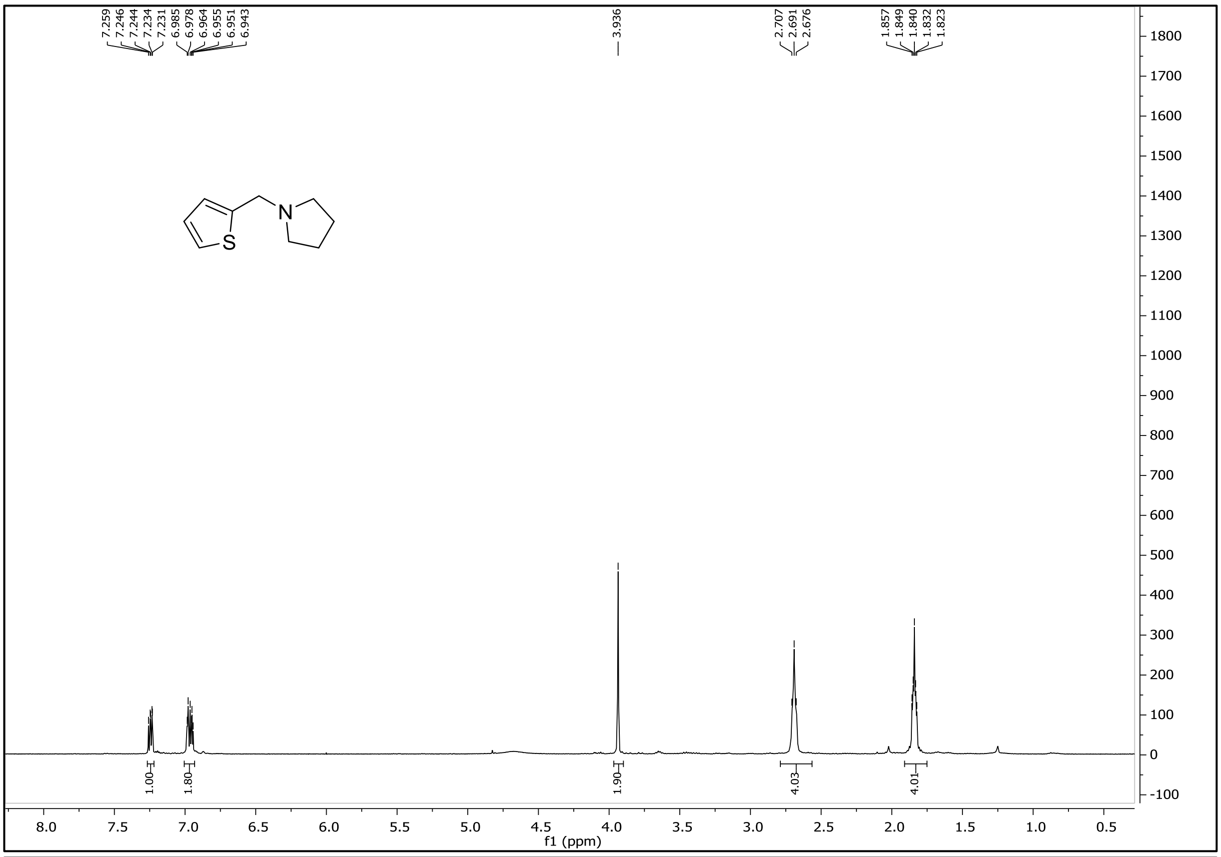

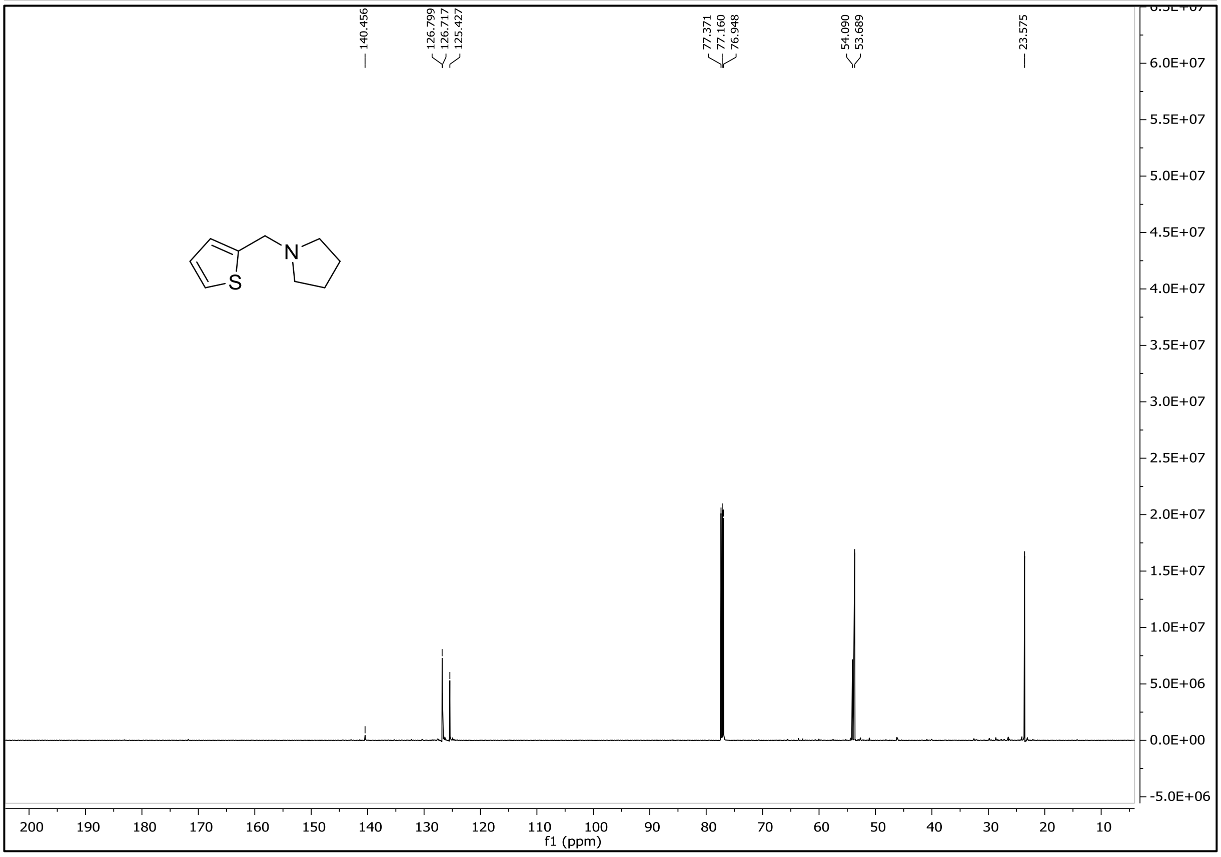

Supplementary Figure 15: ^{1}H and ^{13}C NMR of compound **3ah.**

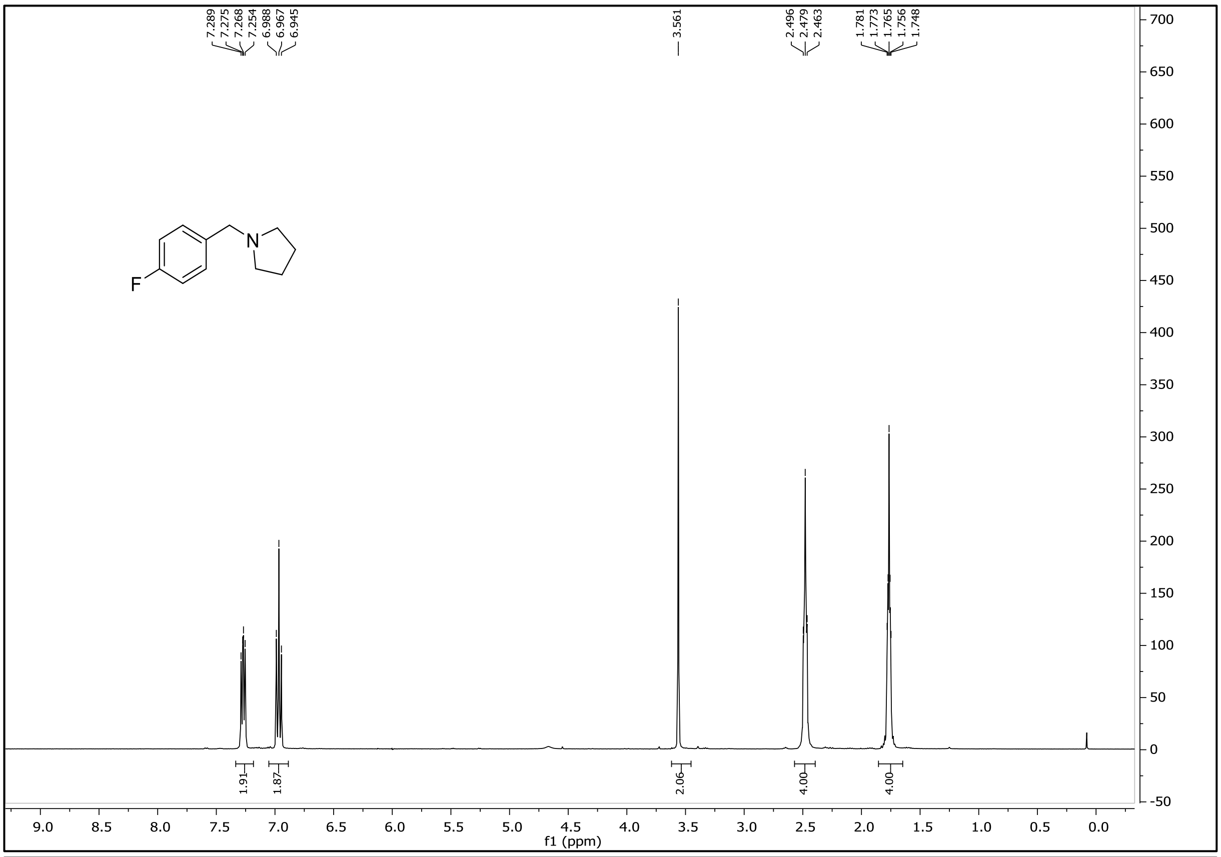

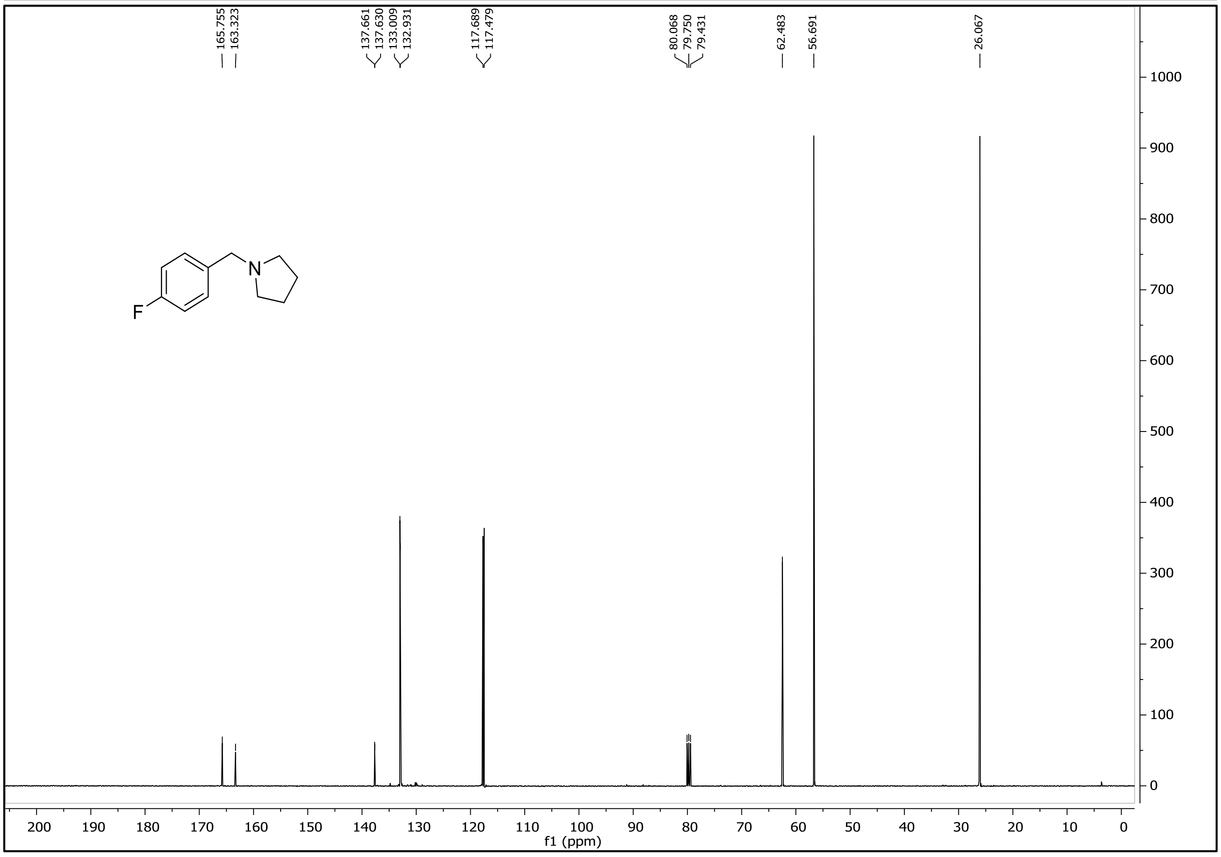

Supplementary Figure 16: ^{1}H and ^{13}C NMR of compound **3ai**.

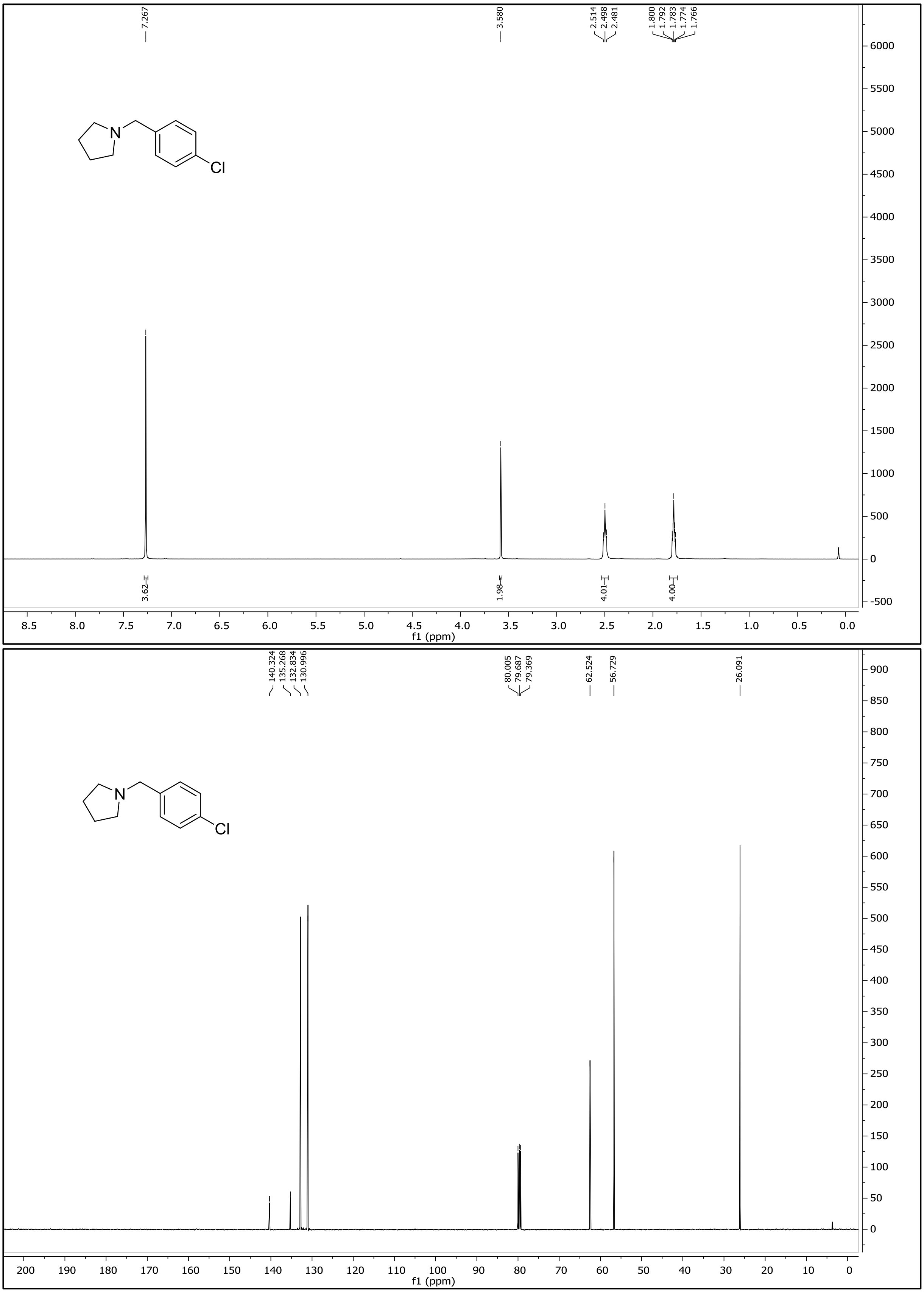

Supplementary Figure 17: ^{1}H and ^{13}C NMR of compound **3aj.**

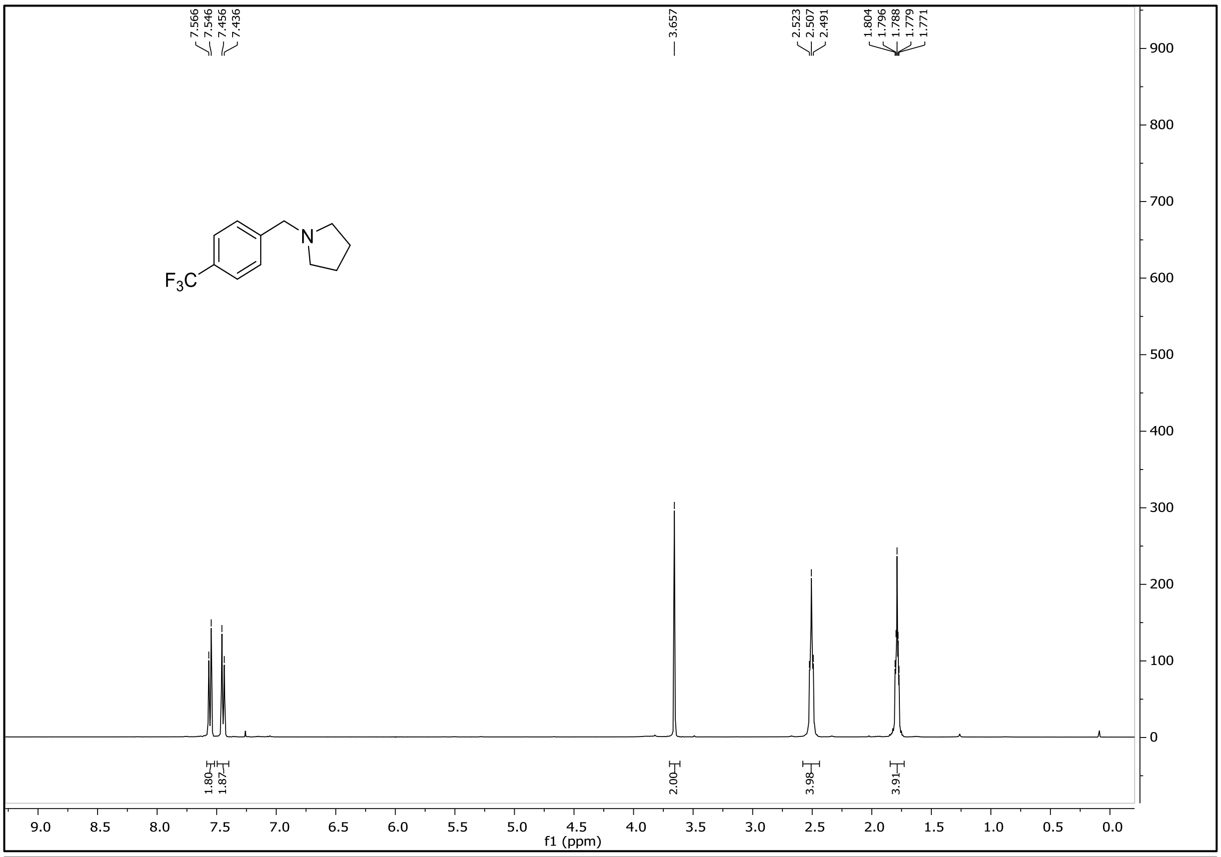

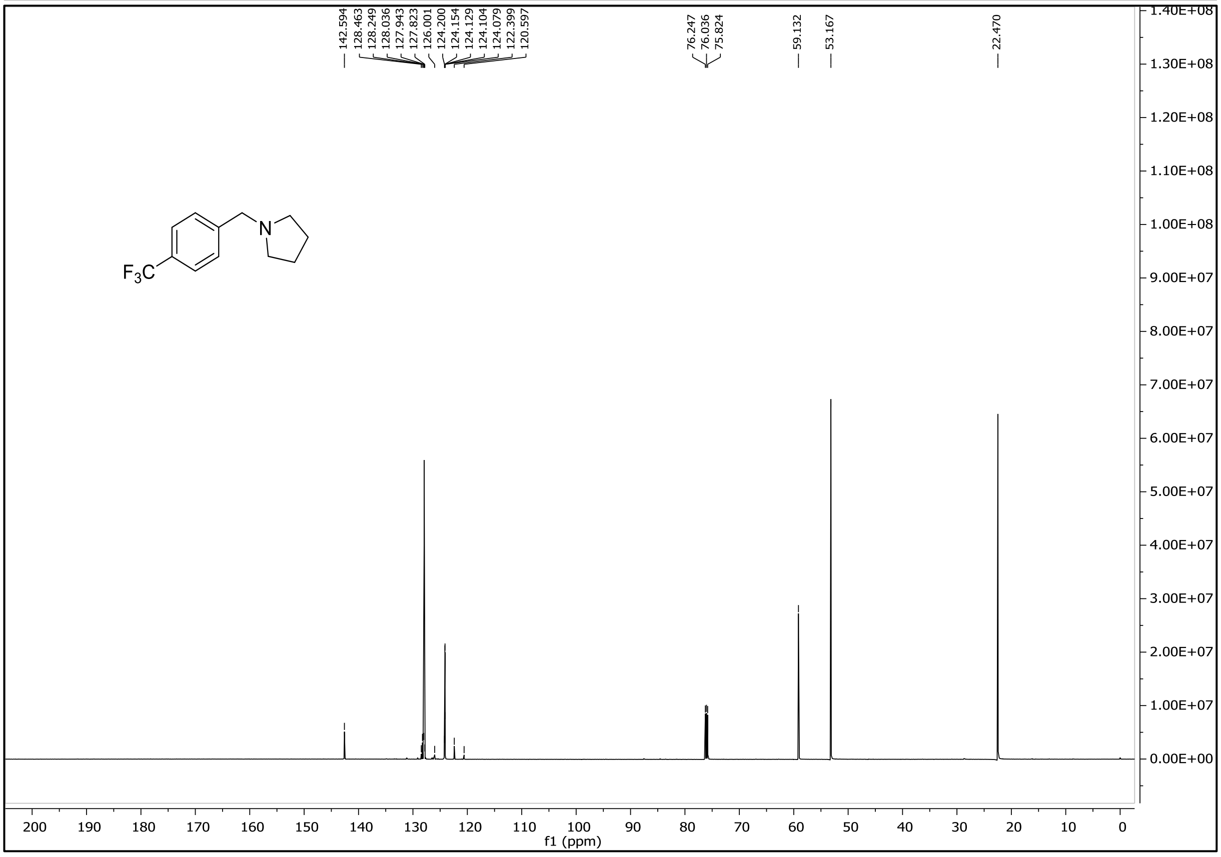

Supplementary Figure 18: ^{1}H and ^{13}C NMR of compound **3ak.**

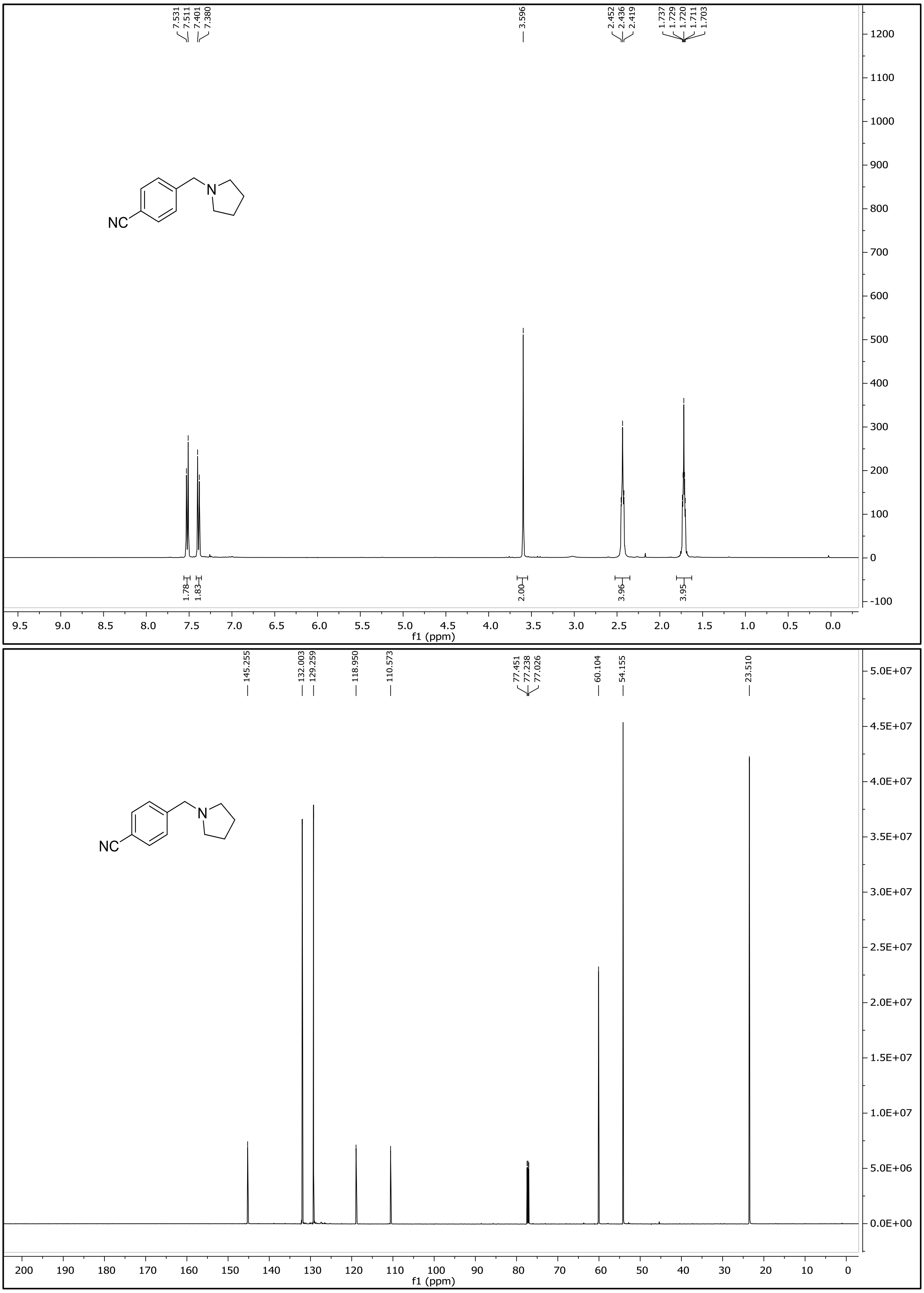

Supplementary Figure 19: ^{1}H and ^{13}C NMR of compound **3al.**

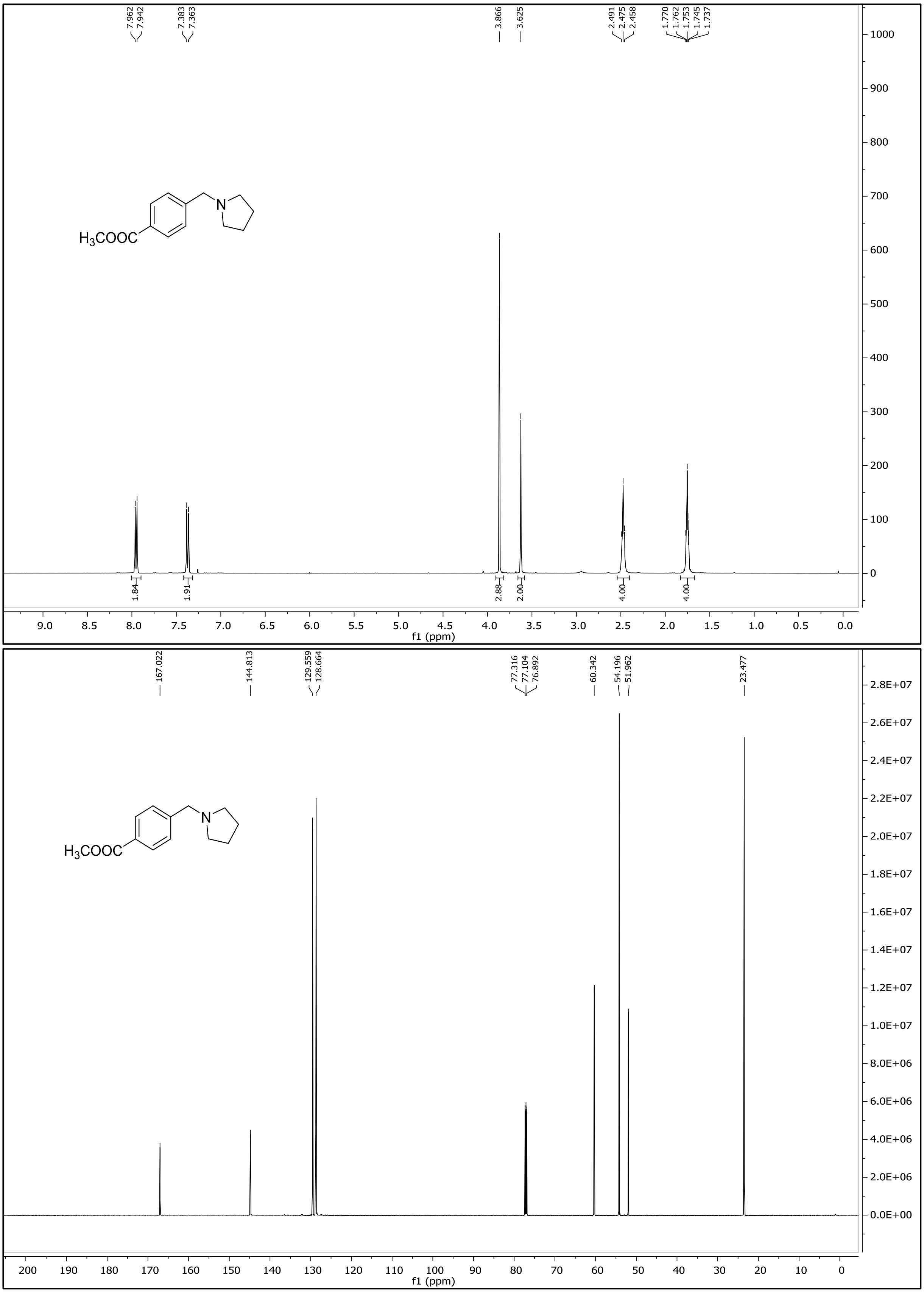

Supplementary Figure 20: ^{1}H and ^{13}C NMR of compound **3am.**

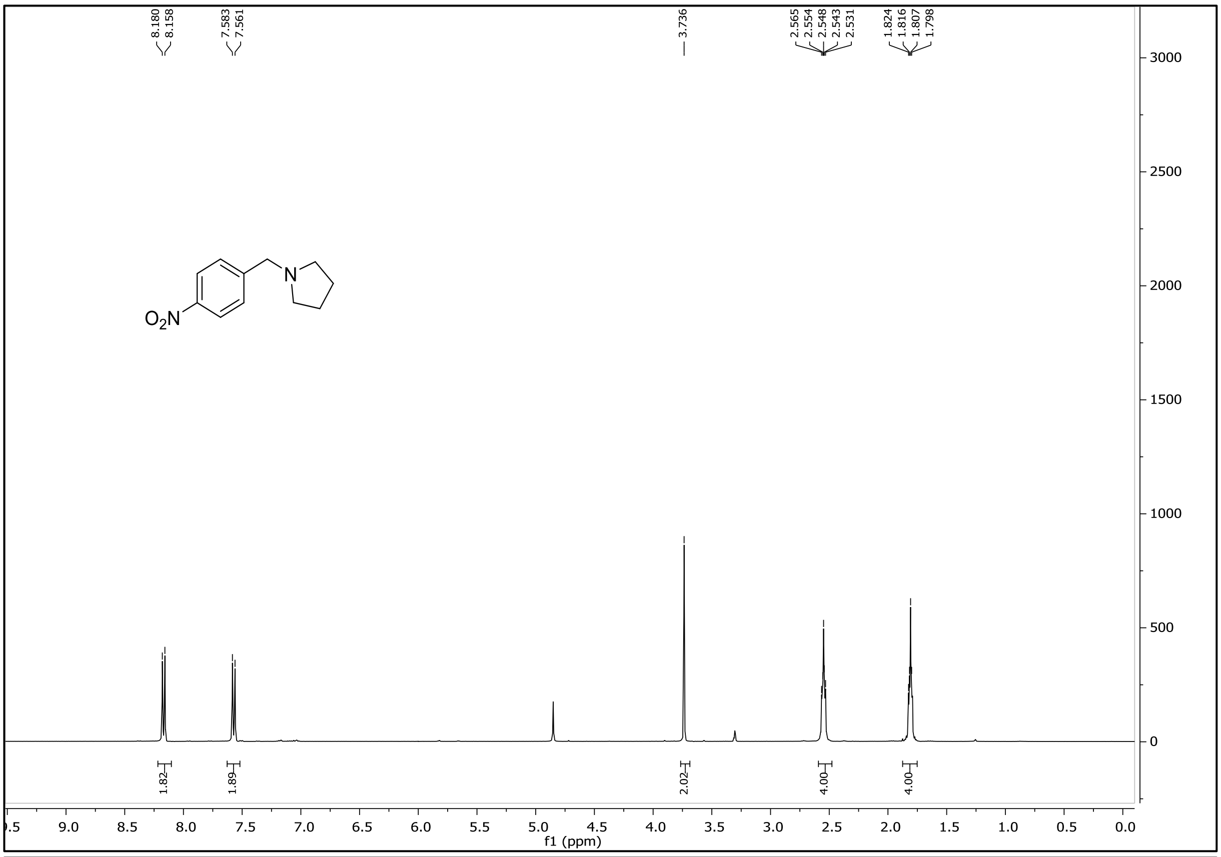

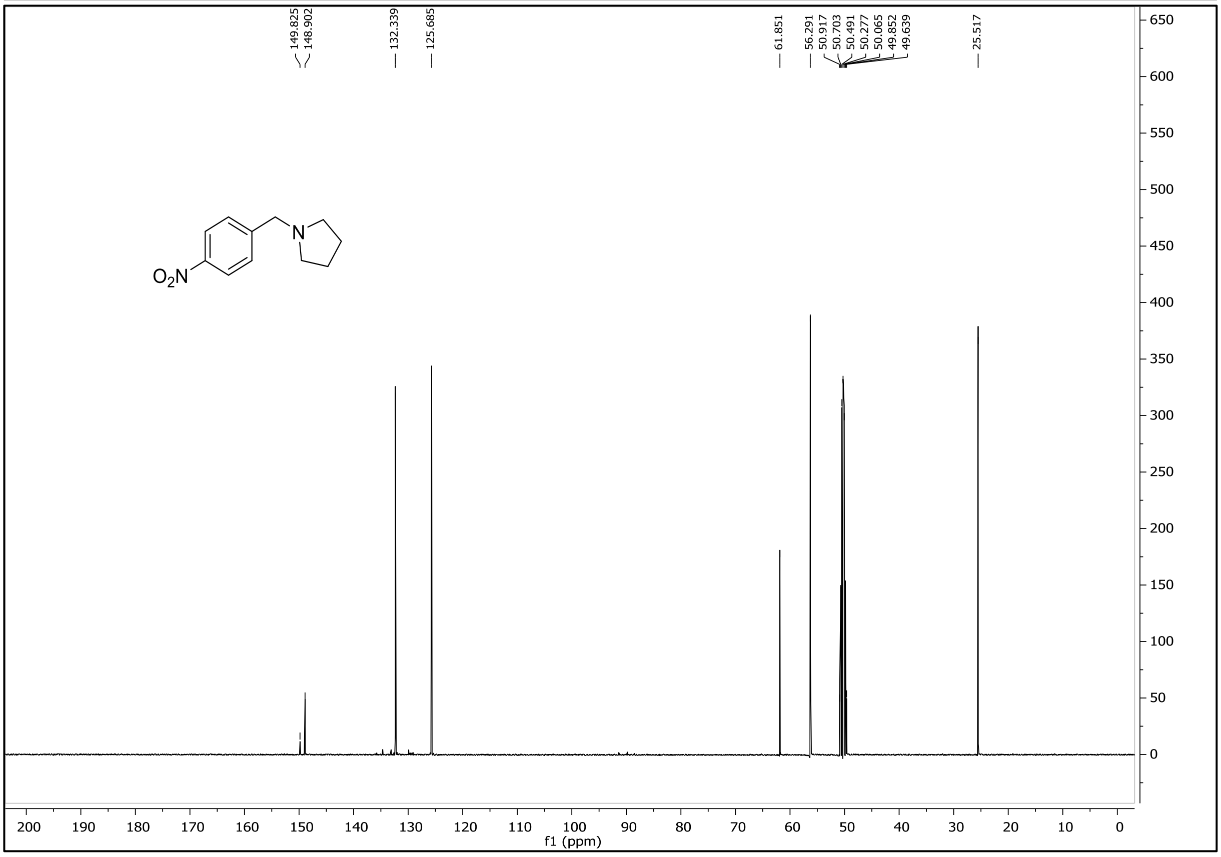

Supplementary Figure 21: ^{1}H and ^{13}C NMR of compound **3an.**

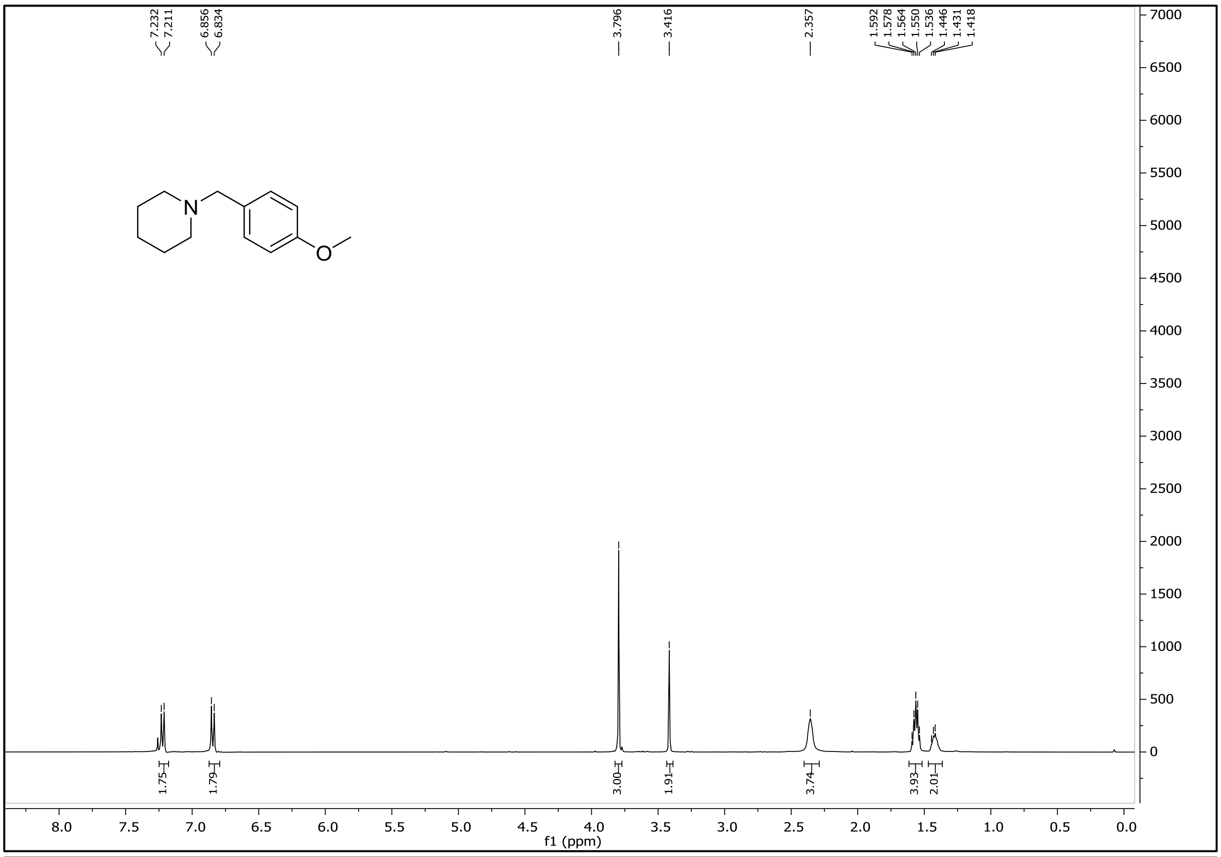

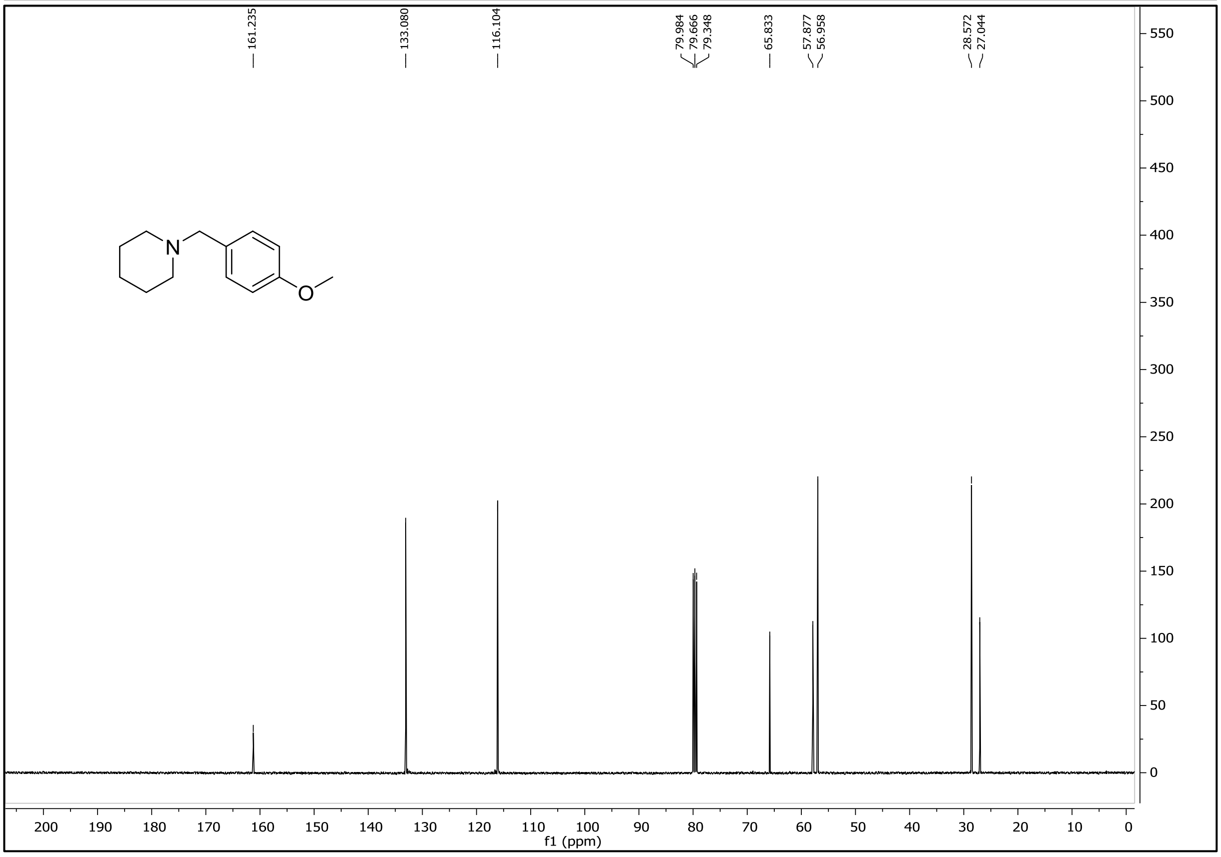

Supplementary Figure 22: ^{1}H and ^{13}C NMR of compound **3ba.**

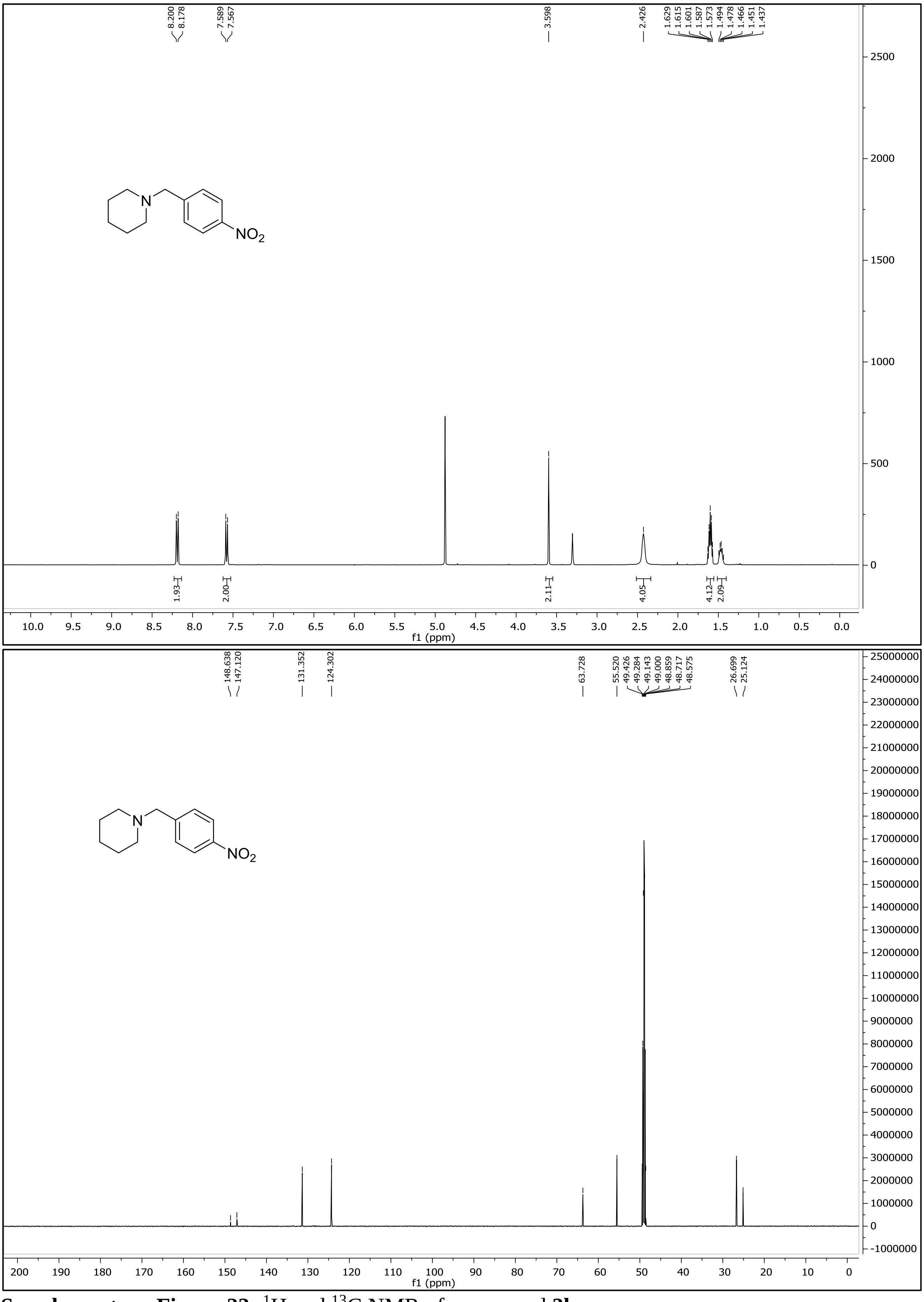

Supplementary Figure 23: ^{1}H and ^{13}C NMR of compound **3bn**.

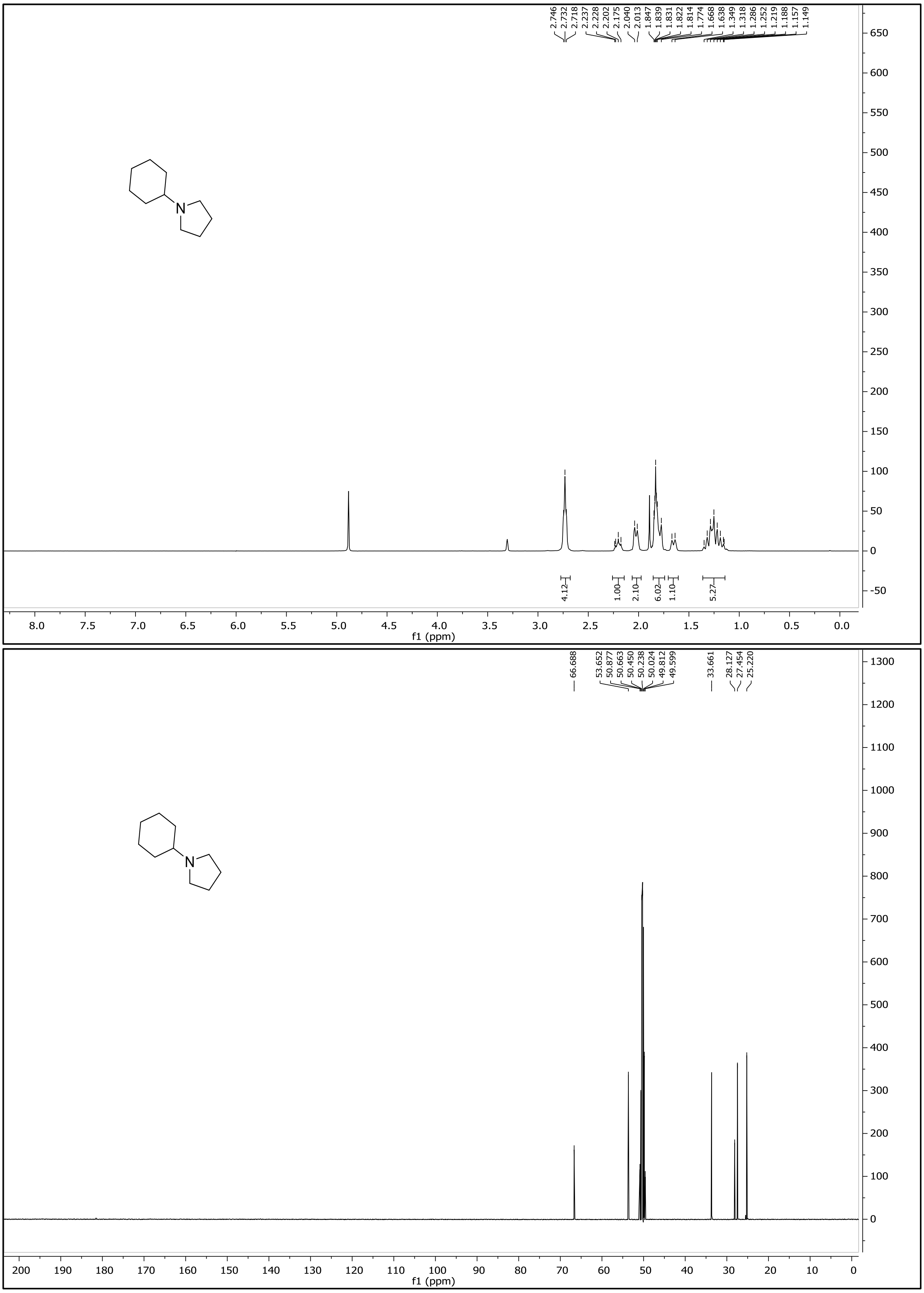

Supplementary Figure 24: ^{1}H and ^{13}C NMR of compound **3aq**.

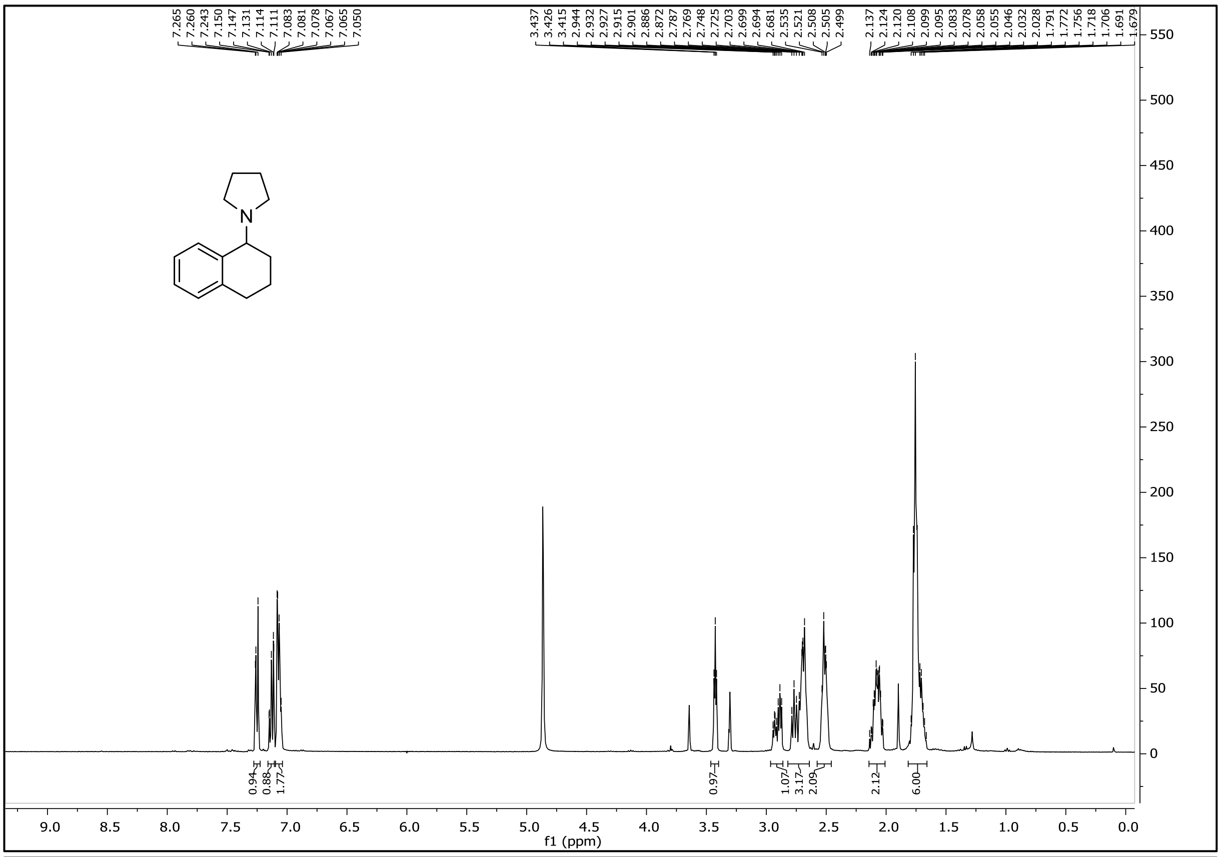

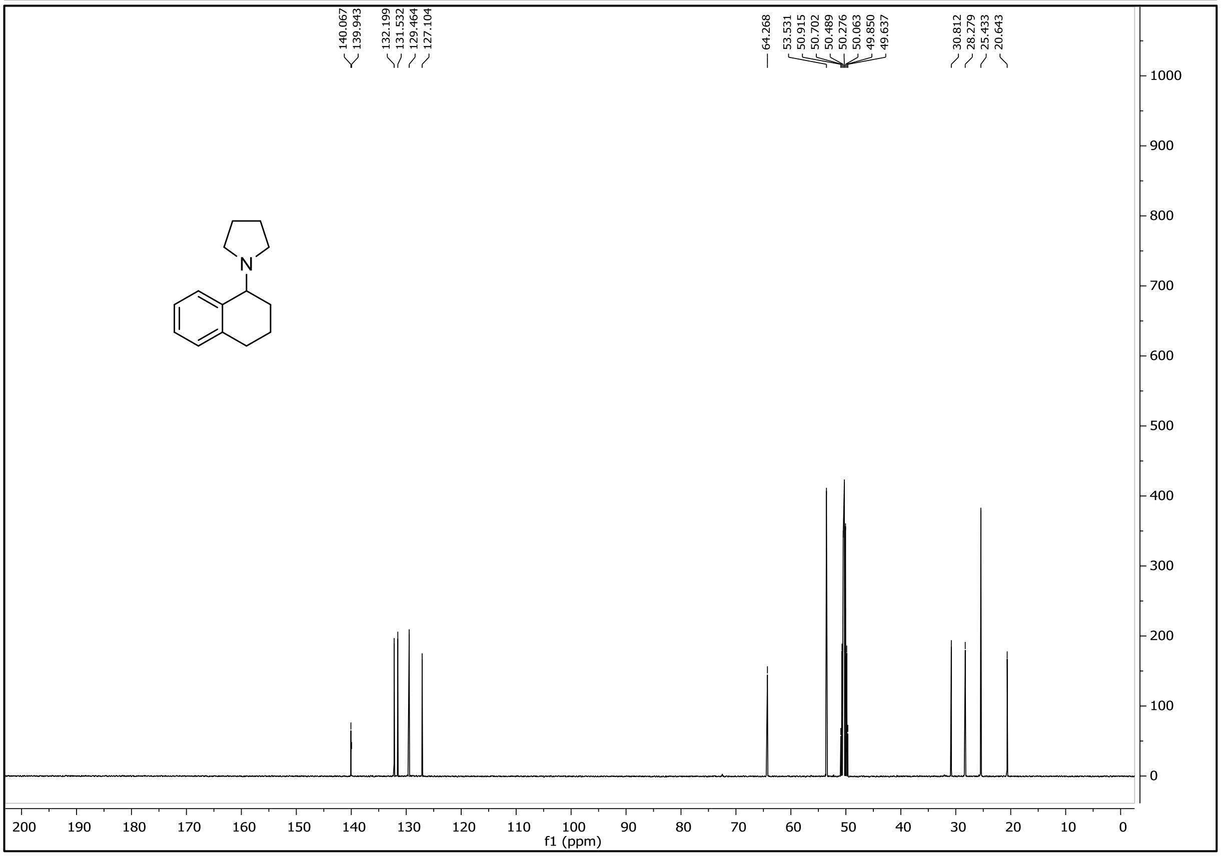

Supplementary Figure 25: ^{1}H and ^{13}C NMR of compound **3at**.

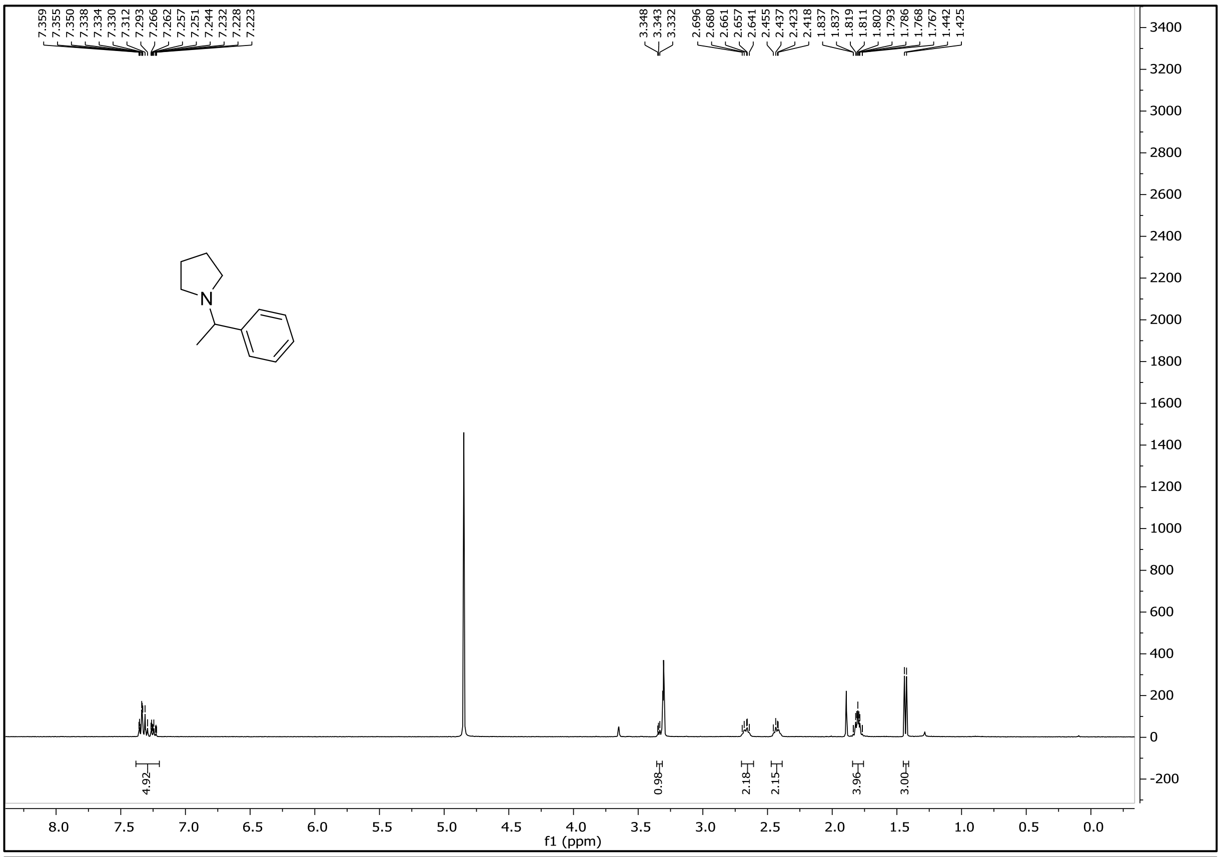

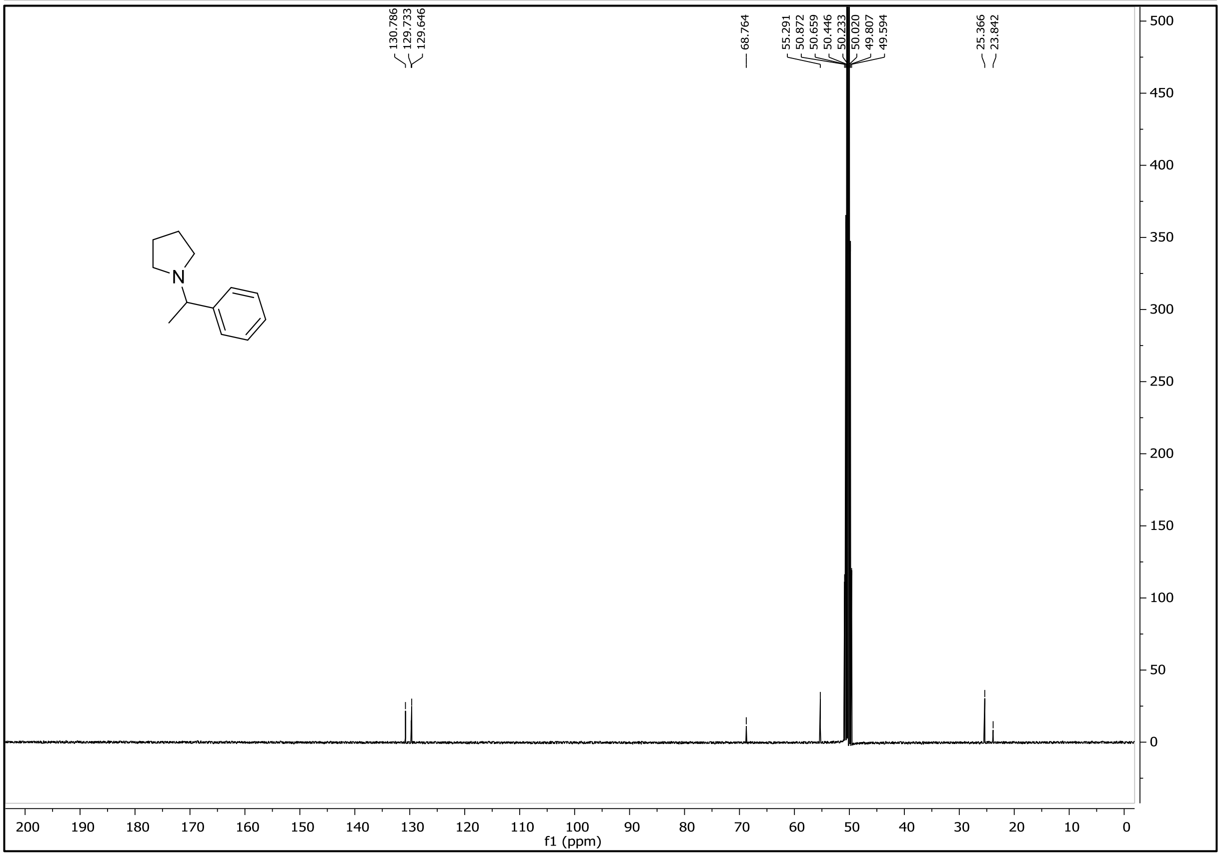

Supplementary Figure 26: ^{1}H and ^{13}C NMR of compound **3au.**

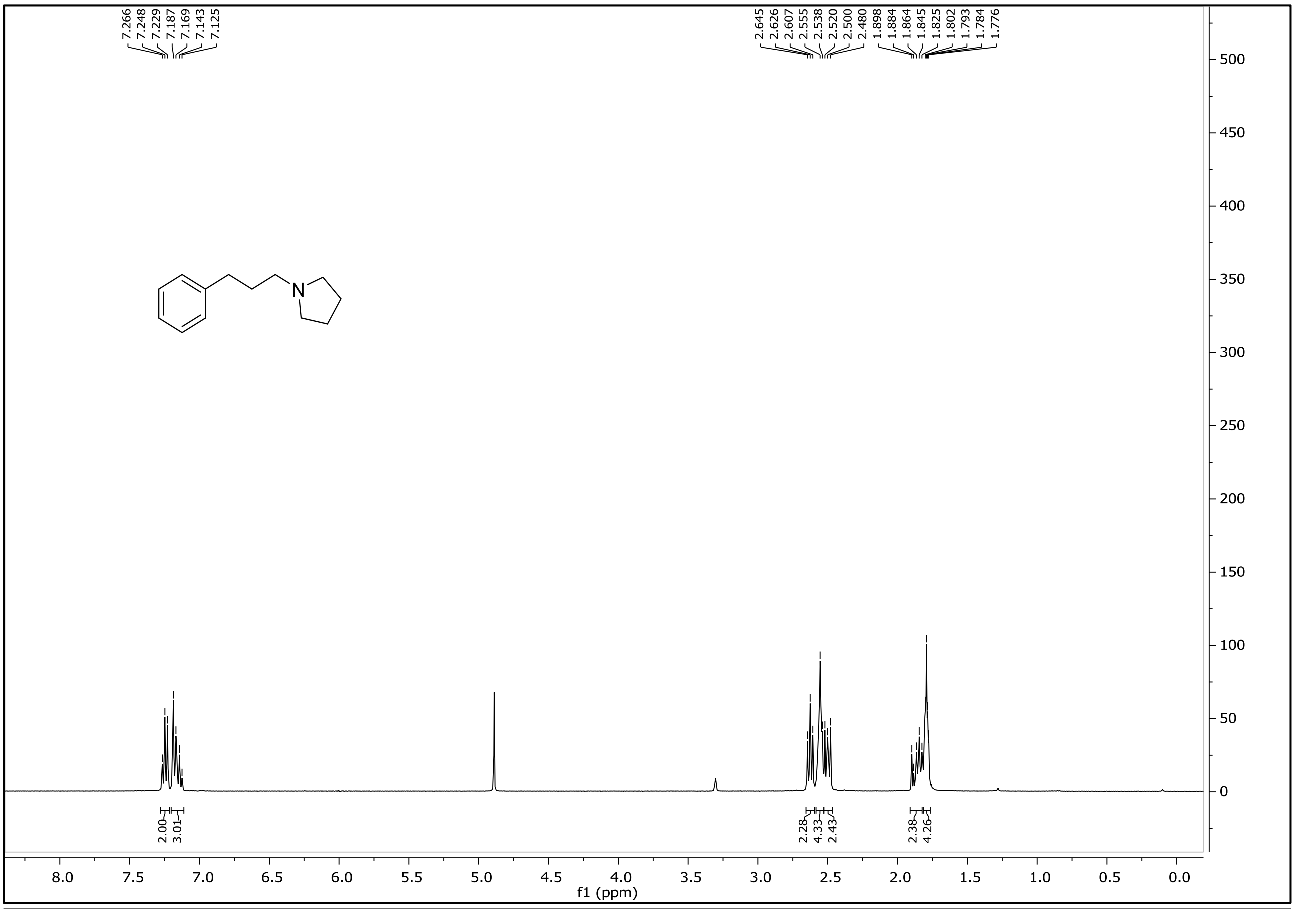

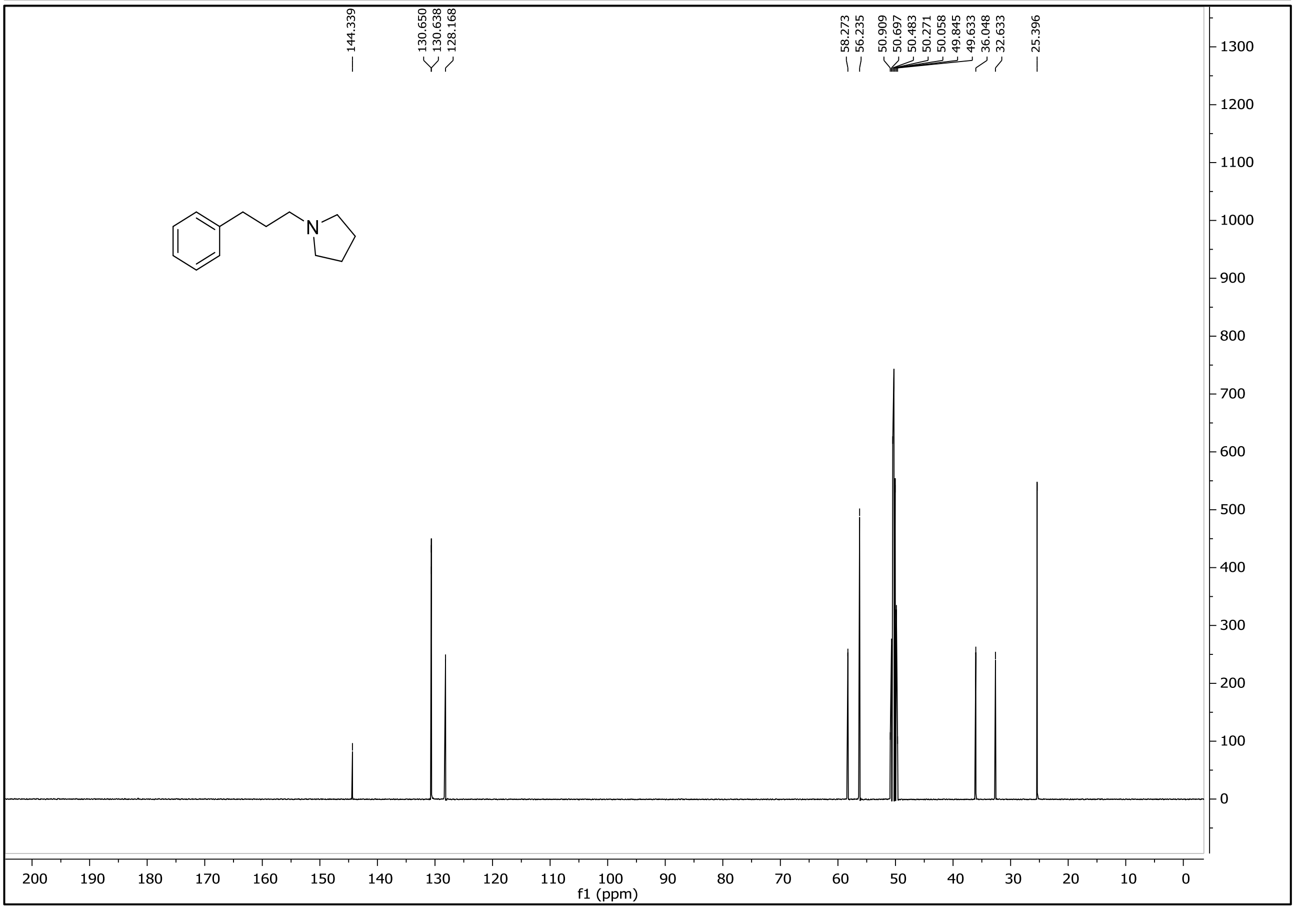

Supplementary Figure 27: ^{1}H and ^{13}C NMR of compound **3aw**.

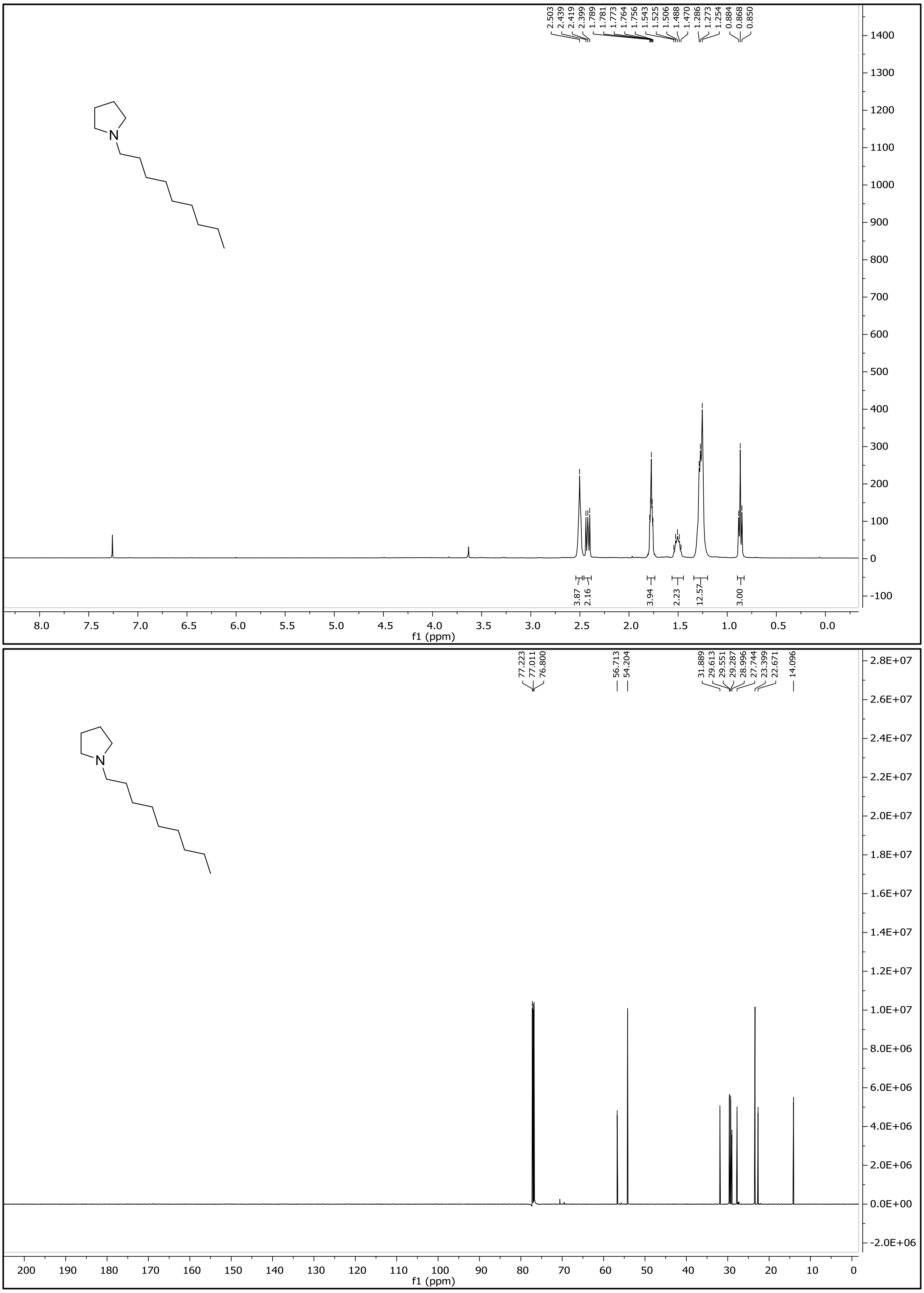

Supplementary Figure 28: ^{1}H and ^{13}C NMR of compound **3ay.**

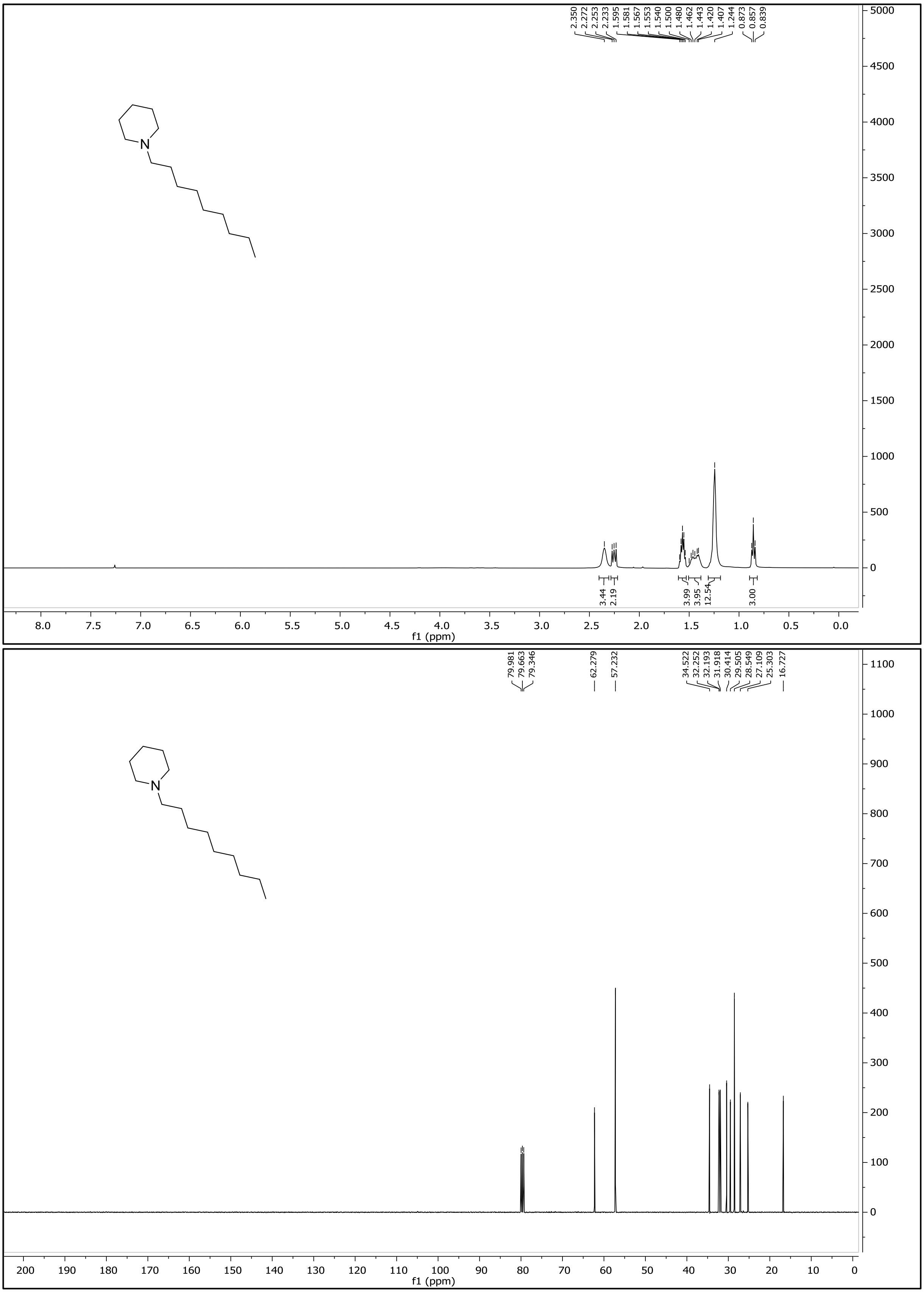

Supplementary Figure 29: ^{1}H and ^{13}C NMR of compound **3by.**

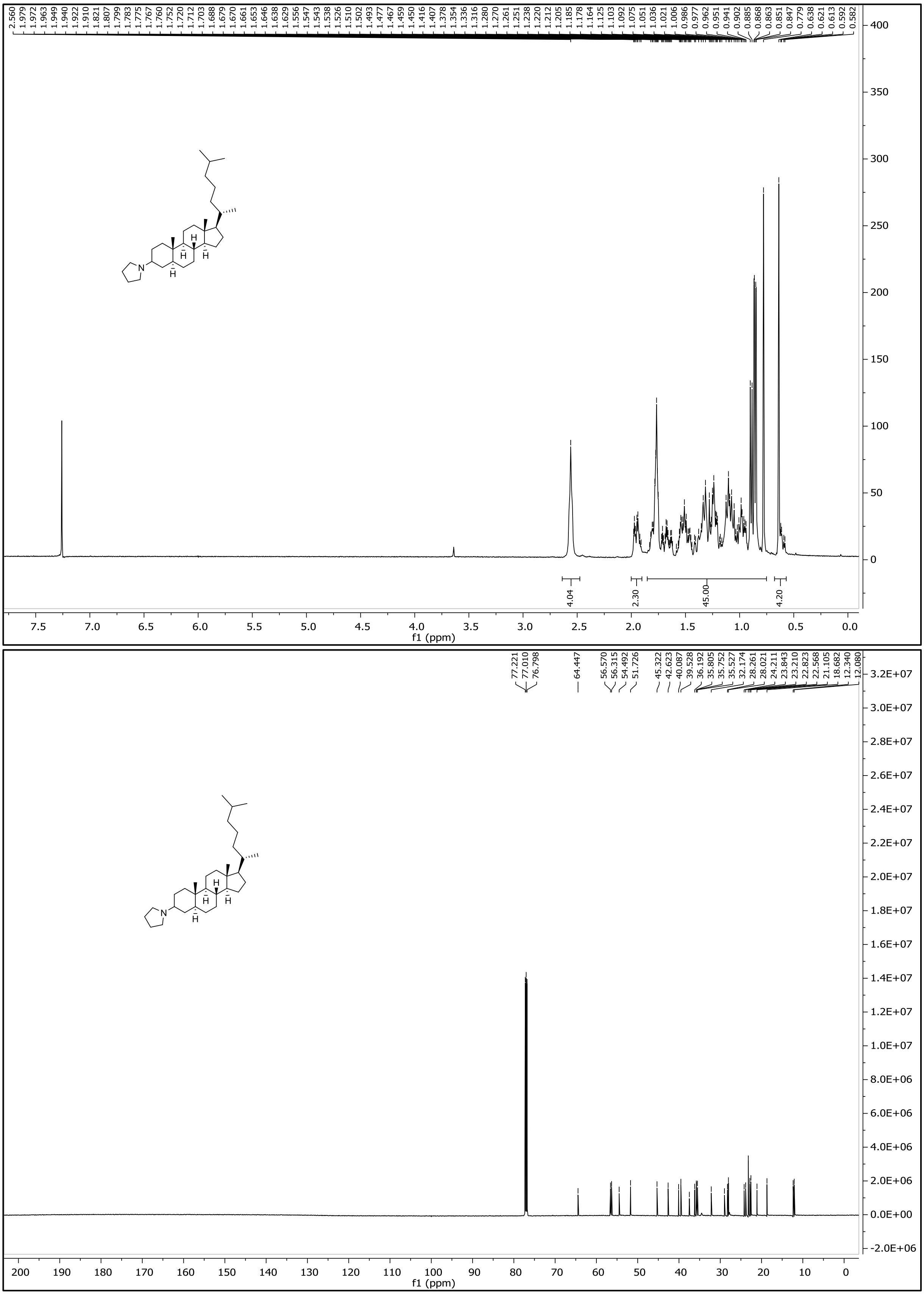

Supplementary Figure 30: ^{1}H and ^{13}C NMR of compound **3az**.

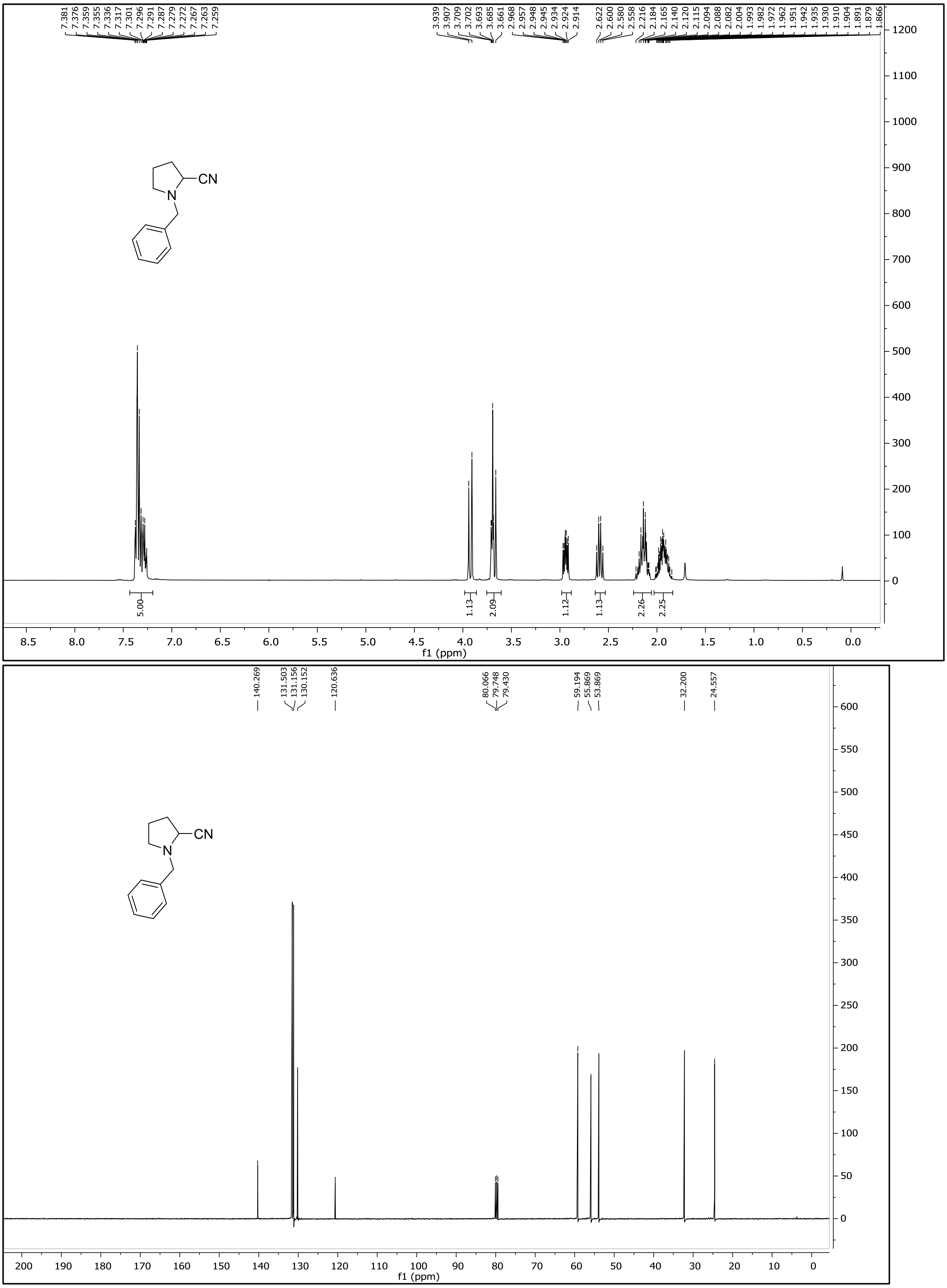

Supplementary Figure 31: ^{1}H and ^{13}C NMR of compound **5a**.

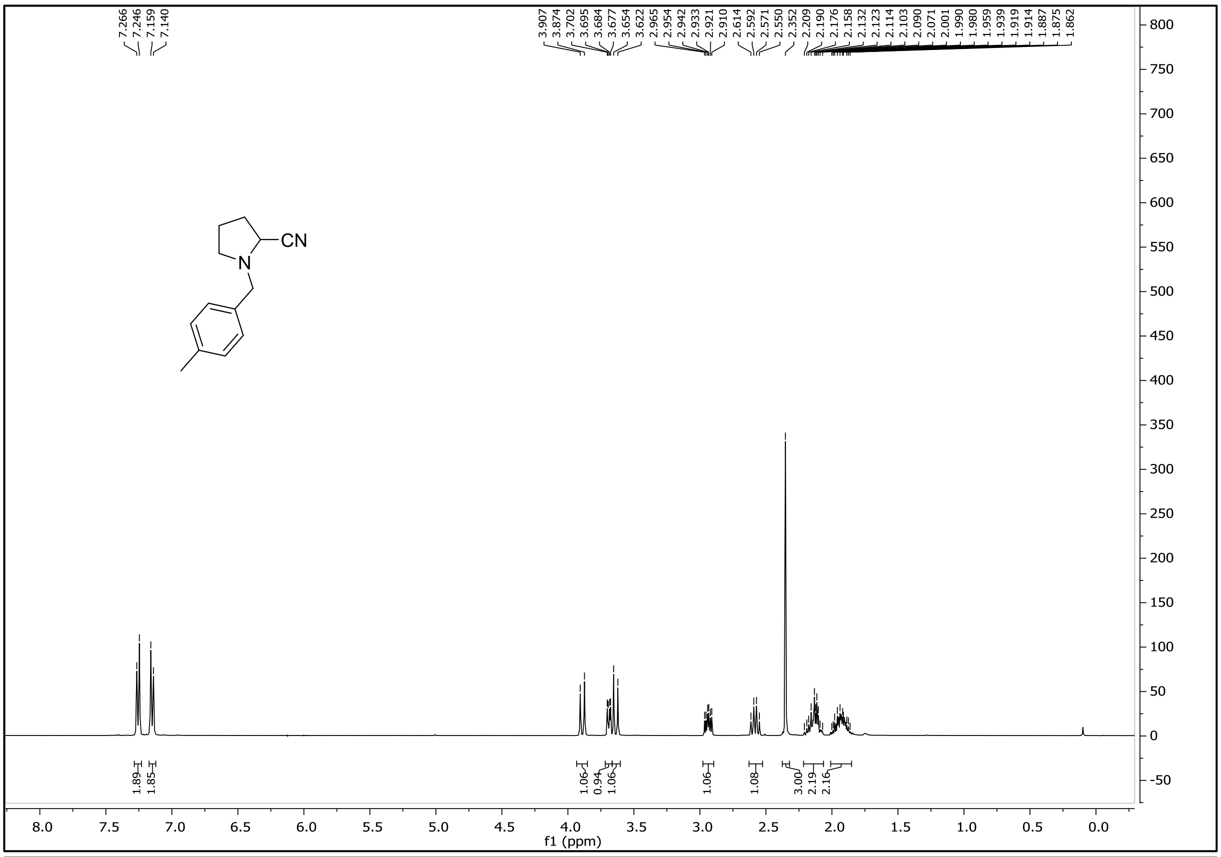

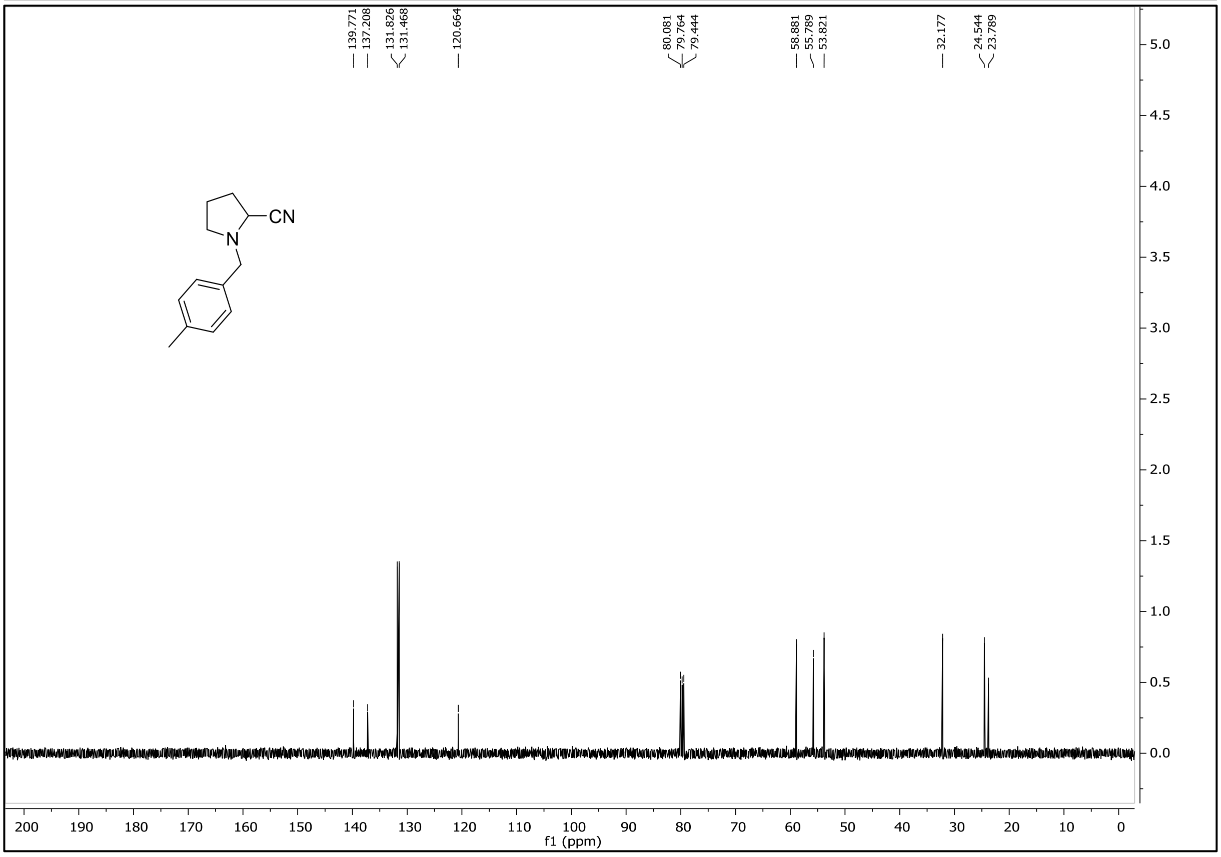

Supplementary Figure 32: ^{1}H and ^{13}C NMR of compound **5b**.

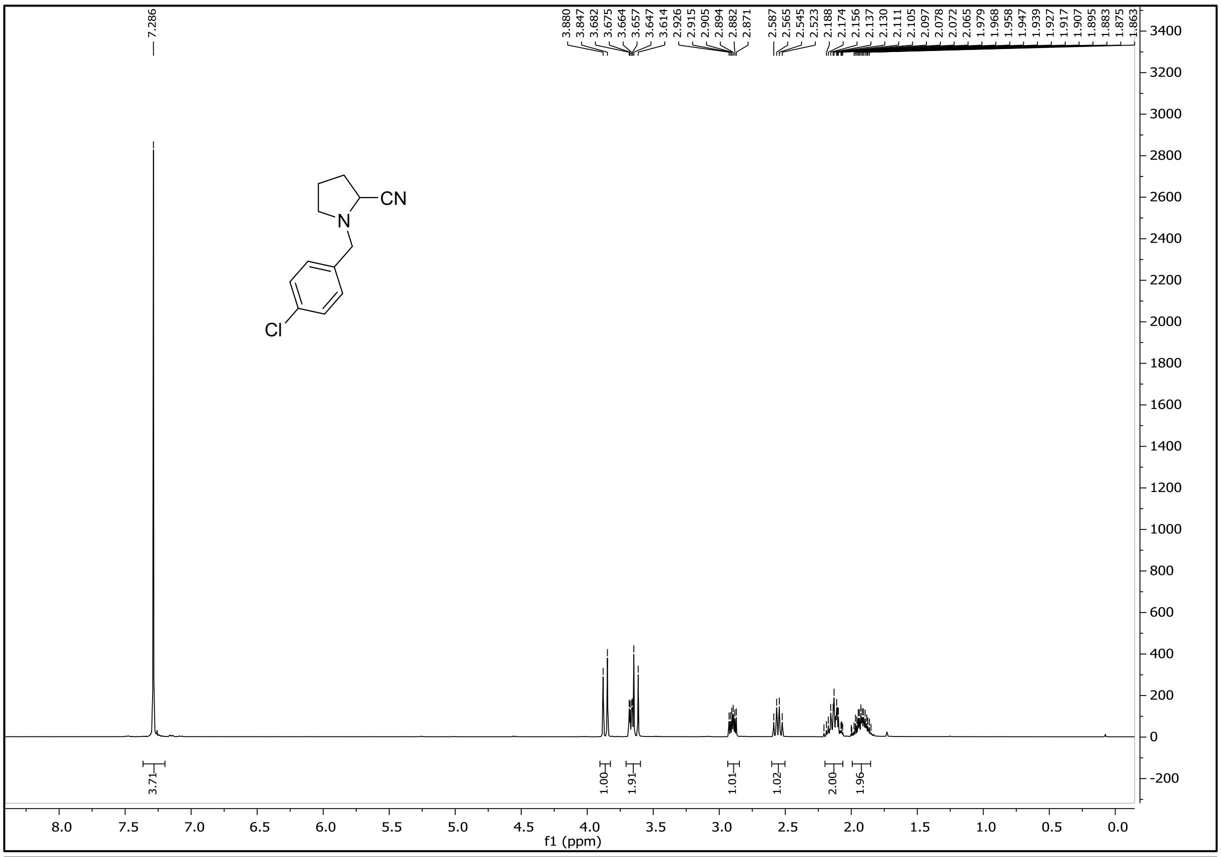

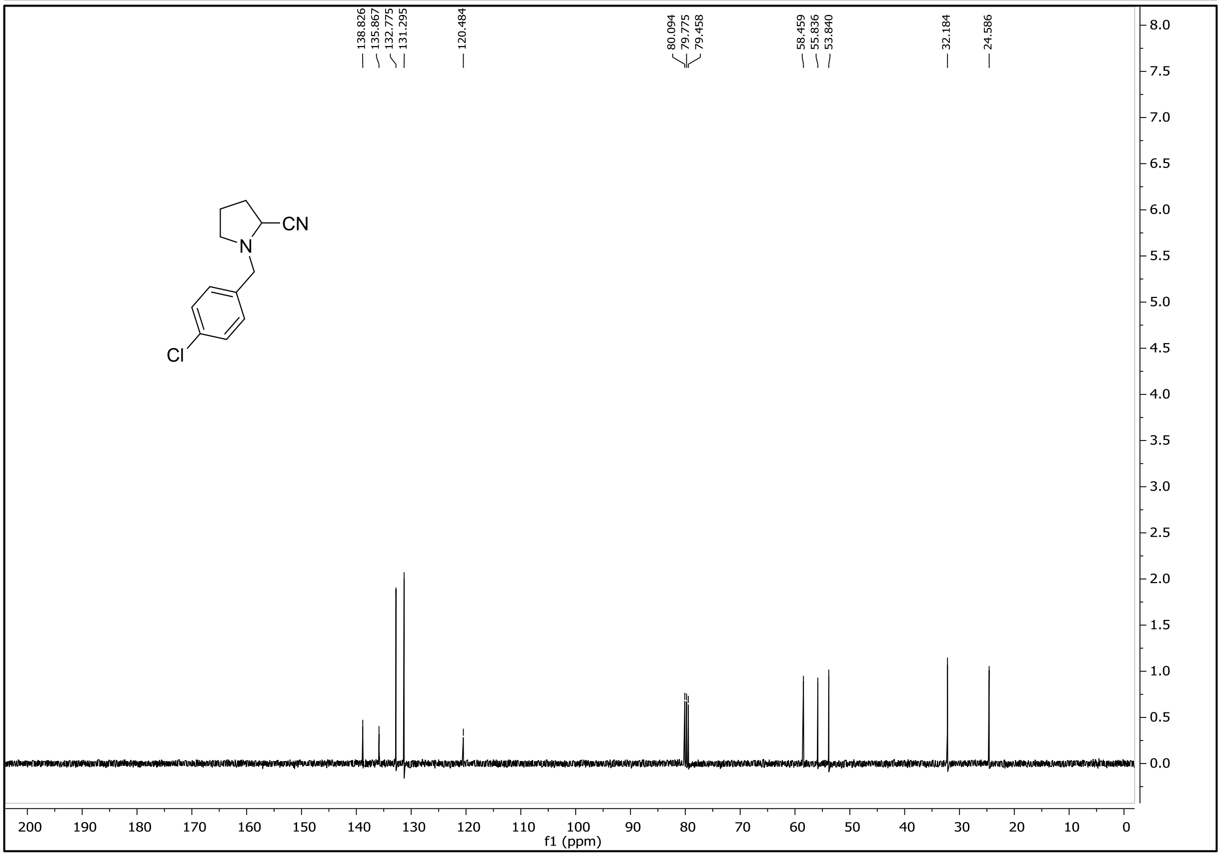

Supplementary Figure 33: ^{1}H and ^{13}C NMR of compound **5c**.

References

[1] T. N. Plank, J. L. Drake, D. K. Kim, T. W. Funk, *Adv. Synth. Catal.* **2012**, *354*, 597–601.
[2] C. Zhang, D. Seidel, *J. Am. Chem. Soc* **2010**, *132*, 1798–1799.
[3] L. Ma, W. Chen, D. Seidel, *J. Am. Chem. Soc.* **2012**, *134*, 15305–15308.
[4] G. A. Molander, P. E. Gormisky, D. L. Sandrock, *J. Org. Chem.* **2008**, *73*, 2052–2057.
[5] T. Jerphagnon, G. P. M. Van Klink, J. G. De Vries, G. Van Koten, *Org. Lett.* **2005**, *7*, 5241–5244.
[6] E. Sperotto, G. P. M. van Klink, J. G. de Vries, G. van Koten, *Tetrahedron* **2010**, *66*, 3478–3484.
[7] G. Wuitschik, M. Rogers-Evans, A. Buckl, M. Bernasconi, M. Märki, T. Godel, H. Fischer, B. Wagner, I. Parrilla, F. Schuler, et al., *Angew. Chemie - Int. Ed.* **2008**, *47*, 4512–4515.
[8] A. C. Maxwell, A. J. A. Watson, M. H. S. A. Hamid, J. M. J. Williams, H. C. Maytum, G. W. Lamb, C. L. Allen, *J. Am. Chem. Soc.* **2009**, *40*, 1766–1774.
[9] K. Fujita, F. Fujii, A. Komatsubara, Y. Enoki, R. Yamaguchi, *Heterocycles* **2007**, *74*, 673–682.
[10] Y. Chen, Y. Liu, H. Duan, W. Liao, X. Shi, J. L. Petersen, *Chem. Commun.* **2009**, 6436–6438.
[11] R. Sheng, Y. Xu, C. Hu, J. Zhang, X. Lin, J. Li, B. Yang, Q. He, Y. Hu, *Eur. J. Med. Chem.* **2009**, *44*, 7–17.
[12] Z. Sahli, B. Sundararaju, M. Achard, C. Bruneau, *Org. Lett.* **2011**, *13*, 3964–3967.
[13] M. Ahmed, R. P. J. Bronger, R. Jackstell, P. C. J. Kamer, P. W. N. M. Van Leeuwen, M. Beller, *Chem. - A Eur. J.* **2006**, *12*, 8979–8988.
[14] J. Han, B. Xu, G. B. Hammond, *Org. Lett.* **2011**, *13*, 3450–3453.
[15] D. Das, M. T. Richers, L. Ma, D. Seidel, *Org. Lett.* **2011**, *13*, 6584–6587.
